# Engineering of the *E. coli *Outer Membrane Protein FhuA to overcome the Hydrophobic Mismatch in Thick Polymeric Membranes

**DOI:** 10.1186/1477-3155-9-8

**Published:** 2011-03-17

**Authors:** Noor Muhammad, Tamara Dworeck, Marco Fioroni, Ulrich Schwaneberg

**Affiliations:** 1 Department of Biotechnology (Biology VI), RWTH Aachen University, Worringerweg 1, 52074 Aachen, Germany

## Abstract

**Background:**

Channel proteins like the engineered FhuA Δ1-159 often cannot insert into thick polymeric membranes due to a mismatch between the hydrophobic surface of the protein and the hydrophobic surface of the polymer membrane. To address this problem usually specific block copolymers are synthesized to facilitate protein insertion. Within this study in a reverse approach we match the protein to the polymer instead of matching the polymer to the protein.

**Results:**

To increase the FhuA Δ1-159 hydrophobic surface by 1 nm, the last 5 amino acids of each of the 22 β-sheets, prior to the more regular periplasmatic β-turns, were doubled leading to an extended FhuA Δ1-159 (FhuA Δ1-159 Ext). The secondary structure prediction and CD spectroscopy indicate the β-barrel folding of FhuA Δ1-159 Ext. The FhuA Δ1-159 Ext insertion and functionality within a nanocontainer polymeric membrane based on the triblock copolymer PIB_1000_-PEG_6000_-PIB_1000 _(PIB = polyisobutylene, PEG = polyethyleneglycol) has been proven by kinetic analysis using the HRP-TMB assay (HRP = Horse Radish Peroxidase, TMB = 3,3',5,5'-tetramethylbenzidine). Identical experiments with the unmodified FhuA Δ1-159 report no kinetics and presumably no insertion into the PIB_1000_-PEG_6000_-PIB_1000 _membrane. Furthermore labeling of the Lys-NH_2 _groups present in the FhuA Δ1-159 Ext channel, leads to controllability of in/out flux of substrates and products from the nanocontainer.

**Conclusion:**

Using a simple "semi rational" approach the protein's hydrophobic transmembrane region was increased by 1 nm, leading to a predicted lower hydrophobic mismatch between the protein and polymer membrane, minimizing the insertion energy penalty. The strategy of adding amino acids to the FhuA Δ1-159 Ext hydrophobic part can be further expanded to increase the protein's hydrophobicity, promoting the efficient embedding into thicker/more hydrophobic block copolymer membranes.

## Background

The *E. coli *outer membrane protein FhuA (Ferric hydroxamate protein uptake component A) is one of the largest known β-barrel protein (714 amino acids, elliptical cross section 39*46 Å), consisting of 22 antiparallel β-sheets connected by short periplasmatic turns and flexible external loops. The protein channel is closed by a cork domain (amino acids 5-159). Several crystal structures of the FhuA wild type have been resolved [1,2]. For biotechnological applications one FhuA variant has been engineered in which the cork domain has been (FhuA Δ1-159, *i.e*. deletion of amino acids 1 - 159) removed, resulting in a passive mass transfer channel [3].

The FhuA Δ1-159 variant has been inserted as a nanochannel triggered by chemical external stimuli into PMOXA-PDMS-PMOXA [PMOXA - poly(2-methyl-2-oxazoline); PDMS - poly(dimethyl-siloxane)] triblock copolymer membranes [4]. FhuA Δ1-159 Lys-NH_2 _position 556 has been found to be the most efficient in channel triggering after labeling [5].

Polymersomes, polymer vesicles/micelles self-assembled from synthetic amphiphilic block copolymers [6-8] have been shown to possess superior biomaterial properties, including greater chemical and physical stability [9,10], as compared to liposomes.

In fact these vesicles represent encapsulation devices that can be used as delivery systems, as bio-mimetic membranes, as biomedical imaging tools, as protection devices for labile substances or as nanoreactors for localized chemical reactions [11].

Polymersomes vary in size from some tens of nanometers to tens of microns. For drug delivery purposes hydrophilic compounds can be encapsulated within the vesicle interior. In contrast to liposomes, polymersomes are quite impermeable so that once encapsulated drugs can be specifically released at the target site. Commonly the release happens upon irreversible polymersome fractioning or degradation. Alternative release mechanisms involve the insertion of channel proteins into the polymer membrane [12-15].

Membranes formed by block copolymers are often thicker (5-22 nm) than those formed by "natural" phospholipids (3-4 nm) leading to better mechanical strength [7].

The aforementioned difference in membrane thickness may lead to dropped efficiency of channel insertion (comparing polymersomes and liposomes), due to the hydrophobic mismatch [16], where the hydrophobic mismatch is defined as the difference between the hydrophobic length of a membrane protein and the hydrophobic thickness of the membrane it spans.

The common strategy for the functional reconstitution of membrane proteins into polymeric membranes requires to design polymer membranes as thin and fluidal as possible, in order to minimize the energetic penalty associated with exposing a nonpolar/polar interface.

As an example: Simulation studies conducted on OmpF (outer membrane protein F) insertion into EO_29_EE_28 _(Ethyleneoxide_29_-Ethylethylene_28_) membranes show a considerable symmetric deformation of the hydrophobic region of the polymer. The hydrophobic mismatch upon insertion is 1.32 nm, corresponding to 22% of the polymer thickness [17]. As a consequence, if copolymer bilayer cannot withstand the hydrophobic mismatch, channel protein insertion is prevented [18,19].

**Figure 1:**
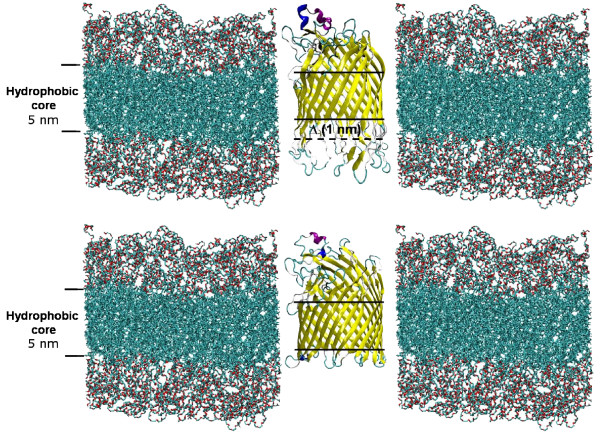
**Schematic representation of FhuA Δ1-159 Ext (top) and FhuA Δ1-159 (bottom) within triblock copolymer PIB_1000_-PEG_1500_-PIB_1000 _membranes**. Membrane structure was obtained by Molecular Dynamics calculations (see Additional File 1). The hydrophobic transmembrane regions of FhuA Δ1-159 Ext (hyrophobic portion: 4 nm) and FhuA Δ1-159 (hydrophobic portion: 3 nm) are indicated with lines; the extended part of FhuA Δ1-159 Ext is indicated by a broken line. Graphical representations obtained by VMD (Visual Molecular Dynamics program ver. 1.6, http://www.ks.uiuc.edu/Research/vmd/).

Differing from the approach of choosing/synthesizing the polymer to match the protein, we match the protein to the polymer by protein engineering. For this purpose a FhuA Δ1-159 channel protein variant with an extended hydrophobic portion (FhuA Δ1-159 Ext) was engineered by "copy-pasting" the last five amino acids of each β-strand, increasing the overall channel length from 3 nm to 4 nm thus reducing the hydrophobic mismatch (Figure 1; see also Section Methods, "Engineering, expression and extraction of FhuAΔ1-159 Ext").

The 1 nm increase of the hydrophobic protein portion is the limit to ensure the insertion of the FhuA Δ1-159 Ext into the *E. coli *membrane. A further elongation would lead to a hydrophobic mismatch between the protein and the lipid membrane forbidding the FhuA Δ1-159 Ext insertion, resulting in unfolded protein accumulation in inclusion bodies.

FhuA Δ1-159 Ext was functionally inserted into vesicles formed by BAB triblock copolymer PIB_1000_-PEG_6000_-PIB_1000 _(PIB = Polyisobutylene; PEG = Polyethylene glycol - Figure S4 Additional File 1) with a hydrophobic thickness of 5 nm for the entangled chains as derived by MD calculations (see Additional File 1) and experimental data [20].

The vesicle wall shows double bilayer morphology suggested from cryo-SEM pictures (see Additional File 1, Figure S8) and from previous experimental results based on BAB tri-block copolymers[21,22].

The advantages of choosing this triblock copolymer are that both building blocks (PIB/PEG) are highly biocompatible [23,24] with the PIB unit impermeable to many compounds and gases [25]. Additionally PIB_1000_-PEG_6000_-PIB_1000 _is commercially available and cost effective.

To our knowledge this is the first time a channel protein was specifically engineered for the purpose of insertion into PIB_1000_-PEG_6000_-PIB_1000 _type membranes. Furthermore to demonstrate the functionality of FhuA Δ1-159 Ext as a channel, kinetics for TMB (3,3',5,5'-tetramethylbenzidine) uptake by HRP (Horse Radish Peroxidase) loaded polymersomes with inserted open and biotin label-closed FhuA Δ1-159 Ext channel were measured.

## Results and Discussion

### Structure prediction and CD Spectra to verify folding of FhuA Δ1-159 Ext

The secondary/tertiary structure analysis of the FhuA Δ1-159 Ext answers whether the engineering strategy to elongate the hydrophobic portion of the protein leads still to a β-sheet folding, important for the channel functionality.

Based on the observation that the original FhuA Δ1-159 is able to independently refold after thermal denaturation (data not published), showing that folding information is fully contained within the primary sequence, a "copy-paste" strategy to double the last 5 amino acids of each of the 22 β-sheets prior to the more regular periplasmatic β-turns has been developed. The 5 pasted amino acids are expected to contain the same folding information as the copied ones in the original primary sequence. The 1 nm increase of the hydrophobic protein portion is the limit to ensure the insertion of the FhuA Δ1-159 Ext into the *E. coli *membrane. A further elongation would lead to a hydrophobic mismatch between the protein and the lipid membrane forbidding the FhuA Δ1-159 Ext insertion, resulting in unfolded protein accumulation in inclusion bodies.

The percentages of secondary structure elements as predicted by using the PSIPRED server (http://bioinf.cs.ucl.ac.uk/psipred/) are summarized in Table 1. A detailed view of the server results is given in Additional File 1 (Figure S1, S2 and S3).

**Table 1:** Percent occurrence of each secondary structure element in FhuA WT, FhuA Δ1-159 and FhuA Δ1-159 Ext.

Predicted Secondary Structure			
**Protein**	**α-helix (%)**	**β-sheet (%)**	**random coil (%)**

FhuA WT	3.3	46.1	50.6

FhuA Δ1-159	1.6	65.4	33.0

FhuA Δ1-159 Ext	5.1	59.2	35.6

Secondary structure was predicted using the PSIPRED server (http://bioinf.cs.ucl.ac.uk/psipred/).

In agreement with the server prediction results for WT, FhuA Δ1-159 and WT crystal structure [1], the predicted secondary structure of variant FhuA Δ1-159 Ext content is well retained.

The prediction of FhuA Δ1-159 Ext secondary structure leads, similarly to FhuA Δ1-159 and WT, to a high percentage of β-sheet confirming the validity of the the five amino acids addition strategy. Further corroboration by CD analysis will be reported in the paragraph "CD Spectra of FhuA Δ1-159 Ext".

### Extraction and Purification of FhuA Δ1-159 Ext

**Figure 2:**
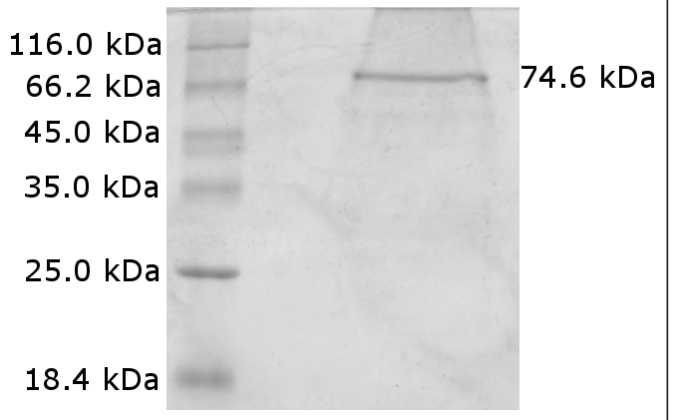
**SDS-PAGE of purified FhuA Δ1-159 Ext**. The sequence derived, expected molecular weight of FhuA Δ1-159 Ext is 74.6 kDa.

Serial extraction with organic solvents led to 250 μg/ml of FhuA Δ1-159 Ext solubilised in buffer containing OES. SDS-PAGE (Figure 2) and subsequent ImageJ (http://rsbweb.nih.gov/ij/index.html) analysis resulted in a FhuA Δ1-159 Ext purity of ~92%.

### Influx kinetics and TMB/HRP detection system

**Figure 3:**
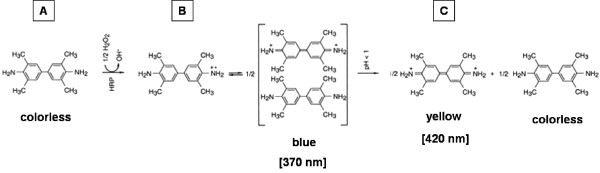
**Schematic representation of two-step TMB conversion reaction**.

TMB is widely used in enzyme immunoassays (EIA) as chromogenic substrate of the HRP. The TMB/HRP detection system is based on a two-step irreversible consecutive reaction A→B→C (A = TMB; B and C = first and second TMB oxidation products) catalyzed by HRP in presence of H_2_O_2 _(Figure 3). Since the final TMB oxidation product C is only stable under very acidic conditions (0.3 Mol/L H_2_SO_4_) [26], the intermediate product (B) is used as a reporter with a characteristic adsorbance maximum at 370 nm.

The HRP was encapsulated into polymersomes and despite of using the Soret absorption band, the total amount of encapsulated enzyme could not be detected.

The kinetic data obtained in presence of the FhuA Δ1-159 Ext, were compared to a set of negative controls to verify the obtained results. In detail: Polymersome + HRP, Polymersome + HRP + FhuA Δ1-159, Free HRP and Polymersome + HRP + detergent. Polymersome adsorption was subtracted from all kinetic data.

By blocking the inserted FhuA Δ1-159 Ext via biotinylation of the channel Lys residues, prior to nanocompartment insertion, the functionality of the channel protein could be further validated. This channel blocking approach had already been employed in previous studies based on the FhuA Δ1-159 [4,5].

**Figure 4:**
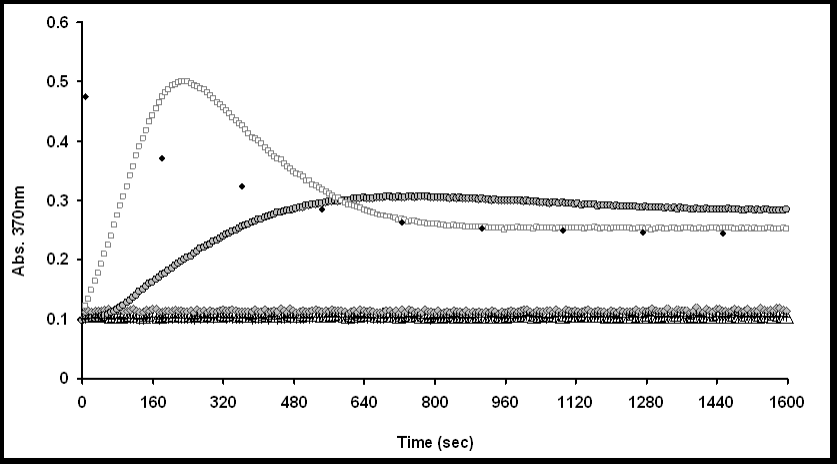
**Results of TMB conversion kinetics**. HRP loaded polymersome (triangles), HRP loaded polymersome + OES detergent (grey diamonds), HRP loaded polymersome + FhuA Δ1-159 (plus), HRP loaded polymersome + unblocked FhuA Δ1-159 Ext (squares), HRP loaded polymersome + blocked FhuA Δ1-159 Ext (grey cycles), Free HRP (black diamonds).

Overall results of the kinetic data are based on three individual data sets and are reported in Figure 4 and Table S1 (Additional File 1).

The polymersome membrane showed no TMB oxidation kinetics (Figure 4, triangles). The detergent, used to solubilise FhuA Δ1-159 Ext, itself has no effect on the polymersome membrane as no kinetics were observed (Figure 4, grey diamonds).

Similarly polymersomes in presence of the protein variant FhuA Δ1-159 show no TMB conversion (Figure 4, black minus). It should be underlined that FhuA Δ1-159 was previously inserted into polymersome membranes formed by the triblock copolymer PMOXA-PDMS-PMOXA [27], however it does not allow transport across PIB_1000_-PEG_6000_-PIB_1000 _membranes. This might be due to inability of FhuA Δ1-159 to reconstitute into the polymeric membrane or otherwise the protein might be reconstituted but burried completely within the thick polymersome wall and therefore unable to function as a channel. At the present research state it is not possible to distinguish between the two phenomena.

In contrast HRP loaded polymersomes in presence of the unblocked FhuA Δ1-159 Ext show a clear oxidation kinetic (Figure 4, squares), indicating the successful channel protein insertion into the polymer membrane. This result strongly indicates that the hydrophobic mismatch has been overcome by increasing the protein's hydrophobic surface. However to address the question whether the FhuA Δ1-159 Ext really acts as a channel or whether the observed kinetics are due to the locally perturbed polymer membrane by the presence of the protein, the channel was blocked by biotinylation of the Lys-NH_2 _groups.

Previous experiments show the ability of the labelling to efficiently close the channel, expecting no kinetics from the labelled channel compared to fast kinetics with an unlabeled one [4,5].

The HRP loaded polymersomes with blocked FhuA Δ1-159 Ext channel show a ~5 times smaller slope determined by absorbance kinetics as compared to polymersomes with the open channel (Figure 4, grey cycles) (see Figure S10 and Table S1 in Additional File 1). Residual kinetics of the biotinylated FhuA Δ1-159 Ext can be due to: a) lower efficiency of the labelling moieties to close the longer FhuA Δ1-159 Ext channel, or b) local perturbation of the polymersome membrane near to the protein rendering it slightly permeable to TMB. At the actual state of the art we cannot distinguish between the phenomena (a) and (b) (see next paragraph).

The TMB conversion by the free HRP results in fast kinetics (black diamonds) indicating that the comparatively slow conversion rate in case of polymersomes with inserted channel is not only influenced by the enzyme speed but is also, as expected, a diffusion limited process. In all three cases (free HRP, polymersome + open channel, polymersome + blocked channel) the reaction endpoint is the same showing the reproducibility of the HRP based detection system. Furthermore due to the absorption overlap of 1^st ^and 2^nd ^product at 370 nm the absorption does not reach to zero (see absorption scan of 2^nd ^product; Figure S9 and kinetic model discussion within Additional File 1).

In conclusion the chemical kinetics absence in presence of FhuA Δ1-159 compared to the observed TMB conversion in presence of the FhuA Δ1-159 Ext variant, clearly confirms the validity of the engineering concept proposed.

### Quantitative determination of the biotinylated Lys (biotinylation assay)

To understand how many Lys present in the FhuA Δ1-159 Ext are effectively labelled can provide first inside into the residual kinetics observed with the biotinylated FhuA Δ1-159 Ext. Therefore the biotin amount after protein labelling was determined.

**Figure 5:**
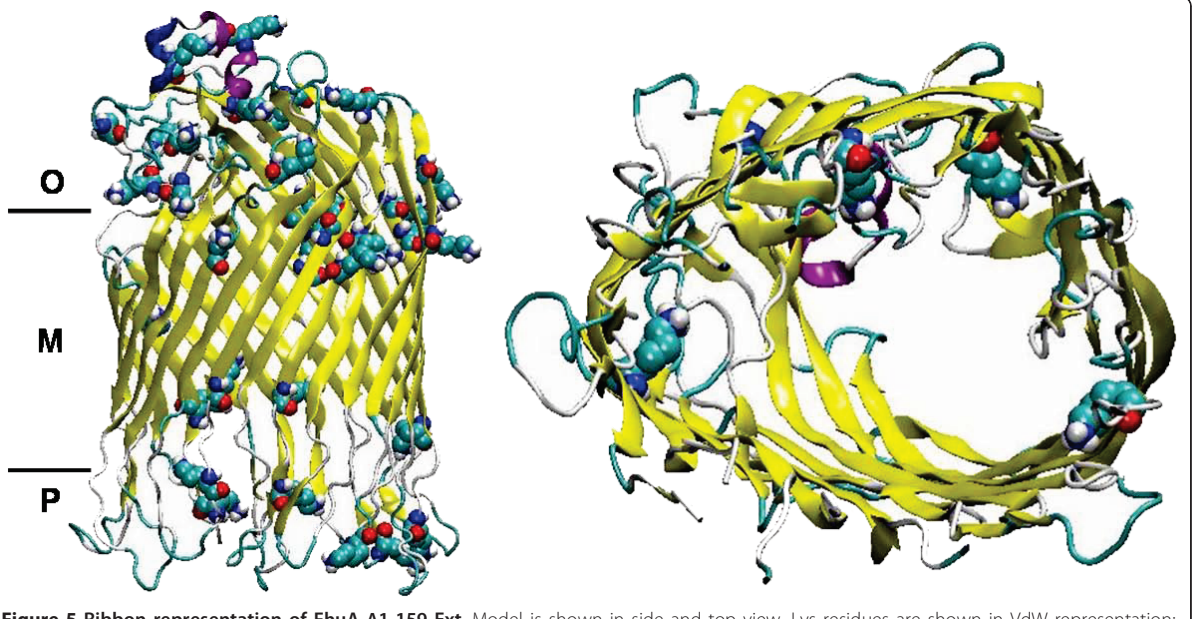
**Ribbon representation of FhuA Δ1-159 Ext**. Model is shown in side and top view. Lys residues are shown in VdW representation; side view: **O **- outer part, **M **- intermembrane part, **P **- periplasmatic part; top view: only Lys present in the channel (4) are shown. Graphical representations obtained by VMD (Visual Molecular Dynamics program ver. 1.6, http://www.ks.uiuc.edu/Research/vmd/).

FhuA Δ1-159 Ext is harboring 29 Lys residues in total. Seven of these are involved in closing the FhuA Δ1-159 Ext channel upon labelling: four are buried within the channel and three are present on both channel entrances (see Figure 5).

An average biotin concentration of ~3900 pmol was found after protease degradation (to expose all biotin moieties) of labelled FhuA Δ1-159 Ext, corresponding to the expected biotin concentration with all 29 Lys labelled (see paragraph "Biotinylation Assay" in Additional File 1). This result shows that all Lys within the channel are labelled and the observed residual flux through the polymersome membrane is not caused by low labelling efficiency.

### CD Spectra of FhuA Δ1-159 Ext

The deconvolved dichroic spectra using the CONTIN method (X) report a 75% β-sheet, 5% random coil and 20% α-helical content for the FhuA Δ1-159 Ext respectively (dichroic spectrum and fitting error are shown in Figure S14 in Additional file 1; complete fitting output is reported in Additional file 2).

To check the stability of FhuA Δ1-159 Ext after biotinylation, further CD measurements have been performed and deconvolution lead to a 0% α-helix, 58% β-sheet and 42% random coil content, (dichroic spectrum and fitting error are shown in Figure S15 in Additional file 1; complete fitting output is reported in Additional file 3).

The amount of β-structure derived by CD measurements for FhuA WT and FhuA Δ1-159 are 51% and 49% respectively [4,28].

In order to understand the secondary structure of the FhuA Δ1-159 Ext after reconstitution into polymersome membranes, the corresponding column fractions were used for CD measurements. However due to the low protein concentration within the polymersome fraction, CD signal could be only be reported after 10 fold concentration of the samples. As reported in Figure S16, the shape of the spectra strongly suggest a β-barrel structure with a representative maximum at 196 nm and a broad minimum centered at 215-220 nm (dichroic spectrum and fitting error are shown in Figure S16 in Additional file 1; complete fitting output is reported in Additional file 4).

Summing up both PSIPRED server predicted and CD derived results concerning the FhuA Δ1-159 Ext secondary structure confirm the β-barrel folding, supporting the functionality of the protein as nanochannel.

## Conclusions

Polymersomes are powerful nano-sized containers with various applications. Since block copolymer membranes are rather thick as compared to the lipid membrane found in nature, the insertion of channel proteins into polymer vesicles is limited by the hydrophobic mismatch [16]. The conventional and rather inflexible approach to overcome this limitation is to synthesise block copolymers with a chain length close to the length of membrane lipids.

In this research article a new approach for the successful insertion of the channel protein FhuA into polymersome membranes is reported. To our knowledge this is the first time a channel protein was specifically engineered for the purpose of insertion into PIB_1000_-PEG_6000_-PIB_1000 _(PIB = Polyisobutylene; PEG = Polyethylene glycol) type membranes. The advantages of choosing this triblock copolymer are that both building blocks (PIB/PEG) are highly biocompatible [23,24] with the PIB unit impermeable to many compounds and gases [25]. Additionally PIB_1000_-PEG_6000_-PIB_1000 _is commercially available and cost effective.

Differing from the approach of choosing the polymer to match the protein, we match the protein to the polymer.

A simple "copy-paste" strategy to double the last 5 amino acids of each of the 22 β-sheets prior to the more regular periplasmatic β-turns has been developed, resulting in protein variant FhuA Δ1-159 Ext (Extended). The pasted 5 amino acids are expected to bring the same folding information as the original ones.

As a consequence the protein's hydrophobic transmembrane region was increased by 1 nm, leading to a predicted lower hydrophobic mismatch between the protein and polymer membrane, minimizing the insertion energy penalty.

The 1 nm increase of the hydrophobic protein portion is the limit to ensure the insertion of the FhuA Δ1-159 Ext into the *E. coli *membrane. An increased hydrophobic mismatch between the protein and the lipid membrane would forbid the FhuA Δ1-159 Ext insertion, resulting in unfolded protein accumulation in inclusion bodies.

FhuA Δ1-159 Ext was functionally inserted into vesicles formed by triblock copolymer PIB_1000_-PEG_6000_-PIB_1000 _with a hydrophobic thickness of 5 nm for the entangled chains.

Both the secondary structure prediction analysis and CD spectroscopy, suggest the correct β-barrel folding of the engineered FhuA Δ1-159 Ext. This indicates that massive protein engineering (addition of 110 amino acids) is possible with the FhuA Δ1-159 without loosing channel functionality.

In addition we believe that our strategy of adding amino acids to the FhuA Δ1-159 Ext hydrophobic part can be further expanded to increase the protein's hydrophobicity, promoting the efficient embedding into thicker/more hydrophobic block copolymer membranes.

A further approach already under development in our Laboratory is applied to increase the channel diameter by adding 2 or more further β-sheets (FhuA Δ1-159 Exp. - Expanded) or to optimize the passive diffusion by cutting the external flexible loop domain leading to a more regular channel structure (FhuA Δ1-159 Reg. - Regular). Combination of the previous variants will give rise to a new extensive set of synthetic channels.

In the future combined approaches of matching FhuA Δ1-159 Ext to block copolymers and vice versa might complement each other synergistically, broadening the possible applications of resulting polymersomes.

## Methods

All chemicals used were of analytical grade or higher and purchased from Sigma-Aldrich Chemie (Taufkirchen, Germany) and Applichem (Darmstadt, Germany) if not stated otherwise. Protein concentrations were determined using the standard BCA kit (Pierce Chemical Co, Rockford, USA). The 2-Hydroxyethyloctylsulfoxide (OES) detergent used to solubilise the protein from the membrane was obtained from BACHEM (Switzerland).

### Engineering, expression and extraction of FhuA Δ1-159 Ext

**Figure 6:**
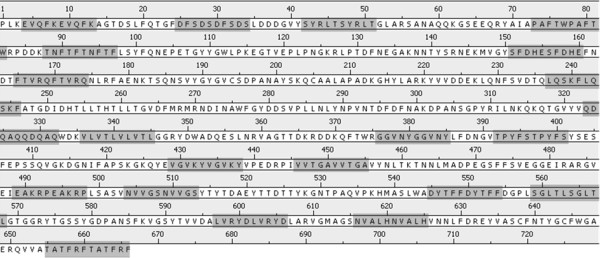
**Amino acid sequence of FhuA Δ1-159 Ext**. Copy-pasted sequence regions (5+5 amino acids) are marked in dark gray.

In order to increase the hydrophobic portion of the FhuA Δ1-159 Ext, the last five amino acids of each β-sheet prior to the periplasmatic region (110 additional amino acids in total), were copied and pasted within the primary sequence of the protein (Figure 6). The loops connecting the β-sheets remained untouched.

The corresponding synthetic gene was obtained from GeneArt (ISO 9001, Germany) and cloned into *E. coli *expression vector pET22b^+ ^(Novagen). FhuA Δ1-159 Ext variant was expressed as previously described [27] using *E. coli *BE strain BL 21 (DE3) omp8 (F- *hsdSB *(rB- mB-) *gal ompT dcm *(DE3) Δ*lamB ompF*::Tn*5 *Δ*ompA *Δ*ompC*) [29]. To extract the protein from the membrane, the membrane fraction was isolated by differential centrifugation as described [27]. Due to the hydrophobic nature of the protein it was not possible to solubilise it from the membrane directly, by adding buffer containing detergent. Instead it was necessary to extract the lipid fraction with a mixture of chloroform:methanol (3:1) to partially remove the more hydrophilic proteins, while the target protein remained within the lipid fraction. To further strip the lipid fraction from proteins more hydrophilic than the FhuA Δ1-159 Ext, it was treated with TFE:Chloroform as described [30]. Finally the residual lipid fraction was incubated with buffer containing 0.5% of the detergent OES to solubilise the target protein and the remaining membrane fraction was removed by centrifugation (45 min, 12°C, 109760 rcf; Beckmann Coulter Optima™, L-100-XP Ultracentrifuge, California USA).

The purified FhuA Δ1-159 Ext was loaded onto a 12% SDS acrylamide gel [31]. After electrophoresis the protein was stained by Coomassie Brilliant blue R-250.

### FhuA Δ1-159 Ext labelling and nanocompartment formation

A 20% DMSO aqueous solution containing (2-[Biotinamido]ethylamido)-3,3'-dithiodipropionic acid N-hydroxysuccinimide ester) (8.2 mM) was added drop-wise to a solution of FhuA Δ1-159 Ext (100 μL) and stirred (3000 rpm, 1 h; RCT basic IKAMAG, IKA-Werke GmbH, Staufen, Germany). The latter solution was used for the formation of nanocompartments loaded with HRP (2.9 U/ml). ABA (PIB_1000_-PEG_6000_-PIB_1000_) triblock copolymer (10 mg; Mw ~8000 g/mol) was dissolved in THF (100 μl; 99.8%) by 10 min vortexing. The clear solution was added drop-wise into potassium phosphate buffer (0.1 M, pH 7.4) containing HRP and stirred (3000 rpm; ambient temperature; 3 h). Nanocompartments loaded with HRP (2.9 U/ml) harbouring FhuA Δ1-159 Ext (0.13 μM final concentration) as well as amino group labelled FhuA Δ1-159 Ext (0.13 μM final concentration) were prepared by slowly dropping the polymer solution (in THF) into buffer containing FhuA Δ1-159 Ext. Resulting mixtures were stirred (3000 rpm; ambient temperature; 3 h). Nanocompartments formed by self-assembly were subsequently purified by gel filtration using Sepharose 6B in 0.1 M potassium phosphate buffer, pH 7.4.

### TMB assay with nanocompartments

The TMB (Sigma Cat. N°: T 0440) assay was selected as a conversion reporter system. Readymade TMB/H_2_O_2 _solution was used in the kinetic measurement [26,32]. The oxidation of TMB by the HRP (Horse Radish Peroxidase)/H2O2 system yields a blue first and a yellow colored second reaction product. Initial TMB oxidation kinetics were quantified by measuring an absorption maximum at 370 nm using a microtiter plate reader (Tecan Spectrofluorometer Infinite^® ^M1000, Tecan Group Ltd., Männedorf, Switzerland). TMB solution (10 μl) was supplemented to a 100 μl dispersion consisting of purified nanocompartments in potassium phosphate buffer (0.1 M, pH 7.4) in 96 well microtiter plates (Greiner flat bottom, transparent).

To measure the uptake kinetics, polymersomes with inserted FhuA Δ1-159 Ext were loaded with HRP and further purified by gel filtration. Sample fractions subjected to HRP kinetics measurement were selected on the basis of their average vesicle size (250 to 300 nm) as determined by (Malvern Z-sizer Nano ZS, UK) (see Figure S5, S6 and S7 in Additional File 1).

### Channel Blocking-Deblocking Chemistry

**Figure 7:**
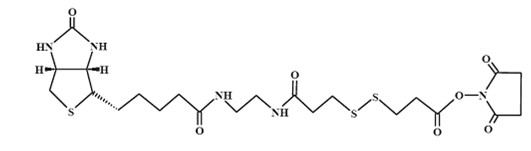
**Structure of 2-[biotinamido]ethylamido-3,3'-dithiodipropionic acid N-hydroxysuccin-imide ester**.

The blocking and deblocking chemistry was carried out as described before [4]. The selected NHS ester derivative was 2-[biotinamido]ethylamido-3,3'-dithiodipropionic acid N-hydroxysuccinimide ester (Figure 7).

### Quantitative determination of the biotinylated Lys (biotinylation assay)

The determination of the biotinyl groups present on the FhuA Δ1-159 Ext protein has been performed using the Invitrogen FluoReporter^® ^Biotin Quantitation Assay Kit specifically developed for proteins. Fluorescence spectra were detected by a Tecan Spectrofluorometer (Infinite^® ^M1000, Tecan Group Ltd., Männedorf Switzerland).

### Secondary structure prediction and CD Spectra of FhuA Δ1-159 Ext

Secondary structure of FhuA Δ1-159 Ext was predicted using the PSIPRED server (http://bioinf.cs.ucl.ac.uk/psipred/) [33]. To evaluate server performance the structures of FhuA Δ1-159 and FhuA WT (wild type) were used as standard reference.

Circular dichroism (CD) spectra were carried out for newly engineered FhuA Δ1-159 Ext to get an insite into the protein secondary structure. The spectra were obtained using the OLIS 17 DSM CD spectrometer (Olis, Bogart, USA) and Hellma^® ^SUPRASIL^® ^QS cuvettes (Hellma GmbH & Co. KG, Müllheim, Germany) with a pathlength of 0.5 mm. All measurements were performed with the FhuA Δ1-159 Ext variant solubilised in presence of phosphate buffer (0.1 M pH = 7.4), OES detergent or polymersomes.

The deconvolution of CD data was carried out by using the CONTIN algorithm [34] implemented in the Dichroprot software [35].

## Competing interests

The authors declare that they have no competing interests.

## Authors' contributions

NM and TD carried out design and performed study, data analysis and drafting of the manuscript. MF designed research. US contributed to write the paper. All authors read and approved the final manuscript.

## Supplementary Material

Additional file 1 **Engineering of the *E. coli *Outer Membrane Protein FhuA to overcome the Hydrophobic Mismatch in Thick Polymeric Membranes**. prediction analysis using PSIPRED server for secondary structure of protein, the chemical structures of polymer blocks, PIB and PEG, Polymersome DLS data, Cryo-TEM image of the polymersome, HRP assay for the second product formation, consecutive reaction analysis, biotynilation analysis for protein, molecular dynamics of PIB_1000_PEG_6000_PIB_1000 _and some CD results for FhuA Δ1-159 Ext. Click here for file

Additional file 2 **Deconvolution analysis of FhuA Δ1-159 Ext (unlabelled) in octyl-pOE (detergent)**. CD spectra deconvolution analysis by the CONTIN algorithm of the FhuA Δ1-159 Ext (unlabelled) in octyl-pOE (detergent) solution. Click here for file

Additional file 3 **Deconvolution analysis of labelled FhuA Δ1-159 Ext in octyl-pOE (detergent)**. CD spectra deconvolution analysis by the CONTIN algorithm of the FhuA Δ1-159 Ext (labelled) in octyl-pOE (detergent) solution. Click here for file

Additional file 4 **Deconvolution analysis of the FhuA Δ1-159 Ext in Polymersomes**. CD spectra deconvolution analysis by the CONTIN algorithm of the FhuA Δ1-159 Ext in poylmersomes. Click here for file
