# Supplementary material for: Engineering of the E. coli Outer Membrane Protein FhuA to overcome the Hydrophobic Mismatch in Thick Polymeric Membranes
Source: J Nanobiotechnology. 2011 Mar 17;9:8. doi: 10.1186/1477-3155-9-8 (PMC3064644; doi:10.1186/1477-3155-9-8)
Supplement: Additional file 1 — Engineering of the E. coli Outer Membrane Protein FhuA to overcome the Hydrophobic Mismatch in Thick Polymeric Membranes. prediction analysis using PSIPRED server for secondary structure of protein, the chemical structures of polymer blocks, PIB and PEG, Polymersome DLS data, Cryo-TEM image of the polymersome, HRP assay for the second product formation, consecutive reaction analysis, biotynilation analysis for protein, molecular dynamics of PIB1000PEG6000PIB1000 and some CD results for FhuA Δ1-159 Ext. [file 1477-3155-9-8-S1.PDF]

Additional File 1

Engineering of the *E. coli* Outer Membrane Protein FhuA to overcome the Hydrophobic Mismatch in Thick Polymeric Membranes

Noor Muhammad[1], Tamara Dworeck[1], Marco Fioroni[1, §] and Ulrich Schwaneberg[1, §]

[1]Department of Biotechnology (Biology VI), RWTH Aachen University, Worringerweg 1, 52074 Aachen, Germany

[§]Corresponding author

Email addresses:
NM: n.muhammad@biotec.rwth-aachen.de
TD: t.dworeck@biotec.rwth-aachen.de
MF: m.fioroni@biotec.rwth-aachen.de
US: u.schwaneberg@biotec.rwth-aachen.de

Content

The PSIPRED server secondary structure prediction	**S03**
Tri-block copolymer structures of the PIBSA and PEG	**S05**
Polymersomes DLS Data	**S05**
Cryo TEM	**S08**
HRP Assay	**S10**
Consecutive Reaction Analysis	**S12**
Biotinylation Assay	**S14**
Molecular Dynamics PIB_{1000}-PEG_{1500}-PIB_{1000}	**S15**
CD Results	**S18**

The PSIPRED server secondary structure prediction

The PSIPRED server (http://bioinf.cs.ucl.ac.uk/psipred/) was used to predict secondary structure of analyzed FhuA variants.

Figure S1. FhuA Δ1-159 Ext secondary structure prediction result.

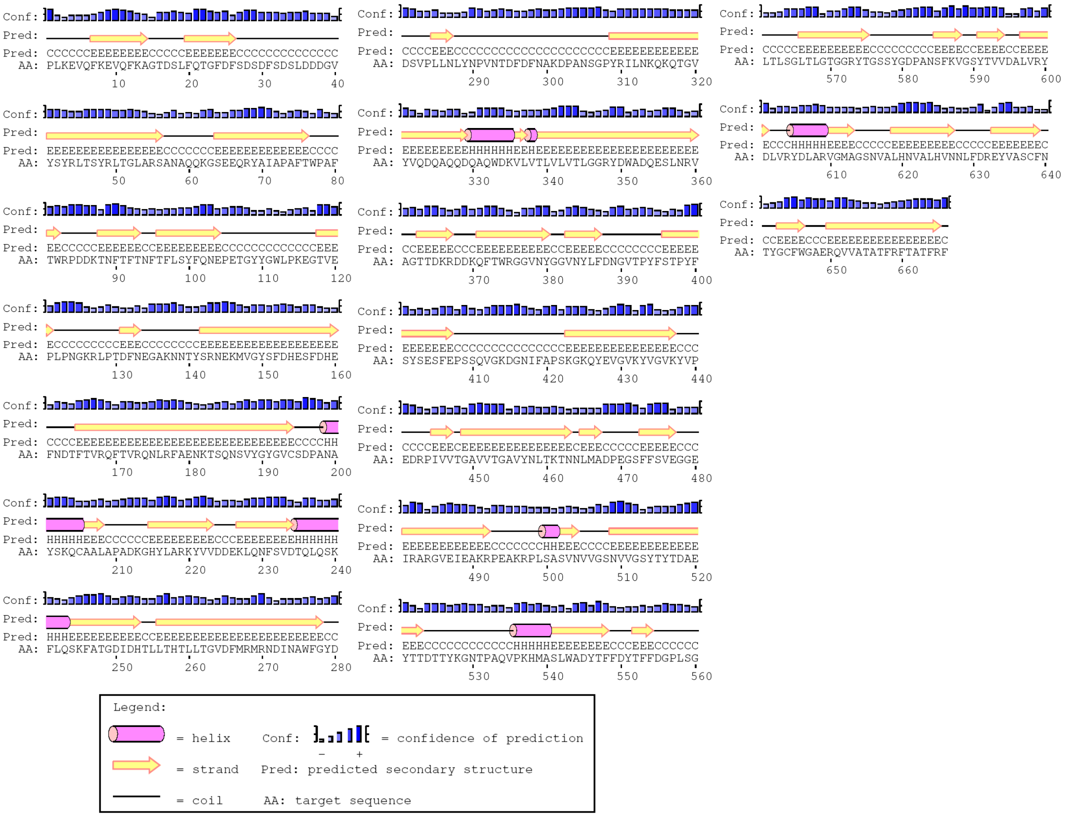

Figure S2. FhuA Δ1-159 secondary structure prediction result.

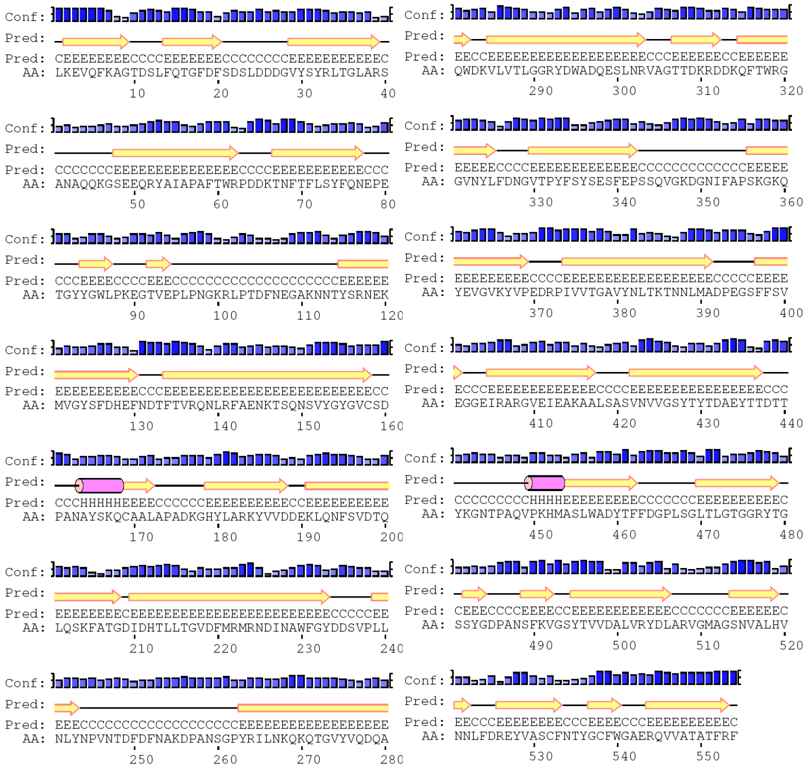

Figure S3. FhuA WT secondary structure prediction result.

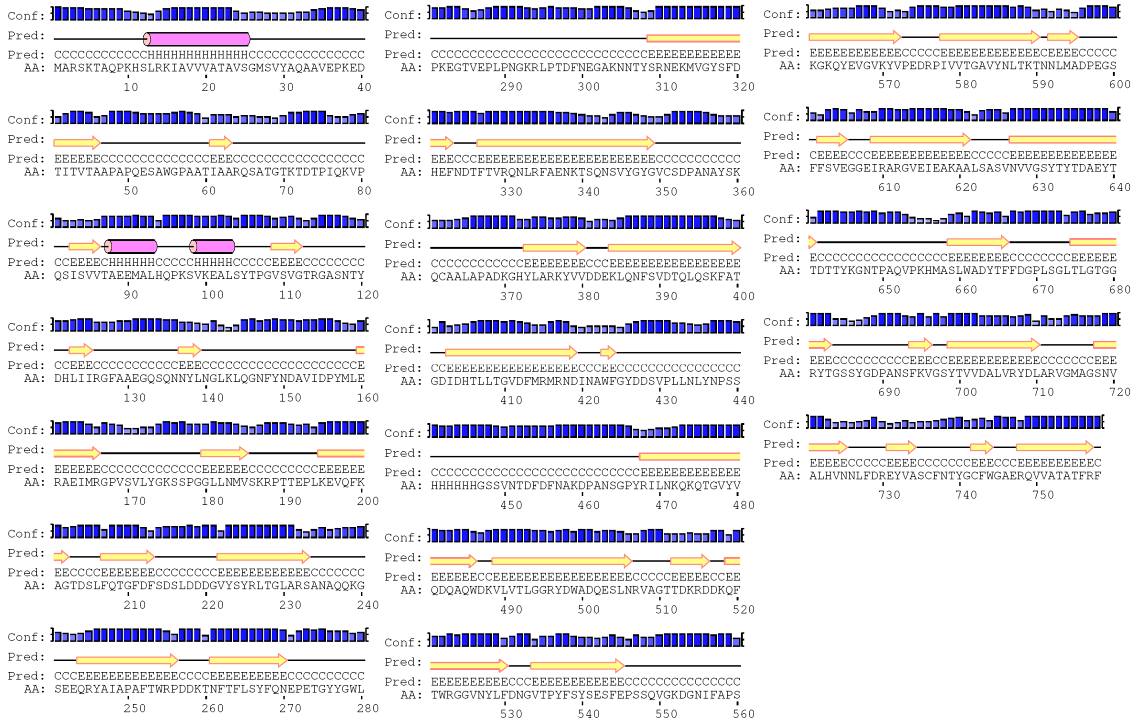

Tri-block copolymer structures of the PIBSA and PEG

Figure S4. Chemical structures of the polymer monomers s used in the experiments.

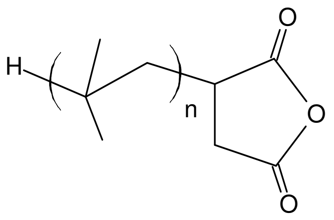

Polyisobutylene succinic anhydride (PIBSA)

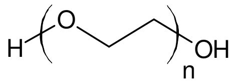

Polyethylene glycol (PEG)

Polymersomes DLS Data

Quasielastic light scattering with a laser particle sizer (Malvern Z-sizer Nano ZS, Malvern, UK) was used to determine the size distribution profile of the Polymersomes in solution. The laser pinhole (Helium-Neon gas, λ= 633 nm has a 100 μm diameter with a cell width of 1 cm (disposable PS cuvettes) was based on a sample volume of 750 μL. The scattering angle was set at 173° and determined at a running T = 25 °C, equilibrated for 120 sec. 3 measurements for each sample were conducted and for each single measurement 5 runs were performed. Each single run was accumulated for 120 sec. Solutions were measured without filtration and distributions calculated according to the CONTIN algorithm.

Figure S5. Size distribution of Polymersomes (PIB_{1000}-PEG_{6000}-PIB_{1000}) without inserted protein, prepared in phosphate buffer (0.1 M; pH 7.4) before size exclusion chromatography (see corresponding Section). Right plot correlation coefficient decay. (Maximum at 458 nm).

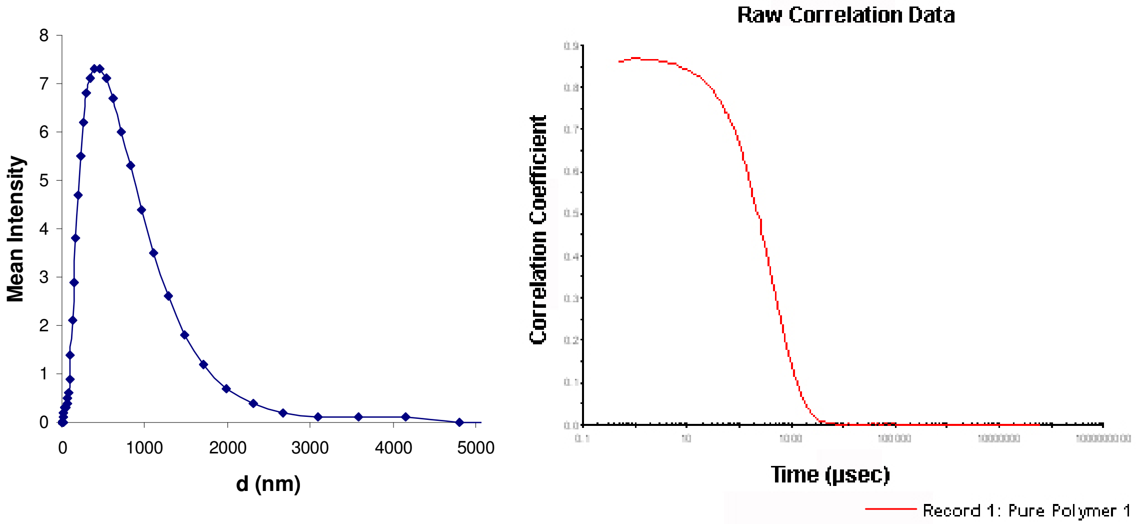

Figure S6. Size distribution of Polymerosomes (PIB_{1000}-PEG_{6000}-PIB_{1000}) without inserted protein, fraction 5-6 that were purified through size exclusion chromatography (see corresponding Section). Right plot correlation coefficient decay. (Maximum at 255 nm).

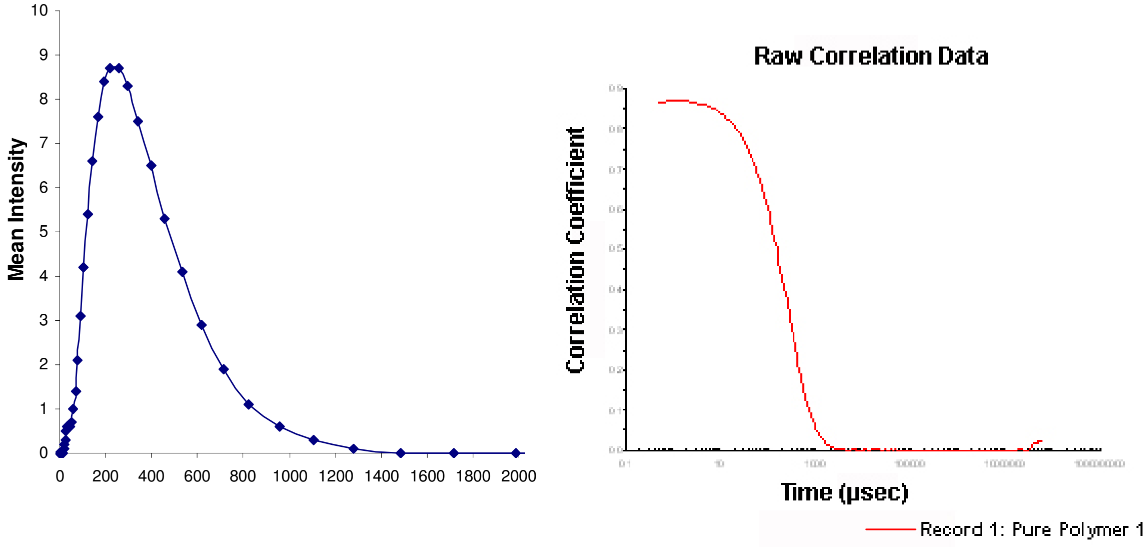

Figure S7. Size distribution of Polymerosomes (PIB_{1000}-PEG_{6000}-PIB_{1000}) without inserted protein, fraction 10 that were purified through size exclusion chromatography (see corresponding Section). Right plot correlation coefficient decay. (Maximum at 32 nm and 255 nm. It should be underlined that the intensity is proportional to r^6 so the contribution of the vesicles is predominant in the intensity but lower in population compared to the micelles.).

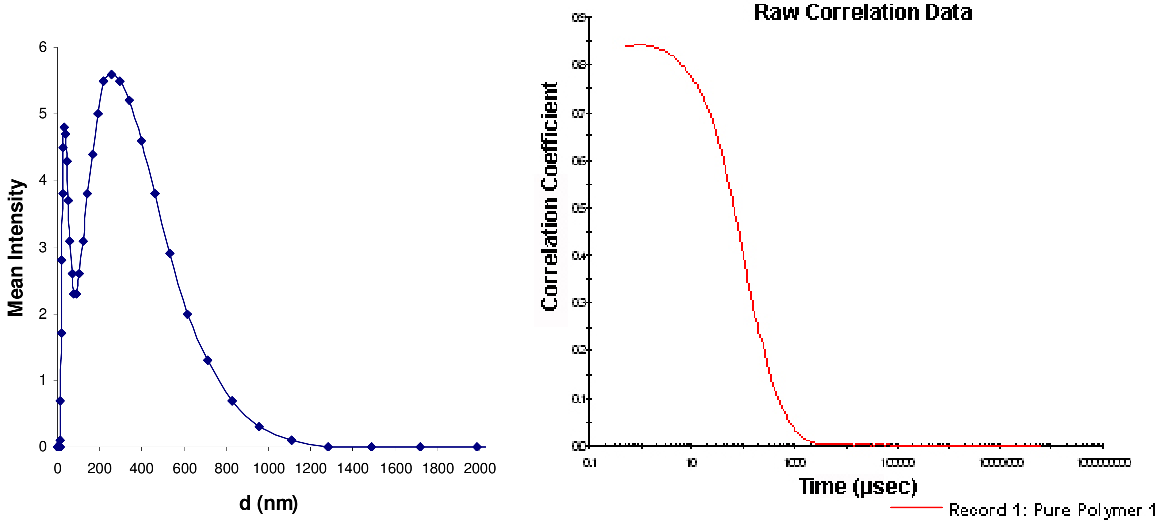

No vesicles or micelles were detected after the collected fraction 14**.**

Cryo TEM

Transmission Electron Microscopy (TEM) was carried out to check the polymersomes integrity and average radii. Fig. S8 shows a TEM image of the polymersome present in solution.

Figure S8. Cryo TEM pictures of the PIB_{1000}-PEG_{6000}-PIB_{1000} polymersomes. A diameter of ~400 nm is shown with a thick double layer ~40 nm (PIB_{1000} ~ n= 18 monomers, PEG_{6000} ~ n= 136 monomers) mainly expected to be PEG. Part 1 – TEM image with 3 µm scale. Part 2 - TEM images showing 2 polymer vesicles with 1 µm scale (A), zoom onto one of the polymersomes (B) and magnified part of the vesicle membrane (C).

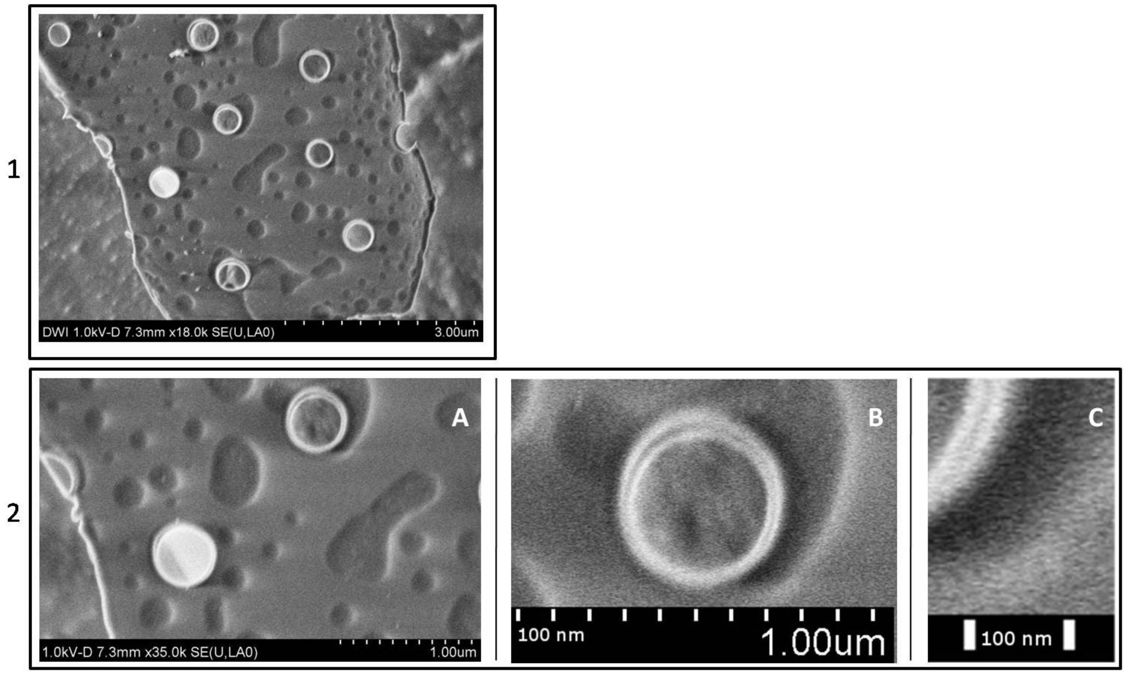

The cryo-TEM picture (Figure S8), shows a double wall morphology, where the PIB_{1000}-PEG_{6000}-PIB_{1000} (hydrophobic-hydrophilic-hydrphobic) is expected to relax in a "U" shape, with the PIB chains entangled in the middle as in a double bilayer. This can be further suggested by the bright white double layer of the polymersome cryo-TEM picture surely originated from the best electron scattering properties of the PEG block due to the presence of oxygen. The aforementioned picture suggests a double bilayer morphology of the polymersome wall. BAB-type copolymers have been reported to form vesicles with the copolymer chains looped into bilayer in majority (**Kurian P, Zschoche S, Kennedy JP. Synthesis and characterization of novel amphiphilic block copolymers di-, tri-, multi-, and star blocks of PEG and PIB.** ***J Polym Sci Pol Chem*** **2000, 38:3200-3209**

and

Yuan J, Li Y, Li X, Cheng S, Jiang L, Feng L, Fan Z: The ‘‘crew-cut’’ aggregates of polystyrene-b-poly(ethylene oxide)-b-polystyrene triblock copolymers in aqueous media. *Eur Polym J* 2003, 39:767–776.)

The thickness of the hydrophobic PIB layer cannot be deduced from the cryo-TEM due to the low resolution, however previous published data on PIB-PEO block copolymers with the PIB represented by ~18 monomers, a number exactly matching our system (**M. Rother, H. Barqawi, D. Pfefferkorn, J. Kressler, W. H. Binder (2010), Macromol.Chem.Phys. 211: 204-214**) report a 4.6 nm length. Furthermore, due to the PIB MW, such system is known to be an entangled bilayer (**G. C. Berry and T. G Fox (1968) Adv. Polym. Sci. 5: 261-357**), where chains are mutually interdigitated.

To complete the aforementioned picture, we have performed MD simulations calculating a membrane thickness of ~5.0 nm to show the morphology of the polymer (Figure S13) and its relative dimension compared to the FhuA Δ1-159 and the FhuA Δ1-159 Ext variant (Figure 1).

Finally, it should be underlined that the 40 nm membrane thickness represents the total thickness including the portion represented by the PEG6000, constituted by ~136 monomers.

HRP Assay

Figure S9. Wavelength scan of second TMB conversion product.

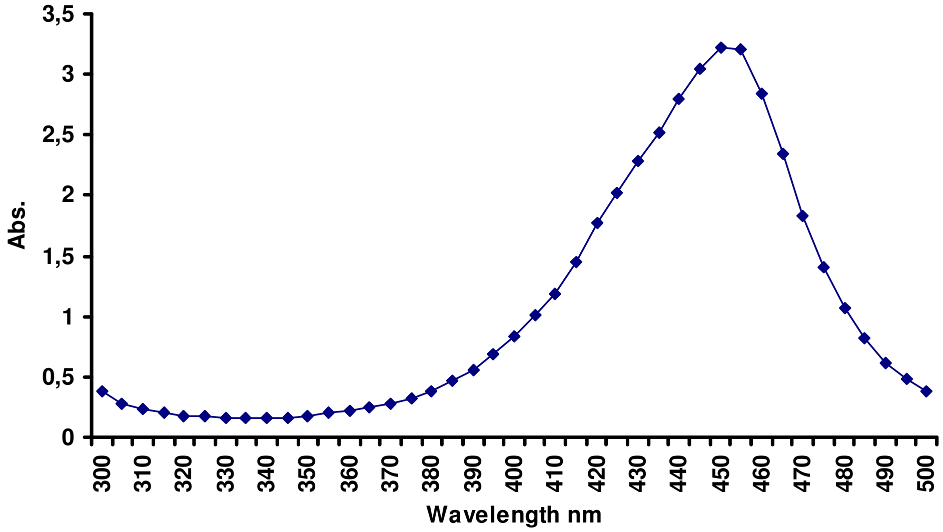

Figure S10. Time derivative of the absorbance was used to calculate the TMB conversion by the Lambert-Beer law. A linear regression using "least square" method was performed to find the best linear section in the steepest region for HRP loaded polymersome (triangles), HRP loaded polymersome + OES detergent (grey diamonds), HRP loaded polymersome + FhuA Δ1-159 (black minus), HRP loaded polymersome + unblocked FhuA Δ1-159 Ext (squares), HRP loaded polymersome + blocked FhuA Δ1-159 Ext (grey cycles) and Free HRP (black diamonds).

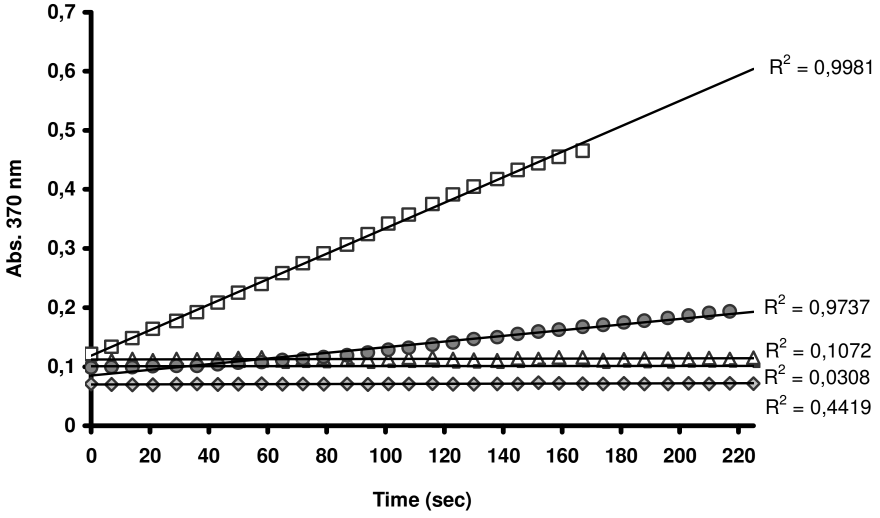

According to Lambert-Beer law, the empirical relationship that relates the absorption of light to the properties of the material through which the light is travelling:

$$\mathbf{A} = \boldsymbol{\varepsilon} \times \mathbf{c} \times \mathbf{L}$$

where A is the light absorbance, ε is the molar extinction coefficient of the substance, c is the molar concentration of the substance and L is the distance that the light travels through the material. The difference in absorbance over time is directly correlated to the difference in concentration of the TMB substrate. Therefore the slopes determined from the linear regions of the obtained kinetic graphs are a direct measure for the relative reaction speed (slopes: Table S1, ratio between blocked and unblocked: main text, "Influx kinetics and TMB/HRP detection system").

Because the extinction coefficient of the first TMB oxidation product at 370 nm is unknown, the actual conversion rate (nm/sec) could not be determined.

Table S1: Slopes of TMB/HRP assay kinetic data.

**TMB Conversion Speed**	
**Sample**	**TMB slopes (ΔA_{370}/sec)**
Polymers +HRP	6×10^{-5}
Polymers +HRP + OES	5×10^{-5}
Polymers + HRP + FhuA Δ1-159	2×10^{-5}
Polymers + HRP + FhuA Δ1-159 Ext	0.017
Polymers + HRP + FhuA Δ1-159 Ext (labelled)	0.0035

Consecutive Reaction Analysis

A two step irreversible consecutive reaction can be modeled by the chemical equation:

$$A \xrightarrow{k_1} B \xrightarrow{k_2} C$$

with rate constants k_1 and k_2.

The rate equations are:

$$(1) \qquad \begin{aligned} \frac{d[A]}{dt} &= -k_1[A] \\ \frac{d[B]}{dt} &= k_1[A] - k_2[B] \\ \frac{d[C]}{dt} &= k_2[B] \end{aligned}$$

Integration of the differential equations with $k_1 \neq k_2$ gives:

$$(2) \qquad \begin{aligned} [A] &= [A]_0 e^{-k_1 t} \\ [B] &= [A]_0 \frac{k_1}{k_2 - k_1}\left(e^{-k_1 t} - e^{-k_2 t}\right) + [B]_0 e^{-k_2 t} \\ [C] &= [A]_0 \left(1 + \frac{k_1 e^{-k_2 t} - k_2 e^{-k_1 t}}{k_2 - k_1}\right) + [B]_0 \left(1 - e^{-k_2 t}\right) + [C]_0 \end{aligned}$$

From literature (**Marquez LA, Dunford HB: Mechanism of the oxidation of 3,5,3',5'-tetramethylbenzidine by myeloperoxidase determined by transient and steady-state kinetics. *Biochemistry* 1997, 36:9349-9355**) the relation between the kinetic constants is $k_1 \approx > 5k_2$. Inserting a reference starting concentration of 1 [M], with $k_1 = 0,1$ and $k_2=0,02$ within system (2), the variation in time of A, B and C is shown in Fig. S11.

Figure S11. Calculated absorbances *vs.* time in an irreversible consecutive reaction $A \xrightarrow{k_1} B \xrightarrow{k_2} C$ measuring at three different non overlapping wavelengths. The turquoise B+C line reports the trend of the absorbance when $\varepsilon_{C370\ nm} = 0,1\ \varepsilon_{B370nm}$ (ε_B is unknown) reproducing the qualitative trend of the experimental data.

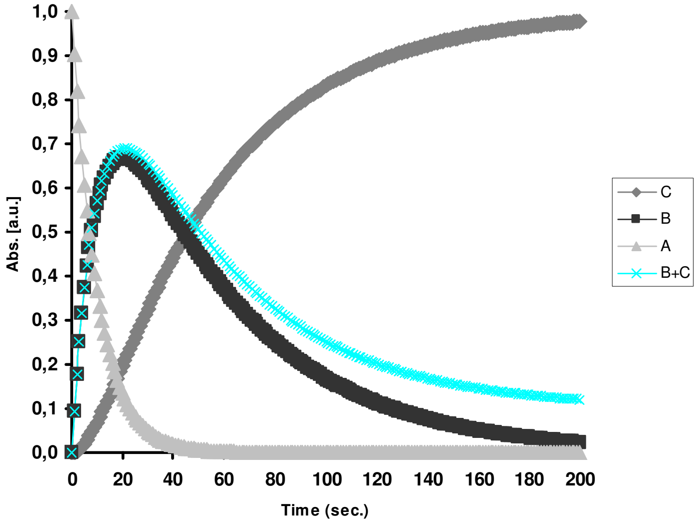

Because of the overlap in the adsorption band between the intermediate B and final product C (see Fig. S9), the measured adsorbance (370 nm) is related to the change of the total concentration of [B] + [C] and the reaction speed, calculated in the HRP assay is:

$$\frac{d\{[B]+[C]\}}{dt}$$

Guessing an absorbance relation of $C_{370\ nm} = 0,1\ B_{370\ nm}$ (ε_B and ε_C are unknown), as reported in legend Fig. S11 (turquoise points), we can reproduce the observed trend found in the absorbance experimental data (see Fig. 6).

Biotinylation Assay

Considering that FhuA Δ1-159 Ext contains a total of 29 Lys, the expected concentration of biotin used to label these residues can be calculated as follows:

1) Protein concentration:	**200 mg/ L**
2) Protein molecular weight:	**74600 g/ Mol**
3) Calculated molarity of FhuA Δ1-159 Ext:	**2.7 µM**
4) Calculated molar concentration of FhuA Δ1-159 Ext (100 µl of 1:2 diluted sample):	**135 pmol**
**5) Expected biotin concentration in case all 29 Lys are labelled:**	**3915 pmol**

Prior to the determination of biotin amount, the labelled, FhuA Δ1-159 Ext (free protein, not inserted into nanocontainers) was digested by proteases to reveal all biotin moieties. The relative fluorescence of the 1:100 diluted sample was 1495 corresponding to a concentration of ~3900 pmol (see calibration curve and related equation in Fig. S12).

Figure S12. Biotin-Assay calibration curve.

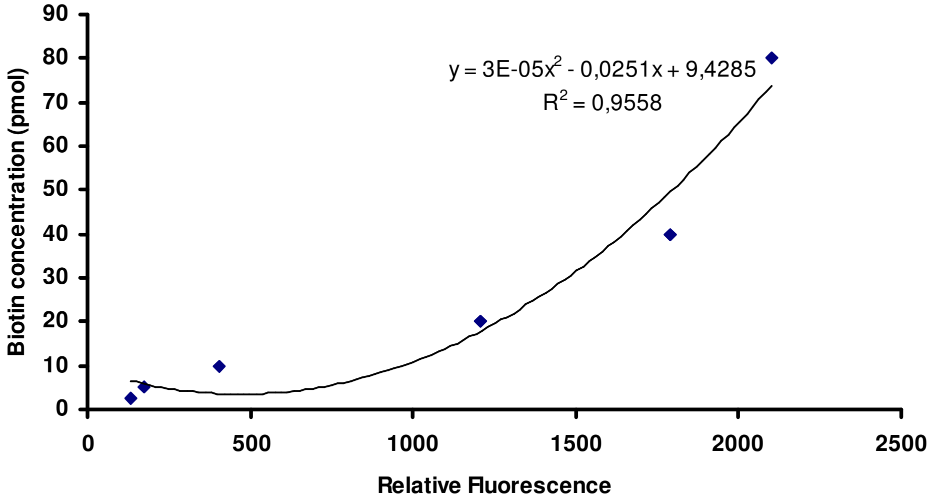

Molecular Dynamics PIB_{1000}-PEG_{1500}-PIB_{1000}

Atomistic Molecular Dynamics simulations on the triblock-copolymer PIB_{1000}-PEG_{1500}-PIB_{1000} have been performed to get first clues on the PIB hydrophobic thickness. To limit the number of atoms in the simulation box, PEG with a MW of 1500 (34 monomers) has been used instead of the MW=6000 (136 monomers), while maintaining the PIB MW=1000 (18 monomers). The introduced approximation is not expected to affect the hydrophobic layer, mainly governed by the entangled PIB chains. The used UA PEG Force Field has been derived from the model of Sadowski:

"**Modeling of Aqueous Poly(oxyethylene) Solutions: 1. Atomistic Simulations**"

Jan Fischer, Dietmar Paschek, Alfons Geiger, and Gabriele Sadowski

J. Phys. Chem. B, **2008**, 112 (8), 2388-2398• DOI: 10.1021/jp0765345

while the UA PIB FF has been derived from the model of Economu:

"Atomistic Simulation of the Sorption of Small Gas Molecules in Polyisobutylene"

Georgia Tsolou, Vlasis G. Mavrantzas, Zoi A. Makrodimitri and Ioannis G. Economou

Macromolecules **2008**, *41*, 6228-6238• DOI: 10.1021/ma8007652

All the simulations were performed using version GROMACS 4.0.7 molecular dynamics simulation package (www.gromacs.org). For all the simulated systems a cubic box employing periodic boundary conditions was utilized and a time step of 2 fs was used for the equations of motions numerical integration while atomic coordinates were saved every 5 ps. Simulations were conducted at a constant temperature of 340 K and a constant pressure of 1 bar. PIB_{1000}-PEG_{1500}-PIB_{1000} and water (SPC model) were independently coupled to a temperature bath, with a coupling constant of τ_T=0.1 ps by a V-rescale thermostat. A semi-isotropic pressure coupling was used, with a coupling constant of τ_P=1.0 ps and a compressibility of 4.5 10^{-5} bar^{-1} by a Berendsen barostat.

Energy minimizations were performed using a steepest descent algorithm followed by constrained molecular dynamics. Bond distances were constrained using the LINCS algorithm while the van der Waals interactions were modeled using a 6–12 Lennard-Jones potential with a cutoff at 1 nm. The electrostatic interactions were calculated by using the Particle Mesh Ewald algorithm (PME) with a cutoff of 1 nm for the direct space calculation. The reciprocal space calculation was performed using a fast Fourier transform algorithm.

System assembling

A double slab of 36+36 PIB_{1000}-PEG_{1500}-PIB_{1000} with the PIB chains partially entangled (50 % of the PIB extended length) was built by using a grid of 6*6 points equally distributed. The center of mass of each single chain was placed in one of the grid points, with a random rotational orientation referenced to the polymer chains latitudinal main axis. In fact each single polymer chain can be well figured by a "U" shape, where the upper terminal sides are the two PIB chains linked by a PEG chain. Distances between chains were minimized to overlap the PIB VdW sphere to reduce the presence of spurious water during solvation procedure. The final box configuration of 72 PIB_{1000}-PEG_{1500}-PIB_{1000} molecules and 16108 SPC water resulted in linear dimensions of: 9.34*9.34*10.95 nm. 30 ns of relaxation at 340 K to maintain the PIB fluid were followed by 50 ns of production run. The final box configuration with marked examples of three PIB and two PEG chains is shown in Fig. S13.

To understand if the PIB_{1000}-PEG_{1500}-PIB_{1000} chains are sufficiently relaxed to give a reliable representation of the system near equilibrium, the autocorrelation function time decay of the chain end-to-end unit vector, defined as $\tau_D = \langle \mathbf{u}(t) \cdot \mathbf{u}(0) \rangle$ has been calculated (T=340 K, τ_D=25 ns). Although longer simulation times would be necessary for a better PES exploration, with a time decay of 25 ns and a simulation run of 50 ns after 30 ns of equilibration, the system can be considered equilibrated.

Figure S13. 50 ns final box configuration showing 2 PEG chains in their characteristic "U" conformation (VdW representation, upper chains with red oxygen atoms) and the embedded PIB chains in their extended and globular conformations (VdW, lower chains). The hydrophobic PIB thickness is ~5 nm.

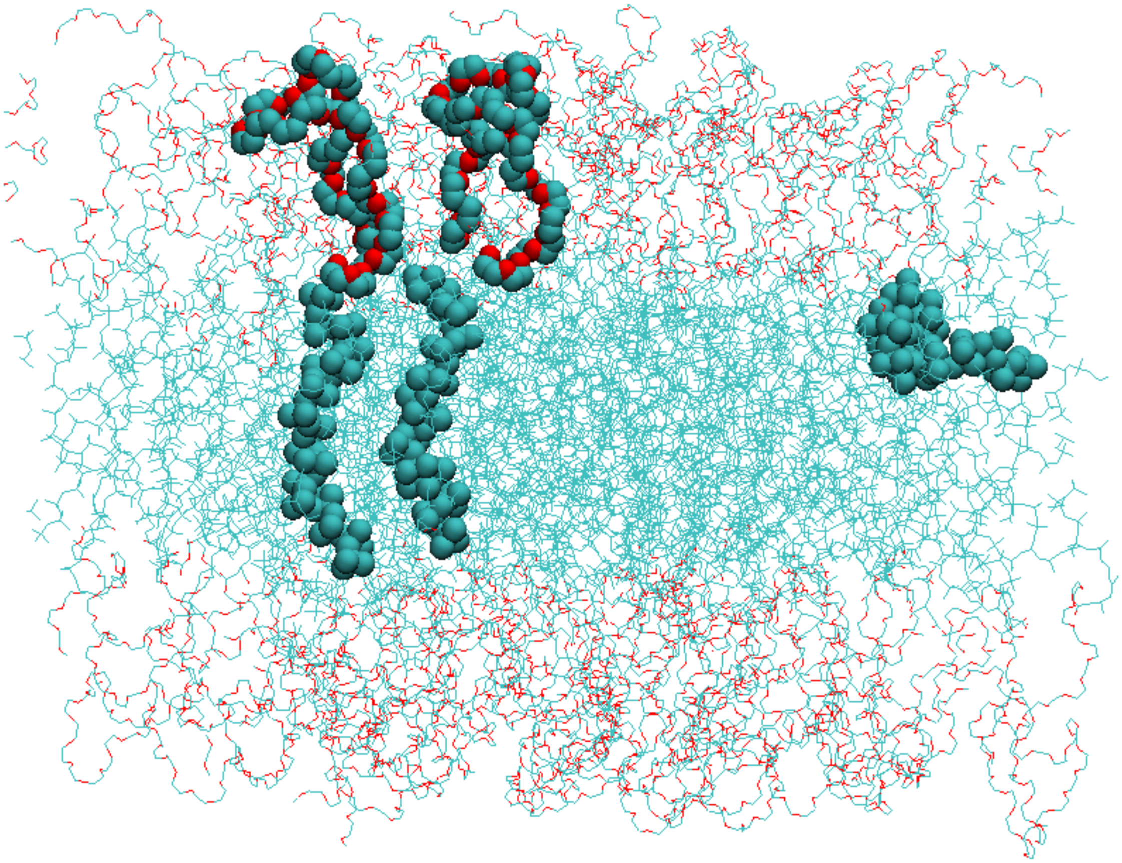

CD Results

Figure S14. CD spectrum of FhuA Δ1-159 Ext in 1mM potassium phosphate buffer containing 0.5% OES (grey squares) and plot of data fit carried out with CONTIN algorithm using the program Dichroprot (black crosses).

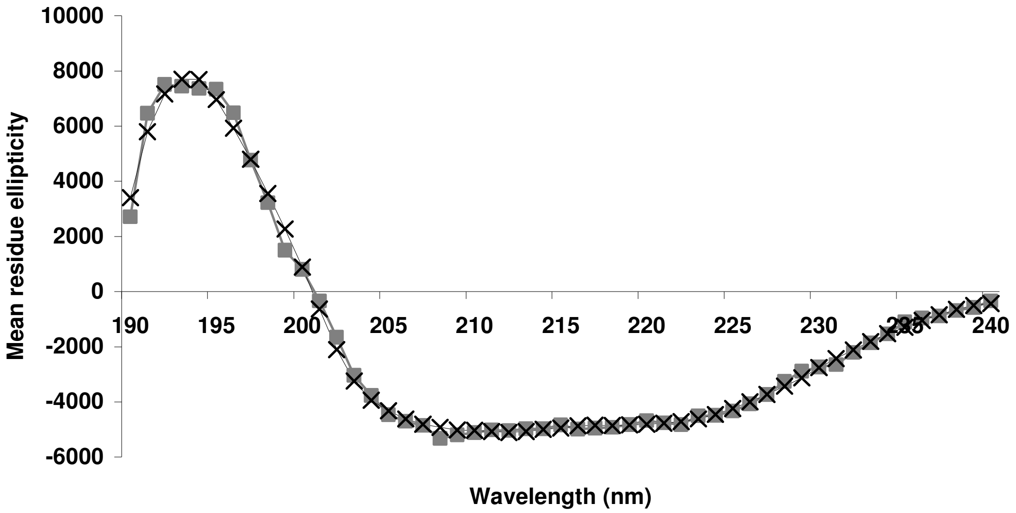

Fitting error:

	HELIX	BETA-SHEET	REMAINDER	SCALE FACTOR
FRACTION	0.20	0.75	0.05	0.980
STANDARD ERROR	3.0E-02	4.2E-02	5.7E-02	

Figure S15. CD spectrum of biotinylated FhuA Δ1-159 Ext in 1mM potassium phosphate buffer containing 0.5% OES (grey squares) and plot of data fit carried out with CONTIN algorithm using the program Dichroprot (black crosses).

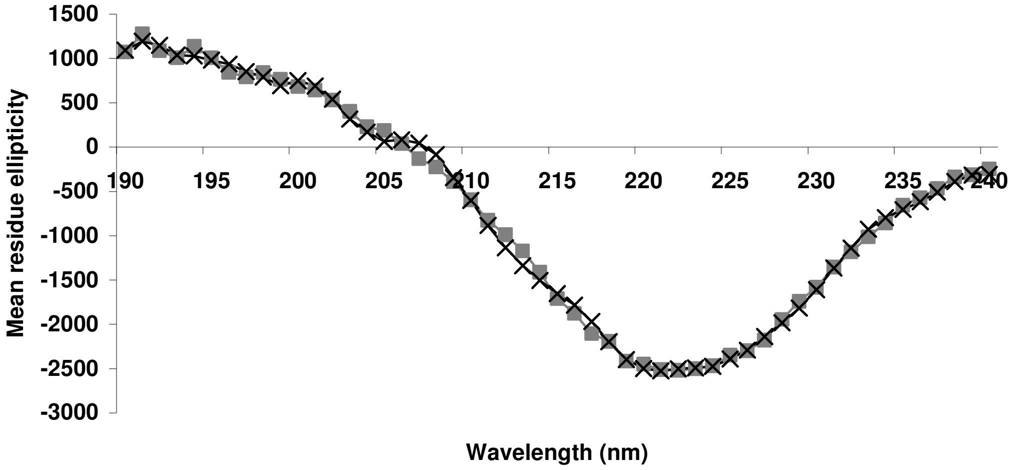

Fitting error:

	HELIX	BETA-SHEET	REMAINDER	SCALE FACTOR
FRACTION	0.00	0.58	0.42	0.913
STANDARD ERROR	3.2E-02	2.6E-02	1.5E-02	

Figure S16. CD spectrum of FhuA Δ1-159 Ext reconstituted into PIB_{1000}-PEG_{6000}-PIB_{1000} membranes (grey squares). The polymersome fraction after gel filtration has been concentrated ~ 10-fold. Fitting of the data was carried out with CONTIN algorithm using the program Dichroprot (black crosses).

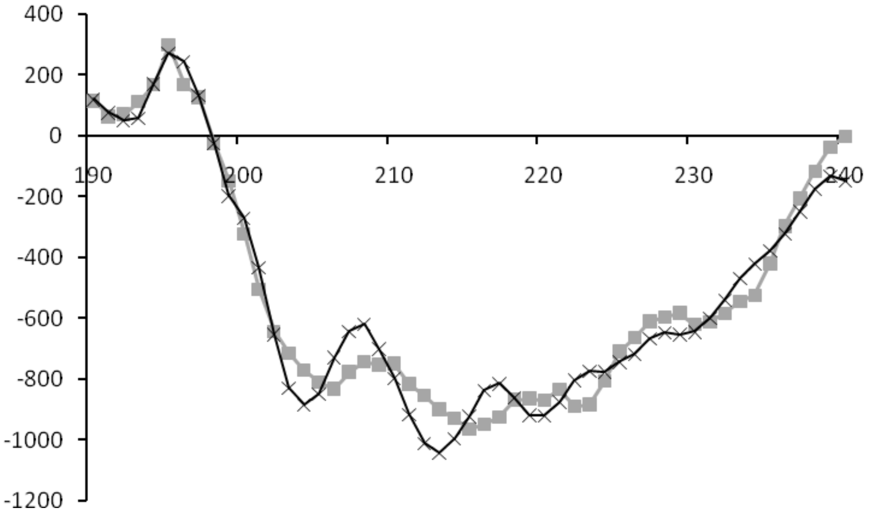

Fitting error:

	HELIX	BETA-SHEET	REMAINDER	SCALE FACTOR
FRACTION	0.00	0.65	0.35	0.912
STANDARD ERROR	9.0E-09	2.7E-02	1.6E-02	
