# Supplementary material for: Engineering of the E. coli Outer Membrane Protein FhuA to overcome the Hydrophobic Mismatch in Thick Polymeric Membranes
Source: J Nanobiotechnology. 2011 Mar 17;9:8. doi: 10.1186/1477-3155-9-8 (PMC3064644; doi:10.1186/1477-3155-9-8)
Supplement: Additional file 2 — Deconvolution analysis of FhuA Δ1-159 Ext (unlabelled) in octyl-pOE (detergent). CD spectra deconvolution analysis by the CONTIN algorithm of the FhuA Δ1-159 Ext (unlabelled) in octyl-pOE (detergent) solution. [file 1477-3155-9-8-S2.PDF]

```
 CONTIN - VERSION 2DP (MAR 1984) ( CD-1  PACK)       TEST DATA SET 1 - FOR CD PACKAGE


 REFERENCES - S.W. PROVENCHER (1982) COMPUT. PHYS. COMMUN., VOL. 27, PAGES 213-227, 229-242.
                             (1984) EMBL TECHNICAL REPORT DA07 (EUROPEAN MOLECULAR BIOLOGY LABORATORY,
HEIDELBERG, F.R. OF GERMANY)


                   INPUT DATA FOR CHANGES TO COMMON VARIABLES

 IFORMY    0    0.00000E+00
 (7F9.0)

 LAST      0    1.00000E+00

 IWT       0    5.00000E+00

 IUSER    14    3.10000E+01

 IUSER    15   -1.00000E+00

 RUSER    14    1.00000E+00

 RUSER    15    3.00000E-02

 RUSER    16    5.00000E+02

 END       0    0.00000E+00
 NSTEND   51    2.40000E+02    1.90000E+02



                                        FINAL VALUES OF CONTROL VARIABLES
 DFMIN  = 3.00000E+00
 SRMIN  = 1.00000E-02
 ALPST  = 0.00000E+00 0.00000E+00
 GMNMX  = 1.00000E+00 1.60000E+01
 PLEVEL = 5.00000E-01 5.00000E-01 5.00000E-01 5.00000E-01
 RSVMNX = 1.00000E+00 1.00000E+00 0.00000E+00 0.00000E+00
 RUSER  = 0.00000E+00 0.00000E+00 0.00000E+00 0.00000E+00 0.00000E+00 0.00000E+00 0.00000E+00 0.00000E+00 0.00000E+00
0.00000E+00 0.00000E+00
          0.00000E+00 0.00000E+00 0.00000E+00 1.00000E+00 3.00000E-02 5.00000E+02 0.00000E+00 0.00000E+00
0.00000E+00 0.00000E+00
          0.00000E+00 0.00000E+00 0.00000E+00 0.00000E+00 0.00000E+00 0.00000E+00 0.00000E+00 0.00000E+00
0.00000E+00 0.00000E+00
          0.00000E+00 0.00000E+00 0.00000E+00 0.00000E+00 0.00000E+00 0.00000E+00 0.00000E+00 0.00000E+00
0.00000E+00 0.00000E+00
```

```
          0.00000E+00 0.00000E+00 0.00000E+00 0.00000E+00 0.00000E+00 0.00000E+00 0.00000E+00 0.00000E+00
0.00000E+00 0.00000E+00
          0.00000E+00 0.00000E+00 0.00000E+00 0.00000E+00 0.00000E+00 0.00000E+00 0.00000E+00 0.00000E+00
0.00000E+00 0.00000E+00
          0.00000E+00 0.00000E+00 0.00000E+00 0.00000E+00 0.00000E+00 0.00000E+00 0.00000E+00 0.00000E+00
0.00000E+00 0.00000E+00
          0.00000E+00 0.00000E+00 0.00000E+00 0.00000E+00 0.00000E+00 0.00000E+00 0.00000E+00 0.00000E+00
0.00000E+00 0.00000E+00
          0.00000E+00 0.00000E+00 0.00000E+00 0.00000E+00 0.00000E+00 0.00000E+00 0.00000E+00 0.00000E+00
0.00000E+00 0.00000E+00
          0.00000E+00 0.00000E+00 0.00000E+00 0.00000E+00 0.00000E+00 0.00000E+00 0.00000E+00 0.00000E+00
0.00000E+00 0.00000E+00
 IGRID  =          1
 IQUAD  =          1
 IUNIT  =         -1
 IWT    =          5
 LINEPG =         60
 MIOERR =          5
 MPKMOM =          0
 MQPITR =         35
 NEQ    =          0
 NERFIT =          0
 NG     =         16
 NINTT  =          1
 NLINF  =          0
 NORDER =         -1
 ICRIT  =          1           1
 IFORMT = (5E15.6)
 IFORMW = (5E15.6)
 IFORMY = (7F9.0)
 IPLFIT =          2           2
 IPLRES =          2           2
 IPRINT =          2           3
 IUSER  =          0           0           0           0           0           0           0           0
  0           0
                   0           0           0          31          -1           4           7           0
  0           0
                   0           0           0           0           0           0           0           0
  0           0
                   0           0           0           0           0           0           0           0
  0           0
                   0           0           0           0           0           0           0           0
  0           0
 IUSROU =          3           3
 LSIGN  =          0           0           0           0           0           0           0           0
  0           0
                   0           0           0           0           0           0
```

```
 MOMNMX =            0           0
 NENDZ  =            0           0
 NFLAT  =            0           0           0           0           0           0           0           0
 NNSGN  =            0           0
 NQPROG =            6           6
 NSGN   =            0           0           0           0
 DOCHOS =            T
 DOMOM  =            F
 DOUSIN =            T
 DOUSNQ =            T
 LAST   =            T
 NEWPG1 =            F
 NONNEG =            F
 ONLY1  =            T
 PRWT   =            T
 PRY    =            T
 SIMULA =            F
 LUSER  =            F           F           F           F           F           F           F           F
  F           F
                     F           F           F           F           F           F           F           F
  F           F
                     F           F           F           F           F           F           F           F
  F           F



           T            Y            T            Y            T            Y            T            Y
  T            Y
    2.400E+02 -3.41000E+02    2.390E+02 -5.76000E+02    2.380E+02 -6.92000E+02    2.370E+02 -8.87000E+02
2.360E+02 -9.65000E+02
    2.350E+02 -1.10500E+03    2.340E+02 -1.54200E+03    2.330E+02 -1.86800E+03    2.320E+02 -2.21300E+03
2.310E+02 -2.64900E+03
    2.300E+02 -2.73900E+03    2.290E+02 -2.89200E+03    2.280E+02 -3.25400E+03    2.270E+02 -3.73600E+03
2.260E+02 -4.07300E+03
    2.250E+02 -4.33800E+03    2.240E+02 -4.48600E+03    2.230E+02 -4.51000E+03    2.220E+02 -4.82500E+03
2.210E+02 -4.75800E+03
    2.200E+02 -4.68400E+03    2.190E+02 -4.82200E+03    2.180E+02 -4.92500E+03    2.170E+02 -4.95100E+03
2.160E+02 -4.98500E+03
    2.150E+02 -4.83200E+03    2.140E+02 -4.97700E+03    2.130E+02 -4.97200E+03    2.120E+02 -5.04400E+03
2.110E+02 -5.01600E+03
    2.100E+02 -5.12300E+03    2.090E+02 -5.19600E+03    2.080E+02 -5.32500E+03    2.070E+02 -4.85300E+03
2.060E+02 -4.70600E+03
    2.050E+02 -4.46300E+03    2.040E+02 -3.76900E+03    2.030E+02 -3.04000E+03    2.020E+02 -1.66100E+03
2.010E+02 -3.38000E+02
    2.000E+02  7.98000E+02    1.990E+02  1.49900E+03    1.980E+02  3.21500E+03    1.970E+02  4.76700E+03
1.960E+02  6.48100E+03
    1.950E+02  7.33500E+03    1.940E+02  7.36600E+03    1.930E+02  7.44200E+03    1.920E+02  7.51100E+03
```

```
1.910E+02  6.45900E+03
    1.900E+02  2.70500E+03    0.000E+00  1.00000E+00
0PRECIS = 1.49E-15           SRANGE = 1.00E+35     RANGE = 1.00E+35


   GRID POINT     MIN IN MATRIX A    AT T =     MAX IN MATRIX A    AT T =     SCALE FACTOR
   1.0000E+00         -2.4876E+04  2.22E+02          5.5079E+04  1.92E+02        2.078E-06
   2.0000E+00         -1.3387E+04  2.08E+02          1.7434E+04  1.92E+02        2.078E-06
   3.0000E+00         -1.1109E+04  2.11E+02          1.6667E+04  0.00E+00        2.078E-06
   4.0000E+00         -1.2593E+04  2.09E+02          1.6667E+04  0.00E+00        2.078E-06
   5.0000E+00         -1.3227E+04  2.22E+02          2.4142E+04  1.93E+02        2.078E-06
   6.0000E+00         -9.7762E+03  2.03E+02          1.6667E+04  0.00E+00        2.078E-06
   7.0000E+00         -6.6914E+03  2.24E+02          1.6667E+04  0.00E+00        2.078E-06
   8.0000E+00         -1.2254E+04  2.23E+02          1.6667E+04  0.00E+00        2.078E-06
   9.0000E+00         -1.2473E+04  1.99E+02          1.6667E+04  0.00E+00        2.078E-06
   1.0000E+01         -1.1208E+04  2.09E+02          1.6667E+04  0.00E+00        2.078E-06
   1.1000E+01         -1.3714E+04  2.09E+02          2.5943E+04  1.96E+02        2.078E-06
   1.2000E+01         -1.4514E+04  2.22E+02          1.9732E+04  1.95E+02        2.078E-06
   1.3000E+01         -9.9252E+03  2.12E+02          1.6667E+04  0.00E+00        2.078E-06
   1.4000E+01         -1.8628E+04  2.03E+02          1.6667E+04  0.00E+00        2.078E-06
   1.5000E+01         -1.4012E+04  2.10E+02          2.8231E+04  1.92E+02        2.078E-06
   1.6000E+01         -1.1083E+04  2.11E+02          1.6667E+04  0.00E+00        2.078E-06
0SCALE FACTOR FOR ALPHA =  7.700E+06

   0 UNREGULARIZED VARIABLES

 SINGULAR VALUES
     4.036E-02    1.461E-02    6.268E-03    3.163E-03    1.902E-03    1.817E-03    1.383E-03    5.122E-04
4.872E-04    3.099E-04
     2.796E-04    1.190E-04    8.184E-05    6.539E-05    2.937E-05    2.580E-05
1
           TEST DATA SET 1 - FOR CD PACKAGE                                                     PRELIMINARY
UNWEIGHTED ANALYSIS

      ALPHA    ALPHA/S(1)     OBJ. FCTN.       VARIANCE      STD. DEV.    DEG FREEDOM    PROB1 TO REJECT    PROB2
TO REJECT
 * 6.01E-17      1.49E-15    1.51902E+06    1.51902E+06      2.026E+02        15.000              0.000
    1.000

                     HELIX   BETA-SHEET    REMAINDER                 SCALE FACTOR
 FRACTION             0.25        0.75         0.00                       1.001
 STANDARD ERROR      2.5E-02      2.7E-02      2.7E-08
0(FOR ALPHA/S(1) = 1.49E-15) PRUNS = 0.4445         PUNCOR =  0.9449  0.0088  0.0394  0.8624  0.5110


           TEST DATA SET 1 - FOR CD PACKAGE                                                     PRELIMINARY
```

```
UNWEIGHTED ANALYSIS

      ALPHA    ALPHA/S(1)     OBJ. FCTN.       VARIANCE      STD. DEV.    DEG FREEDOM    PROB1 TO REJECT    PROB2
TO REJECT
 * 4.41E-14      1.09E-12    1.51902E+06    1.51902E+06      2.026E+02         15.000              0.000
   1.000

                       HELIX   BETA-SHEET    REMAINDER                SCALE FACTOR
 FRACTION               0.25         0.75         0.00                       1.001
 STANDARD ERROR      2.5E-02      2.7E-02      2.7E-08
0(FOR ALPHA/S(1) = 1.09E-12) PRUNS = 0.4445         PUNCOR =  0.9449  0.0088  0.0394  0.8624  0.5110


           TEST DATA SET 1 - FOR CD PACKAGE                                                         PRELIMINARY
UNWEIGHTED ANALYSIS

      ALPHA    ALPHA/S(1)     OBJ. FCTN.       VARIANCE      STD. DEV.    DEG FREEDOM    PROB1 TO REJECT    PROB2
TO REJECT
 * 3.23E-11      8.02E-10    1.51902E+06    1.51902E+06      2.026E+02         15.000              0.000
   1.000

                       HELIX   BETA-SHEET    REMAINDER                SCALE FACTOR
 FRACTION               0.25         0.75         0.00                       1.001
 STANDARD ERROR      2.5E-02      2.7E-02      2.7E-08
0(FOR ALPHA/S(1) = 8.02E-10) PRUNS = 0.4445         PUNCOR =  0.9449  0.0088  0.0394  0.8624  0.5110


           TEST DATA SET 1 - FOR CD PACKAGE                                                         PRELIMINARY
UNWEIGHTED ANALYSIS

      ALPHA    ALPHA/S(1)     OBJ. FCTN.       VARIANCE      STD. DEV.    DEG FREEDOM    PROB1 TO REJECT    PROB2
TO REJECT
 * 2.37E-08      5.88E-07    1.51902E+06    1.51902E+06      2.026E+02         15.000              0.000
   1.000

                       HELIX   BETA-SHEET    REMAINDER                SCALE FACTOR
 FRACTION               0.25         0.75         0.00                       1.001
 STANDARD ERROR      2.5E-02      2.7E-02      2.2E-08
0(FOR ALPHA/S(1) = 5.88E-07) PRUNS = 0.4445         PUNCOR =  0.9449  0.0088  0.0394  0.8624  0.5110


           TEST DATA SET 1 - FOR CD PACKAGE                                                         PRELIMINARY
UNWEIGHTED ANALYSIS
```

```
      ALPHA    ALPHA/S(1)     OBJ. FCTN.       VARIANCE      STD. DEV.    DEG FREEDOM    PROB1 TO REJECT    PROB2
TO REJECT
   1.74E-05      4.31E-04    1.98350E+06    1.59968E+06      2.064E+02         14.448              0.000
    0.916

                       HELIX   BETA-SHEET    REMAINDER                SCALE FACTOR
 FRACTION               0.24         0.76         0.00                       1.001
 STANDARD ERROR      2.0E-02      2.3E-02      1.6E-08
0(FOR ALPHA/S(1) = 4.31E-04) PRUNS = 0.2418         PUNCOR =  0.9452  0.0030  0.0180  0.5928  0.3183


           TEST DATA SET 1 - FOR CD PACKAGE                                                      PRELIMINARY
UNWEIGHTED ANALYSIS

      ALPHA    ALPHA/S(1)     OBJ. FCTN.       VARIANCE      STD. DEV.    DEG FREEDOM    PROB1 TO REJECT    PROB2
TO REJECT
   1.28E-02      3.16E-01    1.88342E+08    9.03419E+07      1.341E+03          1.786              1.000
    1.000

                       HELIX   BETA-SHEET    REMAINDER                SCALE FACTOR
 FRACTION               0.29         0.27         0.43                       0.649
 STANDARD ERROR      9.7E-03      8.3E-03      1.3E-02
0(FOR ALPHA/S(1) = 3.16E-01) PRUNS =-1.0000         PUNCOR =  0.4806  0.0965  0.0298  0.1626  0.4389


           TEST DATA SET 1 - FOR CD PACKAGE                                                      PRELIMINARY
UNWEIGHTED ANALYSIS

      ALPHA    ALPHA/S(1)     OBJ. FCTN.       VARIANCE      STD. DEV.    DEG FREEDOM    PROB1 TO REJECT    PROB2
TO REJECT
   4.47E-05      1.11E-03    3.22574E+06    2.26387E+06      2.416E+02         13.215              0.692
    1.000

                       HELIX   BETA-SHEET    REMAINDER                SCALE FACTOR
 FRACTION               0.23         0.77         0.00                       0.999
 STANDARD ERROR      1.5E-02      1.9E-02      8.1E-09
0(FOR ALPHA/S(1) = 1.11E-03) PRUNS = 0.0626         PUNCOR =  0.6401  0.0020  0.0047  0.7540  0.3079


           TEST DATA SET 1 - FOR CD PACKAGE                                                      PRELIMINARY
UNWEIGHTED ANALYSIS

      ALPHA    ALPHA/S(1)     OBJ. FCTN.       VARIANCE      STD. DEV.    DEG FREEDOM    PROB1 TO REJECT    PROB2
TO REJECT
```

```
   1.15E-04      2.84E-03    6.01769E+06    4.01260E+06      3.161E+02         11.832              1.000
    1.000

                       HELIX   BETA-SHEET    REMAINDER                SCALE FACTOR
 FRACTION               0.19         0.71         0.10                       0.992
 STANDARD ERROR       1.9E-02      2.8E-02      3.4E-02
0(FOR ALPHA/S(1) = 2.84E-03) PRUNS = 0.0048          PUNCOR =  0.4185  0.0113  0.0116  0.7416  0.8755


           TEST DATA SET 1 - FOR CD PACKAGE                                                       PRELIMINARY
UNWEIGHTED ANALYSIS

      ALPHA    ALPHA/S(1)     OBJ. FCTN.       VARIANCE      STD. DEV.    DEG FREEDOM    PROB1 TO REJECT    PROB2
TO REJECT
   2.94E-04      7.29E-03    1.16078E+07    7.30225E+06      4.153E+02          9.659              1.000
    1.000

                       HELIX   BETA-SHEET    REMAINDER                SCALE FACTOR
 FRACTION               0.12         0.61         0.26                       0.977
 STANDARD ERROR       1.2E-02      2.8E-02      2.4E-02
0(FOR ALPHA/S(1) = 7.29E-03) PRUNS = 0.0039          PUNCOR =  0.5401  0.1651  0.1982  0.3949  0.0942


           TEST DATA SET 1 - FOR CD PACKAGE                                                       PRELIMINARY
UNWEIGHTED ANALYSIS

      ALPHA    ALPHA/S(1)     OBJ. FCTN.       VARIANCE      STD. DEV.    DEG FREEDOM    PROB1 TO REJECT    PROB2
TO REJECT
   7.55E-04      1.87E-02    2.42532E+07    1.40301E+07      5.605E+02          7.336              1.000
    1.000

                       HELIX   BETA-SHEET    REMAINDER                SCALE FACTOR
 FRACTION               0.11         0.52         0.36                       0.927
 STANDARD ERROR       1.0E-02      2.7E-02      2.0E-02
0(FOR ALPHA/S(1) = 1.87E-02) PRUNS = 0.0044          PUNCOR =  0.9362  0.8485  0.7325  0.1968  0.0086


           TEST DATA SET 1 - FOR CD PACKAGE                                                       PRELIMINARY
UNWEIGHTED ANALYSIS

      ALPHA    ALPHA/S(1)     OBJ. FCTN.       VARIANCE      STD. DEV.    DEG FREEDOM    PROB1 TO REJECT    PROB2
TO REJECT
   1.94E-03      4.80E-02    5.30688E+07    3.40371E+07      8.518E+02          5.094              1.000
    1.000
```

```
                       HELIX   BETA-SHEET    REMAINDER                SCALE FACTOR
 FRACTION               0.18         0.41         0.41                       0.781
 STANDARD ERROR      1.1E-02      2.5E-02      2.4E-02
0(FOR ALPHA/S(1) = 4.80E-02) PRUNS =-1.0000         PUNCOR =  0.3716  0.1369  0.0738  0.4813  0.6472



           TEST DATA SET 1 - FOR CD PACKAGE                                                     PRELIMINARY
UNWEIGHTED ANALYSIS

      ALPHA    ALPHA/S(1)     OBJ. FCTN.       VARIANCE      STD. DEV.    DEG FREEDOM    PROB1 TO REJECT    PROB2
TO REJECT
   4.97E-03      1.23E-01    9.13090E+07    6.64682E+07      1.166E+03          3.128              1.000
    1.000

                       HELIX   BETA-SHEET    REMAINDER                SCALE FACTOR
 FRACTION               0.26         0.31         0.43                       0.652
 STANDARD ERROR      1.1E-02      1.6E-02      2.2E-02
0(FOR ALPHA/S(1) = 1.23E-01) PRUNS =-1.0000         PUNCOR =  0.2793  0.0589  0.0205  0.1906  0.7022
1CONTIN 2DP (MAR 84) ( CD-1 )    TEST DATA SET 1 - FOR CD PACKAGE
  CHOSEN SOLUTION

 WEIGHTED RESIDUALS (ALPHA/S(1)= 1.11E-03) MAX=U= 5.9E+02  MIN=L=-5.7E+02 (PRUNS= 0.0626) PUNCOR= 0.6401 0.0020
0.0047 0.7540 0.3079

 U---------+---------+---------+---------+---------*--

                                              *
                                               *
             *                                    *
       *      *                          **
  *   *            *  *    * ***      *
        *      *       *    *      *   *             *
 0-***---*--*---*-*--*----------*---*-----*--*--------
          *      *  *   ***      *   *
                                            *
           *                      *              *
                                                *


 L---------+---------+---------+---------+-*-------+*-
                     20                  40                  60                  80                 100
      120
```

```
0PLOT OF DATA (O) AND FIT TO DATA (X).  ORDINATES LISTED ARE FIT VALUES.

    ORDINATE  ABSCISSA
  -4.902E+02  2.40E+02                                        XO

  -5.373E+02  2.39E+02                                       OX

  -6.723E+02  2.38E+02                                      *

  -8.670E+02  2.37E+02                                     *

  -1.080E+03  2.36E+02                                   XO

  -1.320E+03  2.35E+02                                 X O

  -1.562E+03  2.34E+02                               *

  -1.817E+03  2.33E+02                            OX

  -2.093E+03  2.32E+02                          OX

  -2.402E+03  2.31E+02                      O X

  -2.742E+03  2.30E+02                     *

  -3.137E+03  2.29E+02                  X O

  -3.485E+03  2.28E+02               X O

  -3.776E+03  2.27E+02            XO

  -4.028E+03  2.26E+02          *

  -4.260E+03  2.25E+02        *

  -4.466E+03  2.24E+02       *

  -4.614E+03  2.23E+02     XO

  -4.717E+03  2.22E+02    OX

  -4.763E+03  2.21E+02    *

  -4.794E+03  2.20E+02    XO

  -4.833E+03  2.19E+02    *
```

```
-4.855E+03  2.18E+02   *
-4.830E+03  2.17E+02   OX
-4.864E+03  2.16E+02  OX
-4.947E+03  2.15E+02   XO
-5.004E+03  2.14E+02  *
-5.109E+03  2.13E+02 XO
-5.156E+03  2.12E+02 XO
-5.119E+03  2.11E+02 XO
-5.077E+03  2.10E+02 OX
-5.101E+03  2.09E+02 *
-5.027E+03  2.08E+02O X
-4.930E+03  2.07E+02   *
-4.702E+03  2.06E+02     *
-4.348E+03  2.05E+02       OX
-3.900E+03  2.04E+02           X O
-3.057E+03  2.03E+02                  XO
-1.903E+03  2.02E+02                            X O
-5.471E+02  2.01E+02                                       X O
 8.066E+02  2.00E+02                                                   *
 2.072E+03  1.99E+02                                                         O   X
 3.377E+03  1.98E+02                                                                       OX
 4.823E+03  1.97E+02                                                                                    *
 6.021E+03  1.96E+02
X   O
 7.006E+03  1.95E+02
```

```
            X O
  7.716E+03  1.94E+02
                 O X
  7.713E+03  1.93E+02
                 O X
  7.252E+03  1.92E+02
               X O
  5.865E+03  1.91E+02
X    O
  3.272E+03  1.90E+02                                                                                  O   X

  9.993E-01  0.00E+00                                                             *



RMS RESIDUAL FOR PTS. 1 TO 31 =    1.15E+02
RMS RESIDUAL FOR REMAINING PTS. = 3.04E+02


ERRFIT = 0.00E+00
                                 SQUARE ROOTS OF LEAST SQUARES WEIGHTS
    8.6696E-03   8.6696E-03   8.6696E-03   8.6696E-03   8.6696E-03   8.6696E-03   8.6696E-03   8.6696E-03
8.6696E-03   8.6696E-03
    8.6696E-03   8.6696E-03   8.6696E-03   8.6696E-03   8.6696E-03   8.6696E-03   8.6696E-03   8.6696E-03
8.6696E-03   8.6696E-03
    8.6696E-03   8.6696E-03   8.6696E-03   8.6696E-03   8.6696E-03   8.6696E-03   8.6696E-03   8.6696E-03
8.6696E-03   8.6696E-03
    8.6696E-03   3.2868E-03   3.2868E-03   3.2868E-03   3.2868E-03   3.2868E-03   3.2868E-03   3.2868E-03
3.2868E-03   3.2868E-03
    3.2868E-03   3.2868E-03   3.2868E-03   3.2868E-03   3.2868E-03   3.2868E-03   3.2868E-03   3.2868E-03
3.2868E-03   3.2868E-03
    3.2868E-03   3.3333E+01


  GRID POINT     MIN IN MATRIX A    AT T =     MAX IN MATRIX A    AT T =     SCALE FACTOR
  1.0000E+00        -2.1567E+02  2.22E+02          1.8103E+02  1.92E+02        3.492E-04
  2.0000E+00        -1.1162E+02  2.10E+02          5.7301E+01  1.92E+02        3.492E-04
  3.0000E+00        -9.6307E+01  2.11E+02          3.3333E+01  0.00E+00        3.492E-04
  4.0000E+00        -1.0913E+02  2.10E+02          4.8647E+01  1.90E+02        3.492E-04
  5.0000E+00        -1.1467E+02  2.22E+02          7.9350E+01  1.93E+02        3.492E-04
  6.0000E+00        -6.3028E+01  2.10E+02          3.3333E+01  0.00E+00        3.492E-04
  7.0000E+00        -5.8012E+01  2.24E+02          3.9426E+01  1.97E+02        3.492E-04
  8.0000E+00        -1.0624E+02  2.23E+02          4.2599E+01  1.96E+02        3.492E-04
  9.0000E+00        -6.2318E+01  2.10E+02          3.3333E+01  0.00E+00        3.492E-04
  1.0000E+01        -9.6014E+01  2.10E+02          4.0725E+01  1.90E+02        3.492E-04
  1.1000E+01        -1.1770E+02  2.10E+02          8.5270E+01  1.96E+02        3.492E-04
  1.2000E+01        -1.2583E+02  2.22E+02          6.4854E+01  1.95E+02        3.492E-04
```

```
   1.3000E+01         -8.6048E+01  2.12E+02          4.8245E+01  1.98E+02        3.492E-04
   1.4000E+01         -1.0890E+02  2.10E+02          3.3333E+01  0.00E+00        3.492E-04
   1.5000E+01         -1.2148E+02  2.10E+02          9.2790E+01  1.92E+02        3.492E-04
   1.6000E+01         -9.6089E+01  2.11E+02          3.3333E+01  0.00E+00        3.492E-04
0SCALE FACTOR FOR ALPHA =  4.582E+04

   0 UNREGULARIZED VARIABLES

 SINGULAR VALUES
     4.220E-02    8.995E-03    3.608E-03    3.141E-03    2.003E-03    1.378E-03    7.621E-04    5.513E-04
4.278E-04    2.556E-04
     1.990E-04    1.206E-04    7.136E-05    3.986E-05    3.302E-05    2.289E-05
1
           TEST DATA SET 1 - FOR CD PACKAGE

      ALPHA    ALPHA/S(1)     OBJ. FCTN.       VARIANCE      STD. DEV.    DEG FREEDOM    PROB1 TO REJECT    PROB2
TO REJECT
 * 6.29E-17      1.49E-15    4.56319E+01    4.56319E+01      1.111E+00        15.000              0.000
    1.000

    ORDINATE    ERROR  ABSCISSA
   9.753E-01  2.8E-01  1.00E+00
.......X.......
  -5.210E-01  3.2E-01  2.00E+00                                            ........X........

   1.070E+00  6.9E-01  3.00E+00
.................X.................
  -3.729E-01  1.1E-01  4.00E+00                                                     ..X...

  -8.781E-01  5.7E-01  5.00E+00                    ...............X.............

   6.127E-01  1.8E-01  6.00E+00                                                                     .....X....

   8.176E-01  1.8E-01  7.00E+00                                                                           ....X....

   7.773E-02  1.6E-01  8.00E+00                                                        .....X....

   1.717E+00  2.5E-01  9.00E+00
        ......X
  -2.285E+00  2.9E-01  1.00E+01X.......

  -4.621E-01  9.8E-02  1.10E+01                                                   ..X..

   8.567E-01  1.9E-01  1.20E+01
.....X.....
   6.390E-02  1.6E-01  1.30E+01                                                        ....X....
```

```
   9.932E-02  1.4E-01  1.40E+01                                                              ...X...

   3.741E-01  2.1E-01  1.50E+01                                                                   .....X.....

  -1.143E+00  7.0E-01  1.60E+01           .................X.................


                       HELIX    BETA-SHEET    REMAINDER               SCALE FACTOR
 FRACTION               0.23        0.77         0.00                        1.002
 STANDARD ERROR      2.7E-02     4.0E-02      2.5E-08
0(FOR ALPHA/S(1) = 1.49E-15) PRUNS = 0.2418         PUNCOR =  0.1471  0.0195  0.0003  0.1051  0.7689


           TEST DATA SET 1 - FOR CD PACKAGE

      ALPHA    ALPHA/S(1)     OBJ. FCTN.       VARIANCE      STD. DEV.    DEG FREEDOM    PROB1 TO REJECT    PROB2
TO REJECT
 * 3.66E-14      8.68E-13    4.56319E+01    4.56319E+01      1.111E+00         15.000              0.000
    1.000

    ORDINATE    ERROR  ABSCISSA
   9.753E-01  2.8E-01  1.00E+00
.......X.......
  -5.210E-01  3.2E-01  2.00E+00                                                ........X........

   1.070E+00  6.9E-01  3.00E+00
.................X.................
  -3.729E-01  1.1E-01  4.00E+00                                                         ..X...

  -8.781E-01  5.7E-01  5.00E+00                    ...............X..............

   6.127E-01  1.8E-01  6.00E+00                                                                       .....X....

   8.176E-01  1.8E-01  7.00E+00                                                                             ....X....

   7.773E-02  1.6E-01  8.00E+00                                                              .....X....

   1.717E+00  2.5E-01  9.00E+00
          ......X
  -2.285E+00  2.9E-01  1.00E+01X.......

  -4.621E-01  9.8E-02  1.10E+01                                                       ..X..

   8.567E-01  1.9E-01  1.20E+01
.....X.....
   6.390E-02  1.6E-01  1.30E+01                                                              ....X....
```

```
   9.932E-02  1.4E-01  1.40E+01                                                        ...X...

   3.741E-01  2.1E-01  1.50E+01                                                            .....X.....

  -1.143E+00  7.0E-01  1.60E+01          .................X.................


                       HELIX   BETA-SHEET    REMAINDER              SCALE FACTOR
 FRACTION               0.23         0.77         0.00                     1.002
 STANDARD ERROR      2.7E-02      4.0E-02      2.5E-08
0(FOR ALPHA/S(1) = 8.68E-13) PRUNS = 0.2418         PUNCOR =  0.1471  0.0195  0.0003  0.1051  0.7689


           TEST DATA SET 1 - FOR CD PACKAGE

      ALPHA    ALPHA/S(1)     OBJ. FCTN.       VARIANCE      STD. DEV.    DEG FREEDOM    PROB1 TO REJECT    PROB2
TO REJECT
 * 2.13E-11      5.06E-10    4.56319E+01    4.56319E+01      1.111E+00         15.000              0.000
    1.000

    ORDINATE    ERROR  ABSCISSA
   9.753E-01  2.8E-01  1.00E+00
.......X.......
  -5.210E-01  3.2E-01  2.00E+00                                   ........X........

   1.070E+00  6.9E-01  3.00E+00
.................X.................
  -3.729E-01  1.1E-01  4.00E+00                                            ..X...

  -8.781E-01  5.7E-01  5.00E+00                  ...............X..............

   6.127E-01  1.8E-01  6.00E+00                                                              .....X....

   8.176E-01  1.8E-01  7.00E+00                                                                    ....X....

   7.773E-02  1.6E-01  8.00E+00                                                       .....X....

   1.717E+00  2.5E-01  9.00E+00
          ......X
  -2.285E+00  2.9E-01  1.00E+01X.......

  -4.621E-01  9.8E-02  1.10E+01                                         ..X..

   8.567E-01  1.9E-01  1.20E+01
.....X.....
```

```
   6.390E-02  1.6E-01  1.30E+01                                                          ....X....

   9.932E-02  1.4E-01  1.40E+01                                                            ...X...

   3.741E-01  2.1E-01  1.50E+01                                                                .....X.....

  -1.143E+00  7.0E-01  1.60E+01           .................X.................


                     HELIX   BETA-SHEET    REMAINDER               SCALE FACTOR
 FRACTION             0.23         0.77         0.00                      1.002
 STANDARD ERROR      2.7E-02      4.0E-02      2.5E-08
0(FOR ALPHA/S(1) = 5.06E-10) PRUNS = 0.2418         PUNCOR =  0.1471  0.0195  0.0003  0.1051  0.7689


           TEST DATA SET 1 - FOR CD PACKAGE

     ALPHA    ALPHA/S(1)     OBJ. FCTN.       VARIANCE      STD. DEV.    DEG FREEDOM    PROB1 TO REJECT    PROB2
TO REJECT
 * 1.24E-08      2.95E-07    4.56319E+01    4.56319E+01      1.111E+00        15.000              0.000
    1.000

    ORDINATE    ERROR  ABSCISSA
   9.753E-01  2.8E-01  1.00E+00
.......X.......
  -5.210E-01  3.2E-01  2.00E+00                                      ........X........

   1.070E+00  6.9E-01  3.00E+00
.................X................
  -3.729E-01  1.1E-01  4.00E+00                                               ..X...

  -8.781E-01  5.7E-01  5.00E+00                    ...............X..............

   6.127E-01  1.8E-01  6.00E+00                                                           .....X....

   8.176E-01  1.8E-01  7.00E+00                                                                 ....X....

   7.773E-02  1.6E-01  8.00E+00                                                    .....X....

   1.717E+00  2.5E-01  9.00E+00
          .....X
  -2.285E+00  2.9E-01  1.00E+01X.......

  -4.621E-01  9.8E-02  1.10E+01                                             ..X..

   8.567E-01  1.9E-01  1.20E+01
```

```
.....X.....
   6.390E-02  1.6E-01  1.30E+01                                                        ....X....

   9.932E-02  1.4E-01  1.40E+01                                                          ...X...

   3.741E-01  2.1E-01  1.50E+01                                                               .....X.....

  -1.143E+00  7.0E-01  1.60E+01           .................X..................


                       HELIX   BETA-SHEET    REMAINDER                SCALE FACTOR
 FRACTION               0.23         0.77         0.00                       1.002
 STANDARD ERROR      2.7E-02      4.0E-02      9.4E-09
O(FOR ALPHA/S(1) = 2.95E-07) PRUNS = 0.2418         PUNCOR =  0.1471  0.0195  0.0003  0.1051  0.7689


           TEST DATA SET 1 - FOR CD PACKAGE

      ALPHA    ALPHA/S(1)     OBJ. FCTN.       VARIANCE      STD. DEV.    DEG FREEDOM    PROB1 TO REJECT    PROB2
TO REJECT
   7.24E-06      1.72E-04    4.71866E+01    4.57060E+01      1.109E+00         14.846              0.000
    0.791

    ORDINATE    ERROR  ABSCISSA
   9.306E-01  2.7E-01  1.00E+00
......X.......
  -4.649E-01  3.0E-01  2.00E+00                                              ........X.......

   9.484E-01  6.3E-01  3.00E+00
................X................
  -3.553E-01  1.0E-01  4.00E+00                                                      ..X...

  -8.178E-01  5.4E-01  5.00E+00                                 ..............X..............

   5.804E-01  1.7E-01  6.00E+00                                                                  ....X.....

   8.049E-01  1.7E-01  7.00E+00
....X.....
   6.990E-02  1.6E-01  8.00E+00                                                        ....X....

   1.672E+00  2.4E-01  9.00E+00
          ......X
  -2.223E+00  2.7E-01  1.00E+01X......

  -4.441E-01  9.4E-02  1.10E+01                                                    ..X...
```

```
   8.210E-01  1.8E-01  1.20E+01
.....X....
   3.594E-02  1.5E-01  1.30E+01                                                      ...X....

   8.448E-02  1.3E-01  1.40E+01                                                       ....X...

   3.562E-01  2.1E-01  1.50E+01                                                            ......X.....

  -9.979E-01  6.4E-01  1.60E+01               ................X...............


                       HELIX   BETA-SHEET    REMAINDER                SCALE FACTOR
 FRACTION               0.23         0.77         0.00                       1.001
 STANDARD ERROR      2.6E-02      3.9E-02      2.4E-08
0(FOR ALPHA/S(1) = 1.72E-04) PRUNS = 0.2418         PUNCOR =  0.1526  0.0180  0.0002  0.1112  0.7458



           TEST DATA SET 1 - FOR CD PACKAGE

      ALPHA    ALPHA/S(1)     OBJ. FCTN.       VARIANCE      STD. DEV.    DEG FREEDOM    PROB1 TO REJECT    PROB2
TO REJECT
   4.22E-03      1.00E-01    1.20777E+03    5.81998E+02      3.444E+00          2.935              1.000
    1.000

    ORDINATE     ERROR  ABSCISSA
   1.208E-02  5.3E-03  1.00E+00..........X...........

   3.647E-02  3.3E-03  2.00E+00                                                       .......X.......

   4.076E-02  4.8E-03  3.00E+00                                                             ..........X.........

   7.331E-03  5.2E-03  4.00E+00X...........

   3.061E-02  2.0E-03  5.00E+00                                             ....X....

   4.342E-02  3.8E-03  6.00E+00
........X.........
   5.422E-02  5.4E-03  7.00E+00
     ...........X
   2.164E-02  4.2E-03  8.00E+00                     .........X.........

   4.930E-02  3.4E-03  9.00E+00
.......X.......
   2.932E-02  1.7E-03  1.00E+01                                           ...X....

   4.374E-02  4.5E-03  1.10E+01
```

```
..........X..........
   1.741E-02  4.7E-03  1.20E+01           ..........X..........

   3.194E-02  5.8E-03  1.30E+01                                 ............X............

   3.096E-02  4.5E-03  1.40E+01                                 ..........X.........

   3.428E-02  2.6E-03  1.50E+01                                            ......X......

   4.014E-02  4.9E-03  1.60E+01                                                    ..........X...........


                       HELIX   BETA-SHEET    REMAINDER                SCALE FACTOR
 FRACTION               0.30        0.29         0.41                       0.524
 STANDARD ERROR      6.3E-03     1.0E-02      1.4E-02
0(FOR ALPHA/S(1) = 1.00E-01) PRUNS = 0.0003         PUNCOR =  0.8567  0.0280  0.0246  0.1868  0.6400



           TEST DATA SET 1 - FOR CD PACKAGE

      ALPHA    ALPHA/S(1)     OBJ. FCTN.       VARIANCE      STD. DEV.    DEG FREEDOM    PROB1 TO REJECT    PROB2
TO REJECT
   1.80E-05      4.26E-04    5.35065E+01    4.71294E+01      1.118E+00        14.283              0.000
    0.806

    ORDINATE    ERROR  ABSCISSA
   7.556E-01  2.2E-01  1.00E+00
......X.......
  -2.779E-01  2.5E-01  2.00E+00                                   .......X.......

   5.716E-01  4.4E-01  3.00E+00
............X............
  -2.965E-01  8.8E-02  4.00E+00                                       ..X...

  -5.653E-01  4.4E-01  5.00E+00                     .............X............

   4.725E-01  1.4E-01  6.00E+00                                                       ....X....

   7.472E-01  1.4E-01  7.00E+00
....X....
   4.425E-02  1.5E-01  8.00E+00                                            ....X....

   1.500E+00  2.1E-01  9.00E+00
           .....X
  -2.006E+00  2.2E-01  1.00E+01X......
```

```
  -3.804E-01  8.3E-02  1.10E+01                                          ..X..

   6.977E-01  1.5E-01  1.20E+01                                                                   .....X....

  -6.826E-02  1.3E-01  1.30E+01                                                 ....X...

   3.081E-02  1.3E-01  1.40E+01                                                    ....X...

   3.002E-01  1.9E-01  1.50E+01                                                          .....X......

  -5.293E-01  4.4E-01  1.60E+01                               .............X...........


                       HELIX   BETA-SHEET    REMAINDER                SCALE FACTOR
 FRACTION               0.22         0.78         0.00                       0.996
 STANDARD ERROR      2.2E-02      3.6E-02      1.3E-08
0(FOR ALPHA/S(1) = 4.26E-04) PRUNS = 0.1660          PUNCOR =  0.1599  0.0162  0.0003  0.1187  0.7272


           TEST DATA SET 1 - FOR CD PACKAGE

      ALPHA    ALPHA/S(1)     OBJ. FCTN.       VARIANCE      STD. DEV.    DEG FREEDOM    PROB1 TO REJECT    PROB2
TO REJECT
   4.47E-05      1.06E-03    7.45893E+01    5.75478E+01      1.223E+00         13.502              0.181
    0.992

    ORDINATE    ERROR  ABSCISSA
   3.422E-01  1.3E-01  1.00E+00                                                                .....X.....

   6.103E-02  1.9E-01  2.00E+00                                                 .......X........

   1.595E-01  1.8E-01  3.00E+00                                                     .......X........

  -1.756E-01  7.3E-02  4.00E+00                                            ...X...

  -4.889E-02  2.4E-01  5.00E+00                                          ..........X..........

   2.720E-01  1.1E-01  6.00E+00                                                            .....X....

   5.999E-01  8.7E-02  7.00E+00
...X....
  -6.827E-02  1.3E-01  8.00E+00                                              .....X.....

   1.074E+00  1.5E-01  9.00E+00
          ......X
  -1.389E+00  2.5E-01  1.00E+01X..........
```

```
 -2.302E-01  7.4E-02  1.10E+01                                          ...X...

  4.706E-01  1.1E-01  1.20E+01                                                                   ....X....

 -2.810E-01  8.2E-02  1.30E+01                                       ...X....

 -1.161E-01  1.3E-01  1.40E+01                                            .....X.....

  2.335E-01  1.7E-01  1.50E+01                                                         ......X.......

  7.533E-02  1.8E-01  1.60E+01                                                  .......X.......

                     HELIX   BETA-SHEET    REMAINDER                SCALE FACTOR
 FRACTION             0.20         0.75         0.05                       0.980
 STANDARD ERROR      3.0E-02      4.2E-02      5.7E-02
0(FOR ALPHA/S(1) = 1.06E-03) PRUNS = 0.0191         PUNCOR =  0.1674  0.0284  0.0008  0.1131  0.9623


           TEST DATA SET 1 - FOR CD PACKAGE

     ALPHA    ALPHA/S(1)     OBJ. FCTN.       VARIANCE      STD. DEV.    DEG FREEDOM    PROB1 TO REJECT    PROB2
TO REJECT
   1.11E-04      2.63E-03    1.16769E+02    8.40998E+01     1.442E+00         11.541              0.965
    1.000

    ORDINATE    ERROR  ABSCISSA
   2.370E-02  5.5E-02  1.00E+00                                      ......X.....

   2.999E-01  1.0E-01  2.00E+00                                                      .........X.........

   1.610E-01  5.5E-02  3.00E+00                                                 .....X.....

  -7.477E-02  5.9E-02  4.00E+00                             .....X......

   8.446E-02  7.3E-02  5.00E+00                                          .......X.......

   1.076E-01  7.2E-02  6.00E+00                                             ......X.......

   5.001E-01  5.1E-02  7.00E+00
  .....X.....
  -2.398E-01  8.3E-02  8.00E+00             ........X........

   6.028E-01  9.5E-02  9.00E+00
       ........X
```

```
 -4.698E-01  9.3E-02  1.00E+01X........

 -3.374E-02  6.2E-02  1.10E+01                                  ......X......

  2.365E-01  6.8E-02  1.20E+01                                                         ......X.......

 -3.519E-01  5.5E-02  1.30E+01     .....X......

 -2.255E-01  8.4E-02  1.40E+01              ........X........

  1.947E-01  1.0E-01  1.50E+01                                                  .........X..........

  1.378E-01  5.3E-02  1.60E+01                                                 .....X.....


                      HELIX   BETA-SHEET    REMAINDER                SCALE FACTOR
 FRACTION              0.13         0.65         0.22                       0.953
 STANDARD ERROR      1.7E-02      3.7E-02      3.4E-02
0(FOR ALPHA/S(1) = 2.63E-03) PRUNS = 0.0191         PUNCOR =  0.4324  0.0824  0.0222  0.1986  0.5196



          TEST DATA SET 1 - FOR CD PACKAGE

      ALPHA    ALPHA/S(1)     OBJ. FCTN.       VARIANCE      STD. DEV.    DEG FREEDOM    PROB1 TO REJECT    PROB2
TO REJECT
   2.76E-04      6.53E-03    2.04449E+02    1.38995E+02      1.806E+00          9.391              1.000
    1.000

    ORDINATE    ERROR  ABSCISSA
 -2.676E-02  2.5E-02  1.00E+00                        ....X.....

  1.993E-01  4.2E-02  2.00E+00                                                         .......X.......

  1.117E-01  2.3E-02  3.00E+00                                              ....X...

 -1.015E-01  4.1E-02  4.00E+00        .......X.......

  9.879E-02  2.7E-02  5.00E+00                                           ....X.....

  1.085E-01  5.0E-02  6.00E+00                                         ........X........

  3.988E-01  3.7E-02  7.00E+00
      ......X
 -1.740E-01  5.1E-02  8.00E+00....X........

  3.109E-01  5.0E-02  9.00E+00
```

```
........X........
  -1.081E-01  3.3E-02  1.00E+01         ......X.....

   8.896E-02  4.8E-02  1.10E+01                                        ........X........

   2.757E-02  4.1E-02  1.20E+01                               ......X.......

  -1.994E-01  3.5E-02  1.30E+01X.....

  -6.537E-02  4.1E-02  1.40E+01               .......X.......

   1.273E-01  5.2E-02  1.50E+01                                             .........X.........

   8.371E-02  2.4E-02  1.60E+01                                           ....X....

                       HELIX   BETA-SHEET    REMAINDER                SCALE FACTOR
 FRACTION               0.12         0.56         0.31                       0.880
 STANDARD ERROR      1.3E-02      3.9E-02      2.9E-02
0(FOR ALPHA/S(1) = 6.53E-03) PRUNS = 0.0042         PUNCOR =  0.9615  0.5162  0.3574  0.1748  0.0654


           TEST DATA SET 1 - FOR CD PACKAGE

      ALPHA    ALPHA/S(1)     OBJ. FCTN.       VARIANCE      STD. DEV.    DEG FREEDOM    PROB1 TO REJECT    PROB2
TO REJECT
   6.84E-04      1.62E-02    3.66917E+02    2.53044E+02      2.374E+00          7.088              1.000
    1.000

    ORDINATE     ERROR  ABSCISSA
  -3.524E-04  1.2E-02  1.00E+00                          ...X....

   9.223E-02  1.7E-02  2.00E+00                                                 .....X......

   6.746E-02  1.4E-02  3.00E+00                                           ....X....

  -1.048E-01  2.6E-02  4.00E+00X.......

   7.052E-02  1.4E-02  5.00E+00                                            ....X....

   1.026E-01  2.8E-02  6.00E+00                                                 .........X........

   2.351E-01  2.8E-02  7.00E+00
        ........X
  -6.306E-02  2.3E-02  8.00E+00     .......X.......
```

```
   1.454E-01  2.2E-02  9.00E+00                                                                      ......X.......

   1.197E-02  1.1E-02  1.00E+01                              ...X...

   9.450E-02  2.8E-02  1.10E+01                                                     ........X........

  -2.957E-02  2.5E-02  1.20E+01                 ........X.......

  -6.421E-02  2.4E-02  1.30E+01     .......X........

   3.520E-02  2.0E-02  1.40E+01                                  ......X......

   6.733E-02  1.9E-02  1.50E+01                                            .....X......

   5.483E-02  1.4E-02  1.60E+01                                         ....X.....

                       HELIX   BETA-SHEET    REMAINDER                SCALE FACTOR
  FRACTION              0.17         0.46         0.36                       0.715
  STANDARD ERROR     1.2E-02      3.6E-02      2.8E-02
 0(FOR ALPHA/S(1) = 1.62E-02) PRUNS = 0.0805         PUNCOR =  0.2172  0.7228  0.5908  0.7461  0.2864


           TEST DATA SET 1 - FOR CD PACKAGE

     ALPHA    ALPHA/S(1)     OBJ. FCTN.       VARIANCE      STD. DEV.    DEG FREEDOM    PROB1 TO REJECT    PROB2
 TO REJECT
   1.70E-03      4.03E-02    6.11050E+02    4.42337E+02      3.065E+00          4.900              1.000
    1.000

    ORDINATE     ERROR  ABSCISSA
   1.510E-02  9.2E-03  1.00E+00                                .......X.......

   4.960E-02  9.2E-03  2.00E+00                                                        ........X.......

   4.978E-02  1.1E-02  3.00E+00                                                        ........X........

  -3.641E-02  1.3E-02  4.00E+00X..........

   3.849E-02  5.5E-03  5.00E+00                                                   ....X....

   5.735E-02  1.1E-02  6.00E+00
 .........X.........
   9.163E-02  1.4E-02  7.00E+00
       ..........X
  -3.076E-03  1.0E-02  8.00E+00                     ........X........
```

```
   6.875E-02  7.5E-03  9.00E+00
......X.....
   2.667E-02  4.0E-03  1.00E+01                                              ...X...

   6.425E-02  1.3E-02  1.10E+01
..........X..........
   1.324E-03  1.3E-02  1.20E+01                   ..........X..........

   3.802E-03  1.6E-02  1.30E+01                   ............X...........

   3.874E-02  9.1E-03  1.40E+01                                                   .......X.......

   4.282E-02  7.3E-03  1.50E+01                                                        .....X......

   4.638E-02  1.0E-02  1.60E+01                                                        ........X........


                      HELIX   BETA-SHEET    REMAINDER                SCALE FACTOR
 FRACTION              0.27         0.35         0.39                       0.555
 STANDARD ERROR     9.9E-03      2.1E-02      2.3E-02
0(FOR ALPHA/S(1) = 4.03E-02) PRUNS = 0.0066         PUNCOR =  0.2871  0.1746  0.0904  0.4055  0.9520
1CONTIN 2DP (MAR 84) ( CD-1 )    TEST DATA SET 1 - FOR CD PACKAGE
  CHOSEN SOLUTION

 WEIGHTED RESIDUALS (ALPHA/S(1)= 1.06E-03) MAX=U= 2.2E+00  MIN=L=-2.5E+00 (PRUNS= 0.0191) PUNCOR= 0.1674 0.0284
0.0008 0.1131 0.9623

 U---------+---------+---------+---------+---------*--
             *                               *
       *      *                        *
                      *                       *  *
  *                *      * *           *
      *                       **     **             *
            *          *    *
 0------*------*-----*-------------*---------*--------
    **   *        *                 **   *
   *            *       *       **
          *      *  *    **                *   **
                                 *

           *
                                                   *
 L---------+---------+---------+---------+-*-------+--
                    20                  40                  60                  80                 100
     120
```

```
0PLOT OF DATA (O) AND FIT TO DATA (X).  ORDINATES LISTED ARE FIT VALUES.

   ORDINATE  ABSCISSA
 -4.401E+02  2.40E+02                                    XO
 -5.022E+02  2.39E+02                                   OX
 -6.436E+02  2.38E+02                                  OX
 -8.396E+02  2.37E+02                                 *
 -1.052E+03  2.36E+02                               XO
 -1.294E+03  2.35E+02                             X O
 -1.530E+03  2.34E+02                           *
 -1.811E+03  2.33E+02                        OX
 -2.111E+03  2.32E+02                      *
 -2.434E+03  2.31E+02                  O X
 -2.761E+03  2.30E+02                 *
 -3.123E+03  2.29E+02              X O
 -3.436E+03  2.28E+02           X O
 -3.725E+03  2.27E+02         *
 -3.993E+03  2.26E+02      OX
 -4.237E+03  2.25E+02    OX
 -4.455E+03  2.24E+02   *
 -4.614E+03  2.23E+02  XO
 -4.720E+03  2.22E+02 OX
 -4.771E+03  2.21E+02 *
 -4.809E+03  2.20E+02 XO
```

```
-4.840E+03  2.19E+02    *
-4.858E+03  2.18E+02   *
-4.842E+03  2.17E+02   OX
-4.873E+03  2.16E+02  OX
-4.944E+03  2.15E+02   XO
-4.999E+03  2.14E+02  *
-5.078E+03  2.13E+02  *
-5.107E+03  2.12E+02 XO
-5.070E+03  2.11E+02  *
-5.030E+03  2.10E+02 OX
-5.023E+03  2.09E+02 OX
-4.925E+03  2.08E+02O  X
-4.815E+03  2.07E+02   OX
-4.617E+03  2.06E+02     *
-4.328E+03  2.05E+02       OX
-3.948E+03  2.04E+02           X O
-3.244E+03  2.03E+02                 X O
-2.105E+03  2.02E+02                          X   O
-6.384E+02  2.01E+02                                       X O
 8.944E+02  2.00E+02                                                   OX
 2.270E+03  1.99E+02                                                         O     X
 3.555E+03  1.98E+02                                                                       O  X
 4.797E+03  1.97E+02                                                                                    *
 5.931E+03  1.96E+02
```

```
  X   O
   6.958E+03  1.95E+02
          X  O
   7.696E+03  1.94E+02
              O X
   7.706E+03  1.93E+02
              O X
   7.172E+03  1.92E+02
            X  O
   5.799E+03  1.91E+02
 X    O
   3.401E+03  1.90E+02                                                                    O    X

   9.800E-01  0.00E+00                                               *

1CONTIN VERSION 2DP (MAR 1984) ( CD-1  PACKAGE)  ++++++++++++++++++++++++++++++++  CHOSEN SOLUTION
++++++++++++++++++++++++++++++++

           TEST DATA SET 1 - FOR CD PACKAGE

      ALPHA    ALPHA/S(1)     OBJ. FCTN.       VARIANCE      STD. DEV.    DEG FREEDOM    PROB1 TO REJECT    PROB2
TO REJECT
   4.47E-05      1.06E-03    7.45893E+01    5.75478E+01      1.223E+00        13.502             0.181
    0.992

    ORDINATE    ERROR  ABSCISSA
   3.422E-01  1.3E-01  1.00E+00                                                                       .....X.....

   6.103E-02  1.9E-01  2.00E+00                                                         .......X........

   1.595E-01  1.8E-01  3.00E+00                                                             .......X........

  -1.756E-01  7.3E-02  4.00E+00                                                    ...X...

  -4.889E-02  2.4E-01  5.00E+00                                                  ..........X..........

   2.720E-01  1.1E-01  6.00E+00                                                                    .....X....

   5.999E-01  8.7E-02  7.00E+00
...X....
  -6.827E-02  1.3E-01  8.00E+00                                                       .....X.....

   1.074E+00  1.5E-01  9.00E+00
          ......X
  -1.389E+00  2.5E-01  1.00E+01X..........

  -2.302E-01  7.4E-02  1.10E+01                                                  ...X...
```

```
 4.706E-01  1.1E-01  1.20E+01                                                                    ....X....
-2.810E-01  8.2E-02  1.30E+01                                               ...X....
-1.161E-01  1.3E-01  1.40E+01                                                    .....X.....
 2.335E-01  1.7E-01  1.50E+01                                                                .......X.......
 7.533E-02  1.8E-01  1.60E+01                                                          .......X.......

                    HELIX   BETA-SHEET    REMAINDER                SCALE FACTOR
FRACTION             0.20         0.75         0.05                       0.980
STANDARD ERROR     3.0E-02      4.2E-02      5.7E-02
```
