# Supplementary material for: Engineering of the E. coli Outer Membrane Protein FhuA to overcome the Hydrophobic Mismatch in Thick Polymeric Membranes
Source: J Nanobiotechnology. 2011 Mar 17;9:8. doi: 10.1186/1477-3155-9-8 (PMC3064644; doi:10.1186/1477-3155-9-8)
Supplement: Additional file 3 — Deconvolution analysis of labelled FhuA Δ1-159 Ext in octyl-pOE (detergent). CD spectra deconvolution analysis by the CONTIN algorithm of the FhuA Δ1-159 Ext (labelled) in octyl-pOE (detergent) solution. [file 1477-3155-9-8-S3.PDF]

```
 CONTIN - VERSION 2DP (MAR 1984) ( CD-1  PACK)       TEST DATA SET 1 - FOR CD PACKAGE


 REFERENCES - S.W. PROVENCHER (1982) COMPUT. PHYS. COMMUN., VOL. 27, PAGES 213-227, 229-242.
                              (1984) EMBL TECHNICAL REPORT DA07 (EUROPEAN MOLECULAR BIOLOGY LABORATORY,
HEIDELBERG, F.R. OF GERMANY)


                   INPUT DATA FOR CHANGES TO COMMON VARIABLES

 IFORMY    0    0.00000E+00
 (7F9.0)

 LAST      0    1.00000E+00

 IWT       0    5.00000E+00

 IUSER    14    3.10000E+01

 IUSER    15   -1.00000E+00

 RUSER    14    1.00000E+00

 RUSER    15    3.00000E-02

 RUSER    16    5.00000E+02

 END       0    0.00000E+00
 NSTEND   51    2.40000E+02    1.90000E+02



                                       FINAL VALUES OF CONTROL VARIABLES
 DFMIN  = 3.00000E+00
 SRMIN  = 1.00000E-02
 ALPST  = 0.00000E+00 0.00000E+00
 GMNMX  = 1.00000E+00 1.60000E+01
 PLEVEL = 5.00000E-01 5.00000E-01 5.00000E-01 5.00000E-01
 RSVMNX = 1.00000E+00 1.00000E+00 0.00000E+00 0.00000E+00
 RUSER  = 0.00000E+00 0.00000E+00 0.00000E+00 0.00000E+00 0.00000E+00 0.00000E+00 0.00000E+00 0.00000E+00 0.00000E+00 0.00000E+00
0.00000E+00 0.00000E+00
          0.00000E+00 0.00000E+00 0.00000E+00 1.00000E+00 3.00000E-02 5.00000E+02 0.00000E+00 0.00000E+00
0.00000E+00 0.00000E+00
          0.00000E+00 0.00000E+00 0.00000E+00 0.00000E+00 0.00000E+00 0.00000E+00 0.00000E+00 0.00000E+00
0.00000E+00 0.00000E+00
          0.00000E+00 0.00000E+00 0.00000E+00 0.00000E+00 0.00000E+00 0.00000E+00 0.00000E+00 0.00000E+00
0.00000E+00 0.00000E+00
```

```
          0.00000E+00 0.00000E+00 0.00000E+00 0.00000E+00 0.00000E+00 0.00000E+00 0.00000E+00 0.00000E+00
0.00000E+00 0.00000E+00
          0.00000E+00 0.00000E+00 0.00000E+00 0.00000E+00 0.00000E+00 0.00000E+00 0.00000E+00 0.00000E+00
0.00000E+00 0.00000E+00
          0.00000E+00 0.00000E+00 0.00000E+00 0.00000E+00 0.00000E+00 0.00000E+00 0.00000E+00 0.00000E+00
0.00000E+00 0.00000E+00
          0.00000E+00 0.00000E+00 0.00000E+00 0.00000E+00 0.00000E+00 0.00000E+00 0.00000E+00 0.00000E+00
0.00000E+00 0.00000E+00
          0.00000E+00 0.00000E+00 0.00000E+00 0.00000E+00 0.00000E+00 0.00000E+00 0.00000E+00 0.00000E+00
0.00000E+00 0.00000E+00
          0.00000E+00 0.00000E+00 0.00000E+00 0.00000E+00 0.00000E+00 0.00000E+00 0.00000E+00 0.00000E+00
0.00000E+00 0.00000E+00
 IGRID  =           1
 IQUAD  =           1
 IUNIT  =          -1
 IWT    =           5
 LINEPG =          60
 MIOERR =           5
 MPKMOM =           0
 MQPITR =          35
 NEQ    =           0
 NERFIT =           0
 NG     =          16
 NINTT  =           1
 NLINF  =           0
 NORDER =          -1
 ICRIT  =           1           1
 IFORMT = (5E15.6)
 IFORMW = (5E15.6)
 IFORMY = (7F9.0)
 IPLFIT =           2           2
 IPLRES =           2           2
 IPRINT =           2           3
 IUSER  =           0           0           0           0           0           0           0           0
 0           0
                    0           0           0          31          -1           4           7           0
 0           0
                    0           0           0           0           0           0           0           0
 0           0
                    0           0           0           0           0           0           0           0
 0           0
                    0           0           0           0           0           0           0           0
 0           0
 IUSROU =           3           3
 LSIGN  =           0           0           0           0           0           0           0           0
 0           0
                    0           0           0           0           0           0
```

```
MOMNMX =           0           0
NENDZ  =           0           0
NFLAT  =           0           0           0           0           0           0           0           0
NNSGN  =           0           0
NQPROG =           6           6
NSGN   =           0           0           0           0
DOCHOS =           T
DOMOM  =           F
DOUSIN =           T
DOUSNQ =           T
LAST   =           T
NEWPG1 =           F
NONNEG =           F
ONLY1  =           T
PRWT   =           T
PRY    =           T
SIMULA =           F
LUSER  =           F           F           F           F           F           F           F           F
 F           F
                   F           F           F           F           F           F           F           F
 F           F
                   F           F           F           F           F           F           F           F
 F           F


          T            Y            T            Y            T            Y            T            Y
 T            Y
   2.400E+02 -2.47000E+02    2.390E+02 -3.14000E+02    2.380E+02 -3.39000E+02    2.370E+02 -4.67000E+02
2.360E+02 -5.73000E+02
   2.350E+02 -6.55000E+02    2.340E+02 -8.58000E+02    2.330E+02 -1.01400E+03    2.320E+02 -1.18300E+03
2.310E+02 -1.35400E+03
   2.300E+02 -1.58200E+03    2.290E+02 -1.74200E+03    2.280E+02 -1.94600E+03    2.270E+02 -2.17900E+03
2.260E+02 -2.29900E+03
   2.250E+02 -2.34700E+03    2.240E+02 -2.46900E+03    2.230E+02 -2.50100E+03    2.220E+02 -2.51700E+03
2.210E+02 -2.51300E+03
   2.200E+02 -2.45100E+03    2.190E+02 -2.41800E+03    2.180E+02 -2.19200E+03    2.170E+02 -2.10500E+03
2.160E+02 -1.87700E+03
   2.150E+02 -1.71000E+03    2.140E+02 -1.41300E+03    2.130E+02 -1.17300E+03    2.120E+02 -9.88000E+02
2.110E+02 -8.27000E+02
   2.100E+02 -5.96000E+02    2.090E+02 -3.91000E+02    2.080E+02 -2.25000E+02    2.070E+02 -1.30000E+02
2.060E+02  4.00000E+01
   2.050E+02  1.89000E+02    2.040E+02  2.30000E+02    2.030E+02  4.00000E+02    2.020E+02  5.31000E+02
2.010E+02  6.41000E+02
   2.000E+02  6.81000E+02    1.990E+02  7.62000E+02    1.980E+02  8.41000E+02    1.970E+02  7.91000E+02
1.960E+02  8.43000E+02
   1.950E+02  1.00800E+03    1.940E+02  1.13700E+03    1.930E+02  1.00700E+03    1.920E+02  1.08800E+03
```

```
1.910E+02  1.27600E+03
    1.900E+02  1.07400E+03    0.000E+00  1.00000E+00
0PRECIS = 1.49E-15           SRANGE = 1.00E+35     RANGE = 1.00E+35


   GRID POINT     MIN IN MATRIX A    AT T =     MAX IN MATRIX A    AT T =     SCALE FACTOR
   1.0000E+00         -2.4876E+04  2.22E+02          5.5079E+04  1.92E+02        2.078E-06
   2.0000E+00         -1.3387E+04  2.08E+02          1.7434E+04  1.92E+02        2.078E-06
   3.0000E+00         -1.1109E+04  2.11E+02          1.6667E+04  0.00E+00        2.078E-06
   4.0000E+00         -1.2593E+04  2.09E+02          1.6667E+04  0.00E+00        2.078E-06
   5.0000E+00         -1.3227E+04  2.22E+02          2.4142E+04  1.93E+02        2.078E-06
   6.0000E+00         -9.7762E+03  2.03E+02          1.6667E+04  0.00E+00        2.078E-06
   7.0000E+00         -6.6914E+03  2.24E+02          1.6667E+04  0.00E+00        2.078E-06
   8.0000E+00         -1.2254E+04  2.23E+02          1.6667E+04  0.00E+00        2.078E-06
   9.0000E+00         -1.2473E+04  1.99E+02          1.6667E+04  0.00E+00        2.078E-06
   1.0000E+01         -1.1208E+04  2.09E+02          1.6667E+04  0.00E+00        2.078E-06
   1.1000E+01         -1.3714E+04  2.09E+02          2.5943E+04  1.96E+02        2.078E-06
   1.2000E+01         -1.4514E+04  2.22E+02          1.9732E+04  1.95E+02        2.078E-06
   1.3000E+01         -9.9252E+03  2.12E+02          1.6667E+04  0.00E+00        2.078E-06
   1.4000E+01         -1.8628E+04  2.03E+02          1.6667E+04  0.00E+00        2.078E-06
   1.5000E+01         -1.4012E+04  2.10E+02          2.8231E+04  1.92E+02        2.078E-06
   1.6000E+01         -1.1083E+04  2.11E+02          1.6667E+04  0.00E+00        2.078E-06
0SCALE FACTOR FOR ALPHA =  7.700E+06

   0 UNREGULARIZED VARIABLES

 SINGULAR VALUES
     4.036E-02    1.461E-02    6.268E-03    3.163E-03    1.902E-03    1.817E-03    1.383E-03    5.122E-04
4.872E-04    3.099E-04
     2.796E-04    1.190E-04    8.184E-05    6.539E-05    2.937E-05    2.580E-05
1
           TEST DATA SET 1 - FOR CD PACKAGE                                                        PRELIMINARY
UNWEIGHTED ANALYSIS

      ALPHA    ALPHA/S(1)     OBJ. FCTN.       VARIANCE      STD. DEV.    DEG FREEDOM    PROB1 TO REJECT    PROB2
TO REJECT
 * 6.01E-17      1.49E-15    2.52537E+05    2.52537E+05      8.262E+01        15.000              0.000
    1.000

                       HELIX   BETA-SHEET    REMAINDER                SCALE FACTOR
 FRACTION               0.00         0.62         0.38                       0.999
 STANDARD ERROR      3.3E-09      1.8E-02      1.8E-02
0(FOR ALPHA/S(1) = 1.49E-15) PRUNS = 0.0716         PUNCOR =  0.0012  0.1199  0.0005  0.0030  0.0725


           TEST DATA SET 1 - FOR CD PACKAGE                                                        PRELIMINARY
```

```
UNWEIGHTED ANALYSIS

      ALPHA    ALPHA/S(1)     OBJ. FCTN.       VARIANCE      STD. DEV.    DEG FREEDOM    PROB1 TO REJECT    PROB2
TO REJECT
 * 4.41E-14      1.09E-12    2.52537E+05    2.52537E+05      8.262E+01        15.000              0.000
    1.000

                       HELIX   BETA-SHEET    REMAINDER                SCALE FACTOR
 FRACTION               0.00         0.62         0.38                       0.999
 STANDARD ERROR      3.3E-09      1.8E-02      1.8E-02
0(FOR ALPHA/S(1) = 1.09E-12) PRUNS = 0.0716         PUNCOR =  0.0012  0.1199  0.0005  0.0030  0.0725


           TEST DATA SET 1 - FOR CD PACKAGE                                                          PRELIMINARY
UNWEIGHTED ANALYSIS

      ALPHA    ALPHA/S(1)     OBJ. FCTN.       VARIANCE      STD. DEV.    DEG FREEDOM    PROB1 TO REJECT    PROB2
TO REJECT
 * 3.23E-11      8.02E-10    2.52537E+05    2.52537E+05      8.262E+01        15.000              0.000
    1.000

                       HELIX   BETA-SHEET    REMAINDER                SCALE FACTOR
 FRACTION               0.00         0.62         0.38                       0.999
 STANDARD ERROR      3.3E-09      1.8E-02      1.8E-02
0(FOR ALPHA/S(1) = 8.02E-10) PRUNS = 0.0716         PUNCOR =  0.0012  0.1199  0.0005  0.0030  0.0725


           TEST DATA SET 1 - FOR CD PACKAGE                                                          PRELIMINARY
UNWEIGHTED ANALYSIS

      ALPHA    ALPHA/S(1)     OBJ. FCTN.       VARIANCE      STD. DEV.    DEG FREEDOM    PROB1 TO REJECT    PROB2
TO REJECT
 * 2.37E-08      5.88E-07    2.52537E+05    2.52537E+05      8.262E+01        15.000              0.000
    1.000

                       HELIX   BETA-SHEET    REMAINDER                SCALE FACTOR
 FRACTION               0.00         0.62         0.38                       0.999
 STANDARD ERROR      1.2E-08      1.8E-02      1.8E-02
0(FOR ALPHA/S(1) = 5.88E-07) PRUNS = 0.0716         PUNCOR =  0.0012  0.1199  0.0005  0.0030  0.0725


           TEST DATA SET 1 - FOR CD PACKAGE                                                          PRELIMINARY
UNWEIGHTED ANALYSIS
```

```
      ALPHA    ALPHA/S(1)     OBJ. FCTN.       VARIANCE      STD. DEV.    DEG FREEDOM    PROB1 TO REJECT    PROB2
TO REJECT
   1.74E-05      4.31E-04    3.16339E+05    2.55851E+05      8.256E+01         14.462              0.000
    0.716

                       HELIX   BETA-SHEET    REMAINDER                SCALE FACTOR
 FRACTION               0.00         0.61         0.39                       0.999
 STANDARD ERROR      5.1E-09      1.4E-02      1.4E-02
O(FOR ALPHA/S(1) = 4.31E-04) PRUNS = 0.0348         PUNCOR =  0.0008  0.1837  0.0007  0.0029  0.0426


           TEST DATA SET 1 - FOR CD PACKAGE                                                         PRELIMINARY
UNWEIGHTED ANALYSIS

      ALPHA    ALPHA/S(1)     OBJ. FCTN.       VARIANCE      STD. DEV.    DEG FREEDOM    PROB1 TO REJECT    PROB2
TO REJECT
   1.28E-02      3.16E-01    4.54563E+08    1.85001E+08      1.919E+03          1.786              1.000
    1.000

                       HELIX   BETA-SHEET    REMAINDER                SCALE FACTOR
 FRACTION               0.20         0.33         0.47                       0.438
 STANDARD ERROR      2.1E-02      1.8E-02      2.7E-02
O(FOR ALPHA/S(1) = 3.16E-01) PRUNS =-1.0000         PUNCOR =  0.0000  0.0001  0.0023  0.0237  0.1071


           TEST DATA SET 1 - FOR CD PACKAGE                                                         PRELIMINARY
UNWEIGHTED ANALYSIS

      ALPHA    ALPHA/S(1)     OBJ. FCTN.       VARIANCE      STD. DEV.    DEG FREEDOM    PROB1 TO REJECT    PROB2
TO REJECT
   4.47E-05      1.11E-03    6.00872E+05    3.07821E+05      8.913E+01         13.252              0.100
    0.980

                       HELIX   BETA-SHEET    REMAINDER                SCALE FACTOR
 FRACTION               0.00         0.59         0.41                       0.998
 STANDARD ERROR      2.2E-09      1.1E-02      1.0E-02
O(FOR ALPHA/S(1) = 1.11E-03) PRUNS = 0.0626         PUNCOR =  0.0003  0.6100  0.0051  0.0025  0.0093


           TEST DATA SET 1 - FOR CD PACKAGE                                                         PRELIMINARY
UNWEIGHTED ANALYSIS

      ALPHA    ALPHA/S(1)     OBJ. FCTN.       VARIANCE      STD. DEV.    DEG FREEDOM    PROB1 TO REJECT    PROB2
TO REJECT
```

```
   1.15E-04      2.84E-03    1.75250E+06    6.65950E+05      1.282E+02         11.490              1.000
    1.000

                      HELIX   BETA-SHEET    REMAINDER                SCALE FACTOR
 FRACTION              0.00         0.57         0.43                       0.992
 STANDARD ERROR      4.1E-09      1.2E-02      1.1E-02
O(FOR ALPHA/S(1) = 2.84E-03) PRUNS = 0.0048         PUNCOR =  0.0001  0.4439  0.1126  0.0070  0.0097



          TEST DATA SET 1 - FOR CD PACKAGE                                                   PRELIMINARY
UNWEIGHTED ANALYSIS

      ALPHA    ALPHA/S(1)     OBJ. FCTN.       VARIANCE      STD. DEV.    DEG FREEDOM    PROB1 TO REJECT    PROB2
TO REJECT
   2.94E-04      7.29E-03    6.30933E+06    1.63776E+06      1.960E+02          9.364              1.000
    1.000

                      HELIX   BETA-SHEET    REMAINDER                SCALE FACTOR
 FRACTION              0.00         0.55         0.45                       0.970
 STANDARD ERROR      9.9E-10      1.3E-02      1.2E-02
O(FOR ALPHA/S(1) = 7.29E-03) PRUNS = 0.0001         PUNCOR =  0.0000  0.0141  0.8289  0.1582  0.0942



          TEST DATA SET 1 - FOR CD PACKAGE                                                   PRELIMINARY
UNWEIGHTED ANALYSIS

      ALPHA    ALPHA/S(1)     OBJ. FCTN.       VARIANCE      STD. DEV.    DEG FREEDOM    PROB1 TO REJECT    PROB2
TO REJECT
   7.55E-04      1.87E-02    2.48693E+07    8.27704E+06      4.287E+02          6.970              1.000
    1.000

                      HELIX   BETA-SHEET    REMAINDER                SCALE FACTOR
 FRACTION              0.00         0.51         0.49                       0.886
 STANDARD ERROR      8.8E-10      1.8E-02      1.6E-02
O(FOR ALPHA/S(1) = 1.87E-02) PRUNS = 0.0000         PUNCOR =  0.0000  0.0000  0.0019  0.1881  0.7401



          TEST DATA SET 1 - FOR CD PACKAGE                                                   PRELIMINARY
UNWEIGHTED ANALYSIS

      ALPHA    ALPHA/S(1)     OBJ. FCTN.       VARIANCE      STD. DEV.    DEG FREEDOM    PROB1 TO REJECT    PROB2
TO REJECT
   1.94E-03      4.80E-02    7.55642E+07    3.48432E+07      8.583E+02          4.706              1.000
    1.000
```

```
                        HELIX   BETA-SHEET    REMAINDER                SCALE FACTOR
 FRACTION                0.00         0.47         0.53                       0.703
 STANDARD ERROR       1.0E-09      2.6E-02      2.7E-02
0(FOR ALPHA/S(1) = 4.80E-02) PRUNS = 0.0000         PUNCOR =  0.0001  0.0000  0.0045  0.1426  0.4874



          TEST DATA SET 1 - FOR CD PACKAGE                                                  PRELIMINARY
UNWEIGHTED ANALYSIS

      ALPHA    ALPHA/S(1)     OBJ. FCTN.       VARIANCE      STD. DEV.    DEG FREEDOM    PROB1 TO REJECT    PROB2
TO REJECT
   4.97E-03      1.23E-01    1.78822E+08    1.04277E+08      1.461E+03          3.128              1.000
    1.000

                        HELIX   BETA-SHEET    REMAINDER                SCALE FACTOR
 FRACTION                0.11         0.38         0.51                       0.481
 STANDARD ERROR       1.8E-02      2.8E-02      3.7E-02
0(FOR ALPHA/S(1) = 1.23E-01) PRUNS = 0.0000         PUNCOR =  0.0001  0.0002  0.0092  0.1038  0.3221
1CONTIN 2DP (MAR 84) ( CD-1 )    TEST DATA SET 1 - FOR CD PACKAGE
  CHOSEN SOLUTION

 WEIGHTED RESIDUALS (ALPHA/S(1)= 1.11E-03) MAX=U= 1.9E+02  MIN=L=-1.8E+02 (PRUNS= 0.0626) PUNCOR= 0.0003 0.6100
0.0051 0.0025 0.0093

 U---------+---------+-------*-+---------+---------+--
                              *

                            *        *          *
                               *       *           *
  * *  *     *                        *    *
     **     * *  *    *                     *        *
           *      *  *          *              *
 0-*------------*--**---*---------------*-----------*-
          *    *       *         *               *
        *                  *        *    **  *    *
         *
                          *                   *
                                  *
                         *
 L---------+---------+---------+---*-----+---------+--
                    20                  40                  60                  80                 100
     120
```

```
0PLOT OF DATA (O) AND FIT TO DATA (X).  ORDINATES LISTED ARE FIT VALUES.

    ORDINATE  ABSCISSA
  -3.082E+02  2.40E+02                                                                X O
  -3.121E+02  2.39E+02                                                                *
  -3.924E+02  2.38E+02                                                              XO
  -5.135E+02  2.37E+02                                                          X O
  -6.252E+02  2.36E+02                                                       XO
  -7.114E+02  2.35E+02                                                    XO
  -7.981E+02  2.34E+02                                               O X
  -9.265E+02  2.33E+02                                           O X
  -1.137E+03  2.32E+02                                      OX
  -1.365E+03  2.31E+02                                 *
  -1.611E+03  2.30E+02                          XO
  -1.816E+03  2.29E+02                    X O
  -1.982E+03  2.28E+02               XO
  -2.141E+03  2.27E+02         O X
  -2.296E+03  2.26E+02      *
  -2.396E+03  2.25E+02   X O
  -2.481E+03  2.24E+02 *
  -2.493E+03  2.23E+02*
  -2.501E+03  2.22E+02*
  -2.526E+03  2.21E+02*
  -2.496E+03  2.20E+02X O
  -2.390E+03  2.19E+02   *
```

```
  -2.185E+03  2.18E+02         *
  -1.953E+03  2.17E+02            O   X
  -1.771E+03  2.16E+02                  O  X
  -1.655E+03  2.15E+02                       OX
  -1.519E+03  2.14E+02                            X  O
  -1.363E+03  2.13E+02                                 X    O
  -1.163E+03  2.12E+02                                       X    O
  -9.099E+02  2.11E+02                                              X O
  -6.214E+02  2.10E+02                                                      XO
  -3.545E+02  2.09E+02                                                             OX
  -7.998E+01  2.08E+02                                                                 O    X
   5.487E+01  2.07E+02                                                                    O    X
   9.154E+01  2.06E+02                                                                         O X
   6.428E+01  2.05E+02                                                                          X  O
   1.686E+02  2.04E+02                                                                             X O
   3.119E+02  2.03E+02                                                                                 X O
   5.368E+02  2.02E+02                                                                                       *
   6.959E+02  2.01E+02                                                                                          O
X
   7.522E+02  2.00E+02
O X
   6.845E+02  1.99E+02
X O
   7.894E+02  1.98E+02
   XO
   8.534E+02  1.97E+02
   OX
   9.413E+02  1.96E+02
    O  X
   9.859E+02  1.95E+02
```

```
        XO
   1.028E+03  1.94E+02
         X   O
   1.031E+03  1.93E+02
         *
   1.146E+03  1.92E+02
           O X
   1.195E+03  1.91E+02
              X O
   1.093E+03  1.90E+02
           *
   9.975E-01  0.00E+00                                                                         *
```

```
 RMS RESIDUAL FOR PTS. 1 TO 31 =   7.32E+01
 RMS RESIDUAL FOR REMAINING PTS. = 8.36E+01


 ERRFIT = 0.00E+00
                             SQUARE ROOTS OF LEAST SQUARES WEIGHTS
    1.3656E-02   1.3656E-02   1.3656E-02   1.3656E-02   1.3656E-02   1.3656E-02   1.3656E-02   1.3656E-02
1.3656E-02   1.3656E-02
    1.3656E-02   1.3656E-02   1.3656E-02   1.3656E-02   1.3656E-02   1.3656E-02   1.3656E-02   1.3656E-02
1.3656E-02   1.3656E-02
    1.3656E-02   1.3656E-02   1.3656E-02   1.3656E-02   1.3656E-02   1.3656E-02   1.3656E-02   1.3656E-02
1.3656E-02   1.3656E-02
    1.3656E-02   1.1957E-02   1.1957E-02   1.1957E-02   1.1957E-02   1.1957E-02   1.1957E-02   1.1957E-02
1.1957E-02   1.1957E-02
    1.1957E-02   1.1957E-02   1.1957E-02   1.1957E-02   1.1957E-02   1.1957E-02   1.1957E-02   1.1957E-02
1.1957E-02   1.1957E-02
    1.1957E-02   3.3333E+01
```

GRID POINT	MIN IN MATRIX A	AT T =	MAX IN MATRIX A	AT T =	SCALE FACTOR
1.0000E+00	-3.3971E+02	2.22E+02	6.5858E+02	1.92E+02	1.667E-04
2.0000E+00	-1.7582E+02	2.10E+02	2.0845E+02	1.92E+02	1.667E-04
3.0000E+00	-1.5170E+02	2.11E+02	7.7487E+01	1.94E+02	1.667E-04
4.0000E+00	-1.7190E+02	2.10E+02	1.7697E+02	1.90E+02	1.667E-04
5.0000E+00	-1.8062E+02	2.22E+02	2.8867E+02	1.93E+02	1.667E-04
6.0000E+00	-1.1689E+02	2.03E+02	4.8685E+01	1.90E+02	1.667E-04
7.0000E+00	-9.1378E+01	2.24E+02	1.4343E+02	1.97E+02	1.667E-04
8.0000E+00	-1.6734E+02	2.23E+02	1.5497E+02	1.96E+02	1.667E-04
9.0000E+00	-1.4914E+02	1.99E+02	3.3333E+01	0.00E+00	1.667E-04
1.0000E+01	-1.5124E+02	2.10E+02	1.4815E+02	1.90E+02	1.667E-04
1.1000E+01	-1.8540E+02	2.10E+02	3.1020E+02	1.96E+02	1.667E-04
1.2000E+01	-1.9820E+02	2.22E+02	2.3593E+02	1.95E+02	1.667E-04

```
   1.3000E+01         -1.3554E+02  2.12E+02          1.7551E+02  1.98E+02        1.667E-04
   1.4000E+01         -2.2273E+02  2.03E+02          6.0625E+01  1.90E+02        1.667E-04
   1.5000E+01         -1.9135E+02  2.10E+02          3.3756E+02  1.92E+02        1.667E-04
   1.6000E+01         -1.5135E+02  2.11E+02          7.8445E+01  1.95E+02        1.667E-04
0SCALE FACTOR FOR ALPHA =  9.600E+04

   0 UNREGULARIZED VARIABLES

 SINGULAR VALUES
     4.054E-02    1.391E-02    5.646E-03    3.120E-03    1.941E-03    1.499E-03    6.003E-04    5.002E-04
4.423E-04    3.144E-04
     2.246E-04    1.099E-04    8.401E-05    6.120E-05    3.032E-05    2.643E-05
1
           TEST DATA SET 1 - FOR CD PACKAGE

      ALPHA    ALPHA/S(1)     OBJ. FCTN.       VARIANCE      STD. DEV.    DEG FREEDOM    PROB1 TO REJECT    PROB2
TO REJECT
 * 6.04E-17      1.49E-15    4.15653E+01    4.15653E+01      1.060E+00         15.000              0.000
    1.000

    ORDINATE    ERROR  ABSCISSA
   3.162E-01  9.7E-02  1.00E+00                                         .....X......

   5.164E-02  1.3E-01  2.00E+00                          ........X.......

  -5.244E-01  2.7E-01  3.00E+00...X...............

  -4.235E-01  5.2E-02  4.00E+00      ...X..

  -1.119E-01  1.9E-01  5.00E+00               ...........X..........

   8.649E-02  7.7E-02  6.00E+00                               .....X....

   5.832E-01  7.3E-02  7.00E+00                                                        ....X....

   6.443E-01  6.8E-02  8.00E+00                                                            ...X....

   1.230E+00  1.1E-01  9.00E+00
           .....X
  -5.884E-01  1.0E-01  1.00E+01X.....

  -3.452E-01  4.5E-02  1.10E+01          ...X..

  -1.035E-01  7.7E-02  1.20E+01                      ....X....

  -9.494E-02  5.7E-02  1.30E+01                       ....X...
```

```
 -1.673E-01  6.5E-02  1.40E+01                   ....X...

  6.149E-02  9.7E-02  1.50E+01                              .....X......

  3.536E-01  2.8E-01  1.60E+01                                    ...............X................


                     HELIX   BETA-SHEET    REMAINDER                SCALE FACTOR
 FRACTION             0.00         0.62         0.38                      0.968
 STANDARD ERROR      4.9E-09      3.0E-02      2.0E-02
0(FOR ALPHA/S(1) = 1.49E-15) PRUNS = 0.0716         PUNCOR =  0.0012  0.1310  0.0001  0.0023  0.1495


           TEST DATA SET 1 - FOR CD PACKAGE

      ALPHA    ALPHA/S(1)     OBJ. FCTN.       VARIANCE      STD. DEV.    DEG FREEDOM    PROB1 TO REJECT    PROB2
TO REJECT
 * 4.43E-14      1.09E-12    4.15653E+01    4.15653E+01      1.060E+00         15.000              0.000
   1.000

    ORDINATE    ERROR  ABSCISSA
   3.162E-01  9.7E-02  1.00E+00                                       .....X......

   5.164E-02  1.3E-01  2.00E+00                           ........X.......

  -5.244E-01  2.7E-01  3.00E+00...X...............

  -4.235E-01  5.2E-02  4.00E+00      ...X..

  -1.119E-01  1.9E-01  5.00E+00               ...........X..........

   8.649E-02  7.7E-02  6.00E+00                                .....X....

   5.832E-01  7.3E-02  7.00E+00                                                    ....X....

   6.443E-01  6.8E-02  8.00E+00                                                        ...X....

   1.230E+00  1.1E-01  9.00E+00
            .....X
  -5.884E-01  1.0E-01  1.00E+01X.....

  -3.452E-01  4.5E-02  1.10E+01          ...X..

  -1.035E-01  7.7E-02  1.20E+01                      ....X....

  -9.494E-02  5.7E-02  1.30E+01                       ....X...
```

```
 -1.673E-01  6.5E-02  1.40E+01                   ....X...

  6.149E-02  9.7E-02  1.50E+01                              .....X......

  3.536E-01  2.8E-01  1.60E+01                                    ...............X................


                    HELIX   BETA-SHEET    REMAINDER                SCALE FACTOR
FRACTION             0.00        0.62         0.38                       0.968
STANDARD ERROR    4.9E-09     3.0E-02      2.0E-02
0(FOR ALPHA/S(1) = 1.09E-12) PRUNS = 0.0716         PUNCOR =  0.0012  0.1310  0.0001  0.0023  0.1495


           TEST DATA SET 1 - FOR CD PACKAGE

      ALPHA    ALPHA/S(1)     OBJ. FCTN.       VARIANCE      STD. DEV.    DEG FREEDOM    PROB1 TO REJECT    PROB2
TO REJECT
 * 3.25E-11      8.02E-10    4.15653E+01    4.15653E+01      1.060E+00         15.000              0.000
    1.000

    ORDINATE    ERROR  ABSCISSA
  3.162E-01  9.7E-02  1.00E+00                                            .....X......

  5.164E-02  1.3E-01  2.00E+00                           ........X.......

 -5.244E-01  2.7E-01  3.00E+00...X...............

 -4.235E-01  5.2E-02  4.00E+00      ...X..

 -1.119E-01  1.9E-01  5.00E+00               ...........X..........

  8.649E-02  7.7E-02  6.00E+00                                .....X....

  5.832E-01  7.3E-02  7.00E+00                                                ....X....

  6.443E-01  6.8E-02  8.00E+00                                                    ...X....

  1.230E+00  1.1E-01  9.00E+00
           .....X
 -5.884E-01  1.0E-01  1.00E+01X.....

 -3.452E-01  4.5E-02  1.10E+01          ...X..

 -1.035E-01  7.7E-02  1.20E+01                      ....X....
```

```
  -9.494E-02  5.7E-02  1.30E+01                           ....X...

  -1.673E-01  6.5E-02  1.40E+01                       ....X...

   6.149E-02  9.7E-02  1.50E+01                                  .....X......

   3.536E-01  2.8E-01  1.60E+01                                        ...............X................


                     HELIX   BETA-SHEET    REMAINDER                SCALE FACTOR
 FRACTION             0.00         0.62         0.38                       0.968
 STANDARD ERROR      4.9E-09      3.0E-02      2.0E-02
0(FOR ALPHA/S(1) = 8.02E-10) PRUNS = 0.0716         PUNCOR =  0.0012  0.1310  0.0001  0.0023  0.1495



           TEST DATA SET 1 - FOR CD PACKAGE

      ALPHA    ALPHA/S(1)     OBJ. FCTN.       VARIANCE      STD. DEV.    DEG FREEDOM    PROB1 TO REJECT     PROB2
TO REJECT
 * 2.38E-08      5.88E-07    4.15653E+01    4.15653E+01      1.060E+00         15.000              0.000
    1.000

    ORDINATE    ERROR  ABSCISSA
   3.162E-01  9.7E-02  1.00E+00                                                .....X......

   5.164E-02  1.3E-01  2.00E+00                               ........X.......

  -5.244E-01  2.7E-01  3.00E+00...X...............

  -4.235E-01  5.2E-02  4.00E+00      ...X..

  -1.119E-01  1.9E-01  5.00E+00               ...........X..........

   8.649E-02  7.7E-02  6.00E+00                                .....X....

   5.832E-01  7.3E-02  7.00E+00                                                                ....X....

   6.443E-01  6.8E-02  8.00E+00                                                                    ...X....

   1.230E+00  1.1E-01  9.00E+00
         .....X
  -5.884E-01  1.0E-01  1.00E+01X.....

  -3.452E-01  4.5E-02  1.10E+01          ...X..

  -1.035E-01  7.7E-02  1.20E+01                     ....X....
```

```
  -9.494E-02  5.7E-02  1.30E+01                        ....X...

  -1.673E-01  6.5E-02  1.40E+01                    ....X...

   6.149E-02  9.7E-02  1.50E+01                               .....X......

   3.536E-01  2.8E-01  1.60E+01                                     ...............X................


                     HELIX   BETA-SHEET    REMAINDER                SCALE FACTOR
 FRACTION             0.00        0.62         0.38                        0.968
 STANDARD ERROR      4.7E-09     3.0E-02     2.0E-02
0(FOR ALPHA/S(1) = 5.88E-07) PRUNS = 0.0716         PUNCOR =  0.0012  0.1310  0.0001  0.0023  0.1495


          TEST DATA SET 1 - FOR CD PACKAGE

     ALPHA    ALPHA/S(1)     OBJ. FCTN.       VARIANCE      STD. DEV.    DEG FREEDOM    PROB1 TO REJECT    PROB2
TO REJECT
   1.75E-05      4.31E-04    5.05306E+01    4.21499E+01      1.060E+00         14.455             0.000
    0.724

    ORDINATE    ERROR  ABSCISSA
   2.656E-01  8.4E-02  1.00E+00                                          .....X.....

   2.927E-02  1.1E-01  2.00E+00                           ......X.......

  -4.124E-01  1.9E-01  3.00E+00.......X...........

  -4.198E-01  4.5E-02  4.00E+00    ..X...

  -3.886E-02  1.6E-01  5.00E+00                   .........X..........

   9.703E-02  6.2E-02  6.00E+00                                ...X....

   5.489E-01  6.2E-02  7.00E+00                                                          ....X...

   6.238E-01  6.4E-02  8.00E+00                                                              ....X....

   1.177E+00  9.7E-02  9.00E+00
          .....X
  -5.346E-01  9.5E-02  1.00E+01X.....

  -3.370E-01  4.0E-02  1.10E+01         ..X..
```

```
  -9.519E-02  6.1E-02  1.20E+01                        ...X....

  -1.110E-01  5.0E-02  1.30E+01                       ...X...

  -1.818E-01  5.9E-02  1.40E+01                   ...X....

   8.471E-02  8.6E-02  1.50E+01                                 .....X.....

   2.617E-01  1.9E-01  1.60E+01                                     ...........X..........


                      HELIX   BETA-SHEET    REMAINDER                SCALE FACTOR
 FRACTION              0.00         0.61         0.39                       0.957
 STANDARD ERROR      5.2E-09      2.8E-02      1.7E-02
0(FOR ALPHA/S(1) = 4.31E-04) PRUNS = 0.0239         PUNCOR =  0.0011  0.2206  0.0002  0.0022  0.1053



           TEST DATA SET 1 - FOR CD PACKAGE

      ALPHA    ALPHA/S(1)     OBJ. FCTN.       VARIANCE      STD. DEV.    DEG FREEDOM    PROB1 TO REJECT    PROB2
TO REJECT
   1.28E-02      3.16E-01    5.63253E+04    1.07701E+04      1.463E+01          1.710              1.000
    1.000

    ORDINATE    ERROR  ABSCISSA
  -2.721E-02  3.4E-03  1.00E+00X...

   1.574E-02  1.4E-03  2.00E+00                                                      .X..

   2.728E-02  1.8E-03  3.00E+00                                                                  ..X..

   2.133E-02  2.9E-03  4.00E+00                                                          ....X...

   1.706E-02  1.2E-03  5.00E+00                                                       ..X.

   3.669E-02  2.0E-03  6.00E+00                                                                            ...X..

   5.910E-02  2.1E-03  7.00E+00
            ..X
   2.981E-02  1.8E-03  8.00E+00                                                                     ..X..

   4.104E-02  2.5E-03  9.00E+00                                                                                 ...X..

   2.300E-02  1.3E-03  1.00E+01                                                              ..X.

   1.609E-02  2.1E-03  1.10E+01                                                     ...X..
```

```
   1.434E-02  2.3E-03  1.20E+01                                                 ...X..

   3.751E-02  2.3E-03  1.30E+01                                                                          ..X...

   1.584E-02  3.6E-03  1.40E+01                                                 ....X.....

   1.148E-02  1.6E-03  1.50E+01                                               .X..

   2.791E-02  1.8E-03  1.60E+01                                                                 ..X...


                       HELIX   BETA-SHEET    REMAINDER                SCALE FACTOR
 FRACTION               0.21         0.32         0.47                       0.367
 STANDARD ERROR      1.5E-02      1.2E-02      1.9E-02
0(FOR ALPHA/S(1) = 3.16E-01) PRUNS =-1.0000         PUNCOR =  0.0000  0.0000  0.0000  0.0000  0.0000



           TEST DATA SET 1 - FOR CD PACKAGE

      ALPHA    ALPHA/S(1)     OBJ. FCTN.       VARIANCE      STD. DEV.    DEG FREEDOM    PROB1 TO REJECT    PROB2
TO REJECT
   4.49E-05      1.11E-03    8.75749E+01    5.16452E+01      1.154E+00         13.201             0.143
    0.985

    ORDINATE    ERROR  ABSCISSA
   1.127E-01  5.5E-02  1.00E+00                                    .....X....

  -1.247E-03  7.1E-02  2.00E+00                           .....X.....

  -2.254E-01  8.4E-02  3.00E+00   ......X.......

  -3.696E-01  3.7E-02  4.00E+00X..

   1.288E-01  1.0E-01  5.00E+00                                  ........X.......

   9.682E-02  4.5E-02  6.00E+00                                    ...X....

   4.745E-01  4.1E-02  7.00E+00                                                             ...X...

   5.323E-01  5.5E-02  8.00E+00                                                                ....X....

   9.670E-01  7.8E-02  9.00E+00
           .....X
  -3.641E-01  7.3E-02  1.00E+01X.....
```

```
  -2.850E-01  3.4E-02  1.10E+01   ...X..
  -9.490E-02  4.0E-02  1.20E+01                 ...X...
  -1.490E-01  3.7E-02  1.30E+01             ...X...
  -1.880E-01  4.8E-02  1.40E+01         ....X....
   1.515E-01  6.8E-02  1.50E+01                                 .....X......
   1.265E-01  8.2E-02  1.60E+01                              .......X......

                     HELIX   BETA-SHEET    REMAINDER                SCALE FACTOR
 FRACTION             0.00         0.58         0.42                       0.913
 STANDARD ERROR      3.2E-09      2.6E-02      1.5E-02
0(FOR ALPHA/S(1) = 1.11E-03) PRUNS = 0.1730         PUNCOR =  0.0028  0.8224  0.0035  0.0032  0.0607


           TEST DATA SET 1 - FOR CD PACKAGE

      ALPHA    ALPHA/S(1)     OBJ. FCTN.       VARIANCE      STD. DEV.    DEG FREEDOM    PROB1 TO REJECT    PROB2
TO REJECT
   1.15E-04      2.84E-03    2.03543E+02    1.13056E+02      1.667E+00         11.308             1.000
    1.000

    ORDINATE    ERROR  ABSCISSA
  -7.192E-02  3.0E-02  1.00E+00              ....X....
  -2.003E-02  5.0E-02  2.00E+00                   ......X.......
  -9.084E-02  3.1E-02  3.00E+00           .....X....
  -2.082E-01  3.2E-02  4.00E+00X....
   1.895E-01  4.6E-02  5.00E+00                                              ......X.......
   5.916E-02  4.3E-02  6.00E+00                               .....X......
   4.257E-01  3.0E-02  7.00E+00
....X....
   3.117E-01  3.9E-02  8.00E+00                                                                .....X......
   5.162E-01  5.7E-02  9.00E+00
         .......X
  -1.240E-01  5.3E-02  1.00E+01    .......X.......
```

```
 -1.609E-01  3.2E-02  1.10E+01  ....X.....

 -1.103E-01  3.1E-02  1.20E+01         ....X....

 -1.317E-01  3.0E-02  1.30E+01      ....X....

 -7.664E-02  4.0E-02  1.40E+01            ......X.....

  2.378E-01  4.8E-02  1.50E+01                                                      .......X.......

  3.602E-02  3.2E-02  1.60E+01                             ....X.....

                    HELIX   BETA-SHEET    REMAINDER                SCALE FACTOR
 FRACTION            0.00        0.54         0.46                        0.782
 STANDARD ERROR    4.0E-09     3.4E-02      2.1E-02
0(FOR ALPHA/S(1) = 2.84E-03) PRUNS = 0.0042         PUNCOR =  0.0602  0.1814  0.2623  0.0270  0.1567


           TEST DATA SET 1 - FOR CD PACKAGE

      ALPHA    ALPHA/S(1)     OBJ. FCTN.       VARIANCE      STD. DEV.    DEG FREEDOM    PROB1 TO REJECT    PROB2
TO REJECT
   2.95E-04      7.29E-03    4.53287E+02    2.66328E+02      2.487E+00          8.926              1.000
    1.000

    ORDINATE    ERROR  ABSCISSA
 -8.530E-02  1.6E-02  1.00E+00....X...

 -1.943E-02  2.9E-02  2.00E+00            ......X.......

 -1.786E-02  1.5E-02  3.00E+00               ...X....

 -6.678E-02  2.5E-02  4.00E+00  ......X.....

  1.164E-01  2.5E-02  5.00E+00                                         ......X.....

  3.711E-02  3.1E-02  6.00E+00                        .......X......

  3.528E-01  2.3E-02  7.00E+00
          ....X
  1.504E-01  2.9E-02  8.00E+00                                                ......X.......

  1.540E-01  2.8E-02  9.00E+00                                                 ......X......
```

```
  4.377E-03  3.2E-02  1.00E+01                 .......X.......

 -1.048E-01  2.9E-02  1.10E+01X......

 -8.481E-02  2.4E-02  1.20E+01....X.....

 -4.654E-02  2.3E-02  1.30E+01       .....X.....

  2.652E-02  2.7E-02  1.40E+01                      ......X......

  1.470E-01  2.9E-02  1.50E+01                                                    .......X......

  5.004E-03  1.7E-02  1.60E+01                    ...X....


                    HELIX   BETA-SHEET    REMAINDER                SCALE FACTOR
 FRACTION            0.00         0.48         0.52                       0.568
 STANDARD ERROR    1.3E-09      3.8E-02      2.9E-02
0(FOR ALPHA/S(1) = 7.29E-03) PRUNS = 0.0048         PUNCOR =  0.1277  0.0094  0.8966  0.1475  0.4626


          TEST DATA SET 1 - FOR CD PACKAGE

     ALPHA    ALPHA/S(1)     OBJ. FCTN.       VARIANCE      STD. DEV.    DEG FREEDOM    PROB1 TO REJECT    PROB2
TO REJECT
   7.58E-04      1.87E-02    1.04650E+03    5.05520E+02      3.344E+00          6.798              1.000
    1.000

    ORDINATE    ERROR  ABSCISSA
 -3.241E-02  6.7E-03  1.00E+00          ..X..

 -2.785E-02  1.2E-02  2.00E+00         ....X....

  1.267E-02  1.0E-02  3.00E+00                      ...X...

  2.452E-02  1.4E-02  4.00E+00                         ....X....

  5.324E-02  8.6E-03  5.00E+00                                   ..X...

  4.321E-02  1.6E-02  6.00E+00                              ....X.....

  2.519E-01  1.7E-02  7.00E+00
          ....X
  8.368E-02  1.6E-02  8.00E+00                                              .....X.....

  3.223E-02  1.1E-02  9.00E+00                            ...X...
```

```
   5.666E-02  7.4E-03  1.00E+01                                    ..X...

  -7.886E-02  1.6E-02  1.10E+01X....

  -3.357E-02  1.5E-02  1.20E+01        ....X.....

  -8.955E-03  1.5E-02  1.30E+01               .....X....

  -1.394E-03  1.0E-02  1.40E+01                   ...X...

   1.646E-02  1.2E-02  1.50E+01                        ...X....

   1.045E-02  1.0E-02  1.60E+01                      ...X....


                       HELIX   BETA-SHEET    REMAINDER                SCALE FACTOR
 FRACTION               0.00         0.42         0.58                       0.402
 STANDARD ERROR      1.2E-02      3.8E-02      2.9E-02
0(FOR ALPHA/S(1) = 1.87E-02) PRUNS = 0.0002         PUNCOR =  0.0031  0.0006  0.2817  0.6268  0.6842



           TEST DATA SET 1 - FOR CD PACKAGE

      ALPHA    ALPHA/S(1)     OBJ. FCTN.       VARIANCE      STD. DEV.    DEG FREEDOM    PROB1 TO REJECT     PROB2
TO REJECT
   1.95E-03      4.80E-02    3.23228E+03    1.06293E+03      4.741E+00          4.706              1.000
    1.000

    ORDINATE    ERROR  ABSCISSA
  -2.590E-02  4.8E-03  1.00E+00       ..X..

  -2.321E-02  6.0E-03  2.00E+00       ...X...

   6.860E-03  7.3E-03  3.00E+00                      ....X...

   5.830E-02  8.0E-03  4.00E+00                                                ....X....

   3.413E-02  3.3E-03  5.00E+00                                     .X..

   2.616E-02  6.0E-03  6.00E+00                               ...X...

   1.512E-01  8.2E-03  7.00E+00
          ....X
   6.736E-02  6.5E-03  8.00E+00                                                     ...X...
```

```
   1.429E-02  3.7E-03  9.00E+00                                  ..X..

   3.432E-02  2.6E-03  1.00E+01                                             .X..

  -4.662E-02  7.5E-03  1.10E+01X...

   2.068E-04  7.8E-03  1.20E+01                    ....X....

   1.926E-02  9.5E-03  1.30E+01                             .....X.....

  -1.469E-02  5.1E-03  1.40E+01              ...X..

  -5.837E-05  4.9E-03  1.50E+01                      ..X...

   5.917E-03  7.2E-03  1.60E+01                       ....X....

                     HELIX   BETA-SHEET    REMAINDER                SCALE FACTOR
 FRACTION             0.08         0.34         0.58                       0.308
 STANDARD ERROR      1.1E-02      2.1E-02      2.6E-02
0(FOR ALPHA/S(1) = 4.80E-02) PRUNS = 0.0000         PUNCOR =  0.0000  0.0000  0.0004  0.0291  0.1434


           TEST DATA SET 1 - FOR CD PACKAGE

     ALPHA    ALPHA/S(1)     OBJ. FCTN.       VARIANCE      STD. DEV.    DEG FREEDOM    PROB1 TO REJECT    PROB2
TO REJECT
   5.00E-03      1.23E-01    1.24315E+04    2.95872E+03      7.768E+00          2.965              1.000
    1.000

    ORDINATE    ERROR  ABSCISSA
  -2.816E-02  4.5E-03  1.00E+00X...

  -2.555E-04  2.6E-03  2.00E+00                      ...X..

   1.466E-02  3.6E-03  3.00E+00                                   ...X...

   2.975E-02  4.9E-03  4.00E+00                                             ....X....

   2.063E-02  1.6E-03  5.00E+00                                        ..X.

   2.725E-02  2.6E-03  6.00E+00                                            ...X..

   8.695E-02  4.4E-03  7.00E+00
          ...X
   4.011E-02  3.6E-03  8.00E+00                                                     ...X...
```

```
   2.476E-02  2.5E-03  9.00E+00                                            ..X...
   2.101E-02  1.4E-03  1.00E+01                                          .X.
  -5.028E-03  4.3E-03  1.10E+01                ....X...
   8.036E-03  4.5E-03  1.20E+01                           ....X....
   3.574E-02  4.8E-03  1.30E+01                                                   ....X....
  -7.104E-03  3.7E-03  1.40E+01               ...X...
   6.668E-03  2.3E-03  1.50E+01                            ..X..
   1.528E-02  3.8E-03  1.60E+01                                  ...X...

                       HELIX   BETA-SHEET    REMAINDER                SCALE FACTOR
 FRACTION               0.16         0.32         0.52                       0.290
 STANDARD ERROR      1.2E-02      1.6E-02      2.5E-02
0(FOR ALPHA/S(1) = 1.23E-01) PRUNS = 0.0000         PUNCOR =  0.0000  0.0000  0.0000  0.0000  0.0022
1CONTIN 2DP (MAR 84) ( CD-1 )    TEST DATA SET 1 - FOR CD PACKAGE
  CHOSEN SOLUTION

 WEIGHTED RESIDUALS (ALPHA/S(1)= 1.11E-03) MAX=U= 2.9E+00  MIN=L=-2.1E+00 (PRUNS= 0.1730) PUNCOR= 0.0028 0.8224
0.0035 0.0032 0.0607

 U---------+---------+---------+---------+---------+-*
                             *
                              *
                                     *
                            *                   *
  *          *                 *       *   *       *
    ****      *  *    *               *     *
           **        *          *              *
 0-*------------*-***---*---------------*-----------*-
               *       *         *  *            *
        * *                *             **  *    *
         *                                    *
                          *
                         *        *
 L---------+---------+---------+---*-----+---------+--
                     20                  40                  60                  80                 100
      120
```

```
0PLOT OF DATA (O) AND FIT TO DATA (X).  ORDINATES LISTED ARE FIT VALUES.

    ORDINATE  ABSCISSA
  -3.071E+02  2.40E+02                                                               X O

  -3.128E+02  2.39E+02                                                               *

  -3.903E+02  2.38E+02                                                             XO

  -5.068E+02  2.37E+02                                                         X O

  -6.158E+02  2.36E+02                                                      XO

  -7.031E+02  2.35E+02                                                    XO

  -7.947E+02  2.34E+02                                               O X

  -9.273E+02  2.33E+02                                           O X

  -1.138E+03  2.32E+02                                      OX

  -1.366E+03  2.31E+02                                 *

  -1.610E+03  2.30E+02                          XO

  -1.815E+03  2.29E+02                    X O

  -1.982E+03  2.28E+02               XO

  -2.138E+03  2.27E+02         O X

  -2.291E+03  2.26E+02      *

  -2.389E+03  2.25E+02   X O

  -2.474E+03  2.24E+02 *

  -2.492E+03  2.23E+02*

  -2.502E+03  2.22E+02*

  -2.526E+03  2.21E+02*

  -2.497E+03  2.20E+02X O
```

```
  -2.395E+03  2.19E+02   *
  -2.197E+03  2.18E+02         *
  -1.969E+03  2.17E+02            O  X
  -1.783E+03  2.16E+02                  O  X
  -1.654E+03  2.15E+02                       O X
  -1.506E+03  2.14E+02                             X O
  -1.339E+03  2.13E+02                                  X   O
  -1.134E+03  2.12E+02                                       X    O
  -8.845E+02  2.11E+02                                               XO
  -6.020E+02  2.10E+02                                                       *
  -3.440E+02  2.09E+02                                                             OX
  -8.412E+01  2.08E+02                                                                 O    X
   4.422E+01  2.07E+02                                                                    O    X
   8.172E+01  2.06E+02                                                                         OX
   6.559E+01  2.05E+02                                                                          X  O
   1.715E+02  2.04E+02                                                                             X O
   3.181E+02  2.03E+02                                                                                 X O
   5.389E+02  2.02E+02                                                                                       *
   6.910E+02  2.01E+02                                                                                          O
X
   7.495E+02  2.00E+02
O X
   6.931E+02  1.99E+02
X O
   7.898E+02  1.98E+02
   XO
   8.522E+02  1.97E+02
   OX
   9.382E+02  1.96E+02
```

```
  O  X
 9.803E+02  1.95E+02
     XO
 1.029E+03  1.94E+02
      X   O
 1.038E+03  1.93E+02
      OX
 1.148E+03  1.92E+02
        O X
 1.194E+03  1.91E+02
           X O
 1.092E+03  1.90E+02
        *
 9.129E-01  0.00E+00                                                                        *

1CONTIN VERSION 2DP (MAR 1984) ( CD-1  PACKAGE)  ++++++++++++++++++++++++++++++++  CHOSEN SOLUTION
++++++++++++++++++++++++++++++++

           TEST DATA SET 1 - FOR CD PACKAGE

      ALPHA    ALPHA/S(1)     OBJ. FCTN.       VARIANCE      STD. DEV.    DEG FREEDOM    PROB1 TO REJECT    PROB2
TO REJECT
   4.49E-05      1.11E-03    8.75749E+01    5.16452E+01      1.154E+00        13.201              0.143
    0.985

    ORDINATE    ERROR  ABSCISSA
   1.127E-01  5.5E-02  1.00E+00                            .....X....

  -1.247E-03  7.1E-02  2.00E+00                   .....X.....

  -2.254E-01  8.4E-02  3.00E+00   ......X.......

  -3.696E-01  3.7E-02  4.00E+00X..

   1.288E-01  1.0E-01  5.00E+00                          ........X.......

   9.682E-02  4.5E-02  6.00E+00                            ...X....

   4.745E-01  4.1E-02  7.00E+00                                                         ...X...

   5.323E-01  5.5E-02  8.00E+00                                                            ....X....

   9.670E-01  7.8E-02  9.00E+00
           .....X
  -3.641E-01  7.3E-02  1.00E+01X.....

  -2.850E-01  3.4E-02  1.10E+01   ...X..
```

```
 -9.490E-02  4.0E-02  1.20E+01                   ...X...

 -1.490E-01  3.7E-02  1.30E+01               ...X...

 -1.880E-01  4.8E-02  1.40E+01           ....X....

  1.515E-01  6.8E-02  1.50E+01                                       .....X......

  1.265E-01  8.2E-02  1.60E+01                                    .......X......


                       HELIX   BETA-SHEET    REMAINDER                 SCALE FACTOR
FRACTION                0.00         0.58         0.42                        0.913
STANDARD ERROR       3.2E-09      2.6E-02      1.5E-02
```
