# Supplementary material for: Engineering of the E. coli Outer Membrane Protein FhuA to overcome the Hydrophobic Mismatch in Thick Polymeric Membranes
Source: J Nanobiotechnology. 2011 Mar 17;9:8. doi: 10.1186/1477-3155-9-8 (PMC3064644; doi:10.1186/1477-3155-9-8)
Supplement: Additional file 4 — Deconvolution analysis of the FhuA Δ1-159 Ext in Polymersomes. CD spectra deconvolution analysis by the CONTIN algorithm of the FhuA Δ1-159 Ext in poylmersomes. [file 1477-3155-9-8-S4.PDF]

```
 CONTIN - VERSION 2DP (MAR 1984) ( CD-1  PACK)       TEST DATA SET 1 - FOR CD
PACKAGE

 REFERENCES - S.W. PROVENCHER (1982) COMPUT. PHYS. COMMUN., VOL. 27, PAGES
213-227, 229-242.
                              (1984) EMBL TECHNICAL REPORT DA07 (EUROPEAN
MOLECULAR BIOLOGY LABORATORY, HEIDELBERG, F.R. OF GERMANY)


                    INPUT DATA FOR CHANGES TO COMMON VARIABLES

 IFORMY     0    0.00000E+00
 (7F9.0)

 LAST       0    1.00000E+00

 IWT        0    5.00000E+00

 IUSER     14    3.10000E+01

 IUSER     15   -1.00000E+00

 RUSER     14    1.00000E+00

 RUSER     15    3.00000E-02

 RUSER     16    5.00000E+02

 END        0    0.00000E+00
 NSTEND    51    2.40000E+02    1.90000E+02



                                                  FINAL VALUES OF CONTROL VARIABLES
 DFMIN  = 3.00000E+00
 SRMIN  = 1.00000E-02
 ALPST  = 0.00000E+00 0.00000E+00
 GMNMX  = 1.00000E+00 1.60000E+01
 PLEVEL = 5.00000E-01 5.00000E-01 5.00000E-01 5.00000E-01
 RSVMNX = 1.00000E+00 1.00000E+00 0.00000E+00 0.00000E+00
 RUSER  = 0.00000E+00 0.00000E+00 0.00000E+00 0.00000E+00 0.00000E+00
0.00000E+00 0.00000E+00 0.00000E+00 0.00000E+00 0.00000E+00
          0.00000E+00 0.00000E+00 0.00000E+00 1.00000E+00 3.00000E-02
5.00000E+02 0.00000E+00 0.00000E+00 0.00000E+00 0.00000E+00
          0.00000E+00 0.00000E+00 0.00000E+00 0.00000E+00 0.00000E+00
0.00000E+00 0.00000E+00 0.00000E+00 0.00000E+00 0.00000E+00
          0.00000E+00 0.00000E+00 0.00000E+00 0.00000E+00 0.00000E+00
0.00000E+00 0.00000E+00 0.00000E+00 0.00000E+00 0.00000E+00
          0.00000E+00 0.00000E+00 0.00000E+00 0.00000E+00 0.00000E+00
0.00000E+00 0.00000E+00 0.00000E+00 0.00000E+00 0.00000E+00
          0.00000E+00 0.00000E+00 0.00000E+00 0.00000E+00 0.00000E+00
0.00000E+00 0.00000E+00 0.00000E+00 0.00000E+00 0.00000E+00
          0.00000E+00 0.00000E+00 0.00000E+00 0.00000E+00 0.00000E+00
0.00000E+00 0.00000E+00 0.00000E+00 0.00000E+00 0.00000E+00
          0.00000E+00 0.00000E+00 0.00000E+00 0.00000E+00 0.00000E+00
0.00000E+00 0.00000E+00 0.00000E+00 0.00000E+00 0.00000E+00
          0.00000E+00 0.00000E+00 0.00000E+00 0.00000E+00 0.00000E+00
0.00000E+00 0.00000E+00 0.00000E+00 0.00000E+00 0.00000E+00
          0.00000E+00 0.00000E+00 0.00000E+00 0.00000E+00 0.00000E+00
0.00000E+00 0.00000E+00 0.00000E+00 0.00000E+00 0.00000E+00
 IGRID  =           1
 IQUAD  =           1
 IUNIT  =          -1
 IWT    =           5
 LINEPG =          60
 MIOERR =           5
 MPKMOM =           0
 MQPITR =          35
```

```
 NEQ    =           0
 NERFIT =           0
 NG     =          16
 NINTT  =           1
 NLINF  =           0
 NORDER =          -1
 ICRIT  =           1           1
 IFORMT = (5E15.6)
 IFORMW = (5E15.6)
 IFORMY = (7F9.0)
 IPLFIT =           2           2
 IPLRES =           2           2
 IPRINT =           2           3
 IUSER  =           0           0           0           0           0
0           0           0           0           0
            0           0           0          31          -1
4           7           0           0           0
            0           0           0           0           0
0           0           0           0           0
            0           0           0           0           0
0           0           0           0           0
            0           0           0           0           0
0           0           0           0           0
 IUSROU =           3           3
 LSIGN  =           0           0           0           0           0
0           0           0           0           0
            0           0           0           0           0
0
 MOMNMX =           0           0
 NENDZ  =           0           0
 NFLAT  =           0           0           0           0           0
0           0           0
 NNSGN  =           0           0
 NQPROG =           6           6
 NSGN   =           0           0           0           0
 DOCHOS =           T
 DOMOM  =           F
 DOUSIN =           T
 DOUSNQ =           T
 LAST   =           T
 NEWPG1 =           F
 NONNEG =           F
 ONLY1  =           T
 PRWT   =           T
 PRY    =           T
 SIMULA =           F
 LUSER  =           F           F           F           F           F
F           F           F           F           F
            F           F           F           F           F
F           F           F           F           F
            F           F           F           F           F
F           F           F           F           F


         T             Y             T             Y             T             Y
      T             Y             T             Y
   2.400E+02  0.00000E+00    2.390E+02 -3.60000E+01    2.380E+02 -1.16000E+02
 2.370E+02 -2.05000E+02    2.360E+02 -2.96000E+02
   2.350E+02 -4.20000E+02    2.340E+02 -5.25000E+02    2.330E+02 -5.44000E+02
 2.320E+02 -5.83000E+02    2.310E+02 -6.12000E+02
   2.300E+02 -6.20000E+02    2.290E+02 -5.83000E+02    2.280E+02 -5.96000E+02
 2.270E+02 -6.11000E+02    2.260E+02 -6.63000E+02
   2.250E+02 -7.09000E+02    2.240E+02 -8.07000E+02    2.230E+02 -8.84000E+02
 2.220E+02 -8.91000E+02    2.210E+02 -8.35000E+02
   2.200E+02 -8.70000E+02    2.190E+02 -8.64000E+02    2.180E+02 -8.67000E+02
 2.170E+02 -9.24000E+02    2.160E+02 -9.49000E+02
   2.150E+02 -9.64000E+02    2.140E+02 -9.29000E+02    2.130E+02 -9.00000E+02
```

```
 2.120E+02 -8.55000E+02    2.110E+02 -8.16000E+02
    2.100E+02 -7.49000E+02    2.090E+02 -7.54000E+02    2.080E+02 -7.42000E+02
 2.070E+02 -7.77000E+02    2.060E+02 -8.33000E+02
    2.050E+02 -8.09000E+02    2.040E+02 -7.70000E+02    2.030E+02 -7.16000E+02
 2.020E+02 -6.44000E+02    2.010E+02 -5.04000E+02
    2.000E+02 -3.21000E+02    1.990E+02 -1.48000E+02    1.980E+02 -2.50000E+01
 1.970E+02  1.28000E+02    1.960E+02  1.70000E+02
    1.950E+02  3.00000E+02    1.940E+02  1.70000E+02    1.930E+02  1.14000E+02
 1.920E+02  7.20000E+01    1.910E+02  6.50000E+01
    1.900E+02  1.16000E+02    0.000E+00  1.00000E+00
0PRECIS = 1.49E-15          SRANGE = 1.00E+35     RANGE = 1.00E+35


   GRID POINT     MIN IN MATRIX A    AT T =     MAX IN MATRIX A    AT T =
SCALE FACTOR
   1.0000E+00         -2.4876E+04  2.22E+02          5.5079E+04  1.92E+02
2.078E-06
   2.0000E+00         -1.3387E+04  2.08E+02          1.7434E+04  1.92E+02
2.078E-06
   3.0000E+00         -1.1109E+04  2.11E+02          1.6667E+04  0.00E+00
2.078E-06
   4.0000E+00         -1.2593E+04  2.09E+02          1.6667E+04  0.00E+00
2.078E-06
   5.0000E+00         -1.3227E+04  2.22E+02          2.4142E+04  1.93E+02
2.078E-06
   6.0000E+00         -9.7762E+03  2.03E+02          1.6667E+04  0.00E+00
2.078E-06
   7.0000E+00         -6.6914E+03  2.24E+02          1.6667E+04  0.00E+00
2.078E-06
   8.0000E+00         -1.2254E+04  2.23E+02          1.6667E+04  0.00E+00
2.078E-06
   9.0000E+00         -1.2473E+04  1.99E+02          1.6667E+04  0.00E+00
2.078E-06
   1.0000E+01         -1.1208E+04  2.09E+02          1.6667E+04  0.00E+00
2.078E-06
   1.1000E+01         -1.3714E+04  2.09E+02          2.5943E+04  1.96E+02
2.078E-06
   1.2000E+01         -1.4514E+04  2.22E+02          1.9732E+04  1.95E+02
2.078E-06
   1.3000E+01         -9.9252E+03  2.12E+02          1.6667E+04  0.00E+00
2.078E-06
   1.4000E+01         -1.8628E+04  2.03E+02          1.6667E+04  0.00E+00
2.078E-06
   1.5000E+01         -1.4012E+04  2.10E+02          2.8231E+04  1.92E+02
2.078E-06
   1.6000E+01         -1.1083E+04  2.11E+02          1.6667E+04  0.00E+00
2.078E-06
0SCALE FACTOR FOR ALPHA =  7.700E+06

   0 UNREGULARIZED VARIABLES

 SINGULAR VALUES
     4.036E-02    1.461E-02    6.268E-03    3.163E-03    1.902E-03    1.817E-03
  1.383E-03    5.122E-04    4.872E-04    3.099E-04
     2.796E-04    1.190E-04    8.184E-05    6.539E-05    2.937E-05    2.580E-05
1
           TEST DATA SET 1 - FOR CD PACKAGE
                   PRELIMINARY UNWEIGHTED ANALYSIS

      ALPHA    ALPHA/S(1)     OBJ. FCTN.       VARIANCE      STD. DEV.    DEG
FREEDOM    PROB1 TO REJECT    PROB2 TO REJECT
 * 6.01E-17      1.49E-15    2.61986E+05    2.61986E+05      8.415E+01
15.000            0.000             1.000

                       HELIX    BETA-SHEET    REMAINDER               SCALE
FACTOR
 FRACTION               0.00          0.69          0.31
0.999
```

```
 STANDARD ERROR      3.4E-09      1.8E-02      1.8E-02
0(FOR ALPHA/S(1) = 1.49E-15) PRUNS = 0.0066         PUNCOR =  0.0000  0.7078
0.0000  0.0000  0.0061


           TEST DATA SET 1 - FOR CD PACKAGE
                   PRELIMINARY UNWEIGHTED ANALYSIS

      ALPHA    ALPHA/S(1)     OBJ. FCTN.       VARIANCE      STD. DEV.    DEG
FREEDOM    PROB1 TO REJECT    PROB2 TO REJECT
 * 4.41E-14      1.09E-12    2.61986E+05    2.61986E+05      8.415E+01
15.000             0.000              1.000

                       HELIX   BETA-SHEET    REMAINDER                SCALE
FACTOR
 FRACTION              0.00         0.69         0.31
0.999
 STANDARD ERROR      3.4E-09      1.8E-02      1.8E-02
0(FOR ALPHA/S(1) = 1.09E-12) PRUNS = 0.0066         PUNCOR =  0.0000  0.7078
0.0000  0.0000  0.0061


           TEST DATA SET 1 - FOR CD PACKAGE
                   PRELIMINARY UNWEIGHTED ANALYSIS

      ALPHA    ALPHA/S(1)     OBJ. FCTN.       VARIANCE      STD. DEV.    DEG
FREEDOM    PROB1 TO REJECT    PROB2 TO REJECT
 * 3.23E-11      8.02E-10    2.61986E+05    2.61986E+05      8.415E+01
15.000             0.000              1.000

                       HELIX   BETA-SHEET    REMAINDER                SCALE
FACTOR
 FRACTION              0.00         0.69         0.31
0.999
 STANDARD ERROR      3.4E-09      1.8E-02      1.8E-02
0(FOR ALPHA/S(1) = 8.02E-10) PRUNS = 0.0066         PUNCOR =  0.0000  0.7078
0.0000  0.0000  0.0061


           TEST DATA SET 1 - FOR CD PACKAGE
                   PRELIMINARY UNWEIGHTED ANALYSIS

      ALPHA    ALPHA/S(1)     OBJ. FCTN.       VARIANCE      STD. DEV.    DEG
FREEDOM    PROB1 TO REJECT    PROB2 TO REJECT
 * 2.37E-08      5.88E-07    2.61986E+05    2.61986E+05      8.415E+01
15.000             0.000              1.000

                       HELIX   BETA-SHEET    REMAINDER                SCALE
FACTOR
 FRACTION              0.00         0.69         0.31
0.999
 STANDARD ERROR      1.3E-08      1.8E-02      1.8E-02
0(FOR ALPHA/S(1) = 5.88E-07) PRUNS = 0.0066         PUNCOR =  0.0000  0.7078
0.0000  0.0000  0.0061


           TEST DATA SET 1 - FOR CD PACKAGE
                   PRELIMINARY UNWEIGHTED ANALYSIS

      ALPHA    ALPHA/S(1)     OBJ. FCTN.       VARIANCE      STD. DEV.    DEG
FREEDOM    PROB1 TO REJECT    PROB2 TO REJECT
   1.74E-05      4.31E-04    3.41539E+05    2.66925E+05      8.433E+01
14.462             0.000              0.774
```

```
                       HELIX   BETA-SHEET    REMAINDER                SCALE
FACTOR
 FRACTION               0.00         0.68         0.32
0.999
 STANDARD ERROR      5.2E-09      1.5E-02      1.4E-02
0(FOR ALPHA/S(1) = 4.31E-04) PRUNS = 0.0011         PUNCOR =  0.0000  0.8804
0.0001  0.0000  0.0024


           TEST DATA SET 1 - FOR CD PACKAGE
                   PRELIMINARY UNWEIGHTED ANALYSIS

      ALPHA    ALPHA/S(1)     OBJ. FCTN.       VARIANCE      STD. DEV.    DEG
FREEDOM    PROB1 TO REJECT    PROB2 TO REJECT
   1.28E-02      3.16E-01    4.88902E+08    1.83138E+08      1.910E+03
1.786             1.000              1.000

                       HELIX   BETA-SHEET    REMAINDER                SCALE
FACTOR
 FRACTION               0.17         0.35         0.48
0.400
 STANDARD ERROR      2.2E-02      1.9E-02      2.9E-02
0(FOR ALPHA/S(1) = 3.16E-01) PRUNS =-1.0000         PUNCOR =  0.0070  0.0228
0.0627  0.1484  0.2994


           TEST DATA SET 1 - FOR CD PACKAGE
                   PRELIMINARY UNWEIGHTED ANALYSIS

      ALPHA    ALPHA/S(1)     OBJ. FCTN.       VARIANCE      STD. DEV.    DEG
FREEDOM    PROB1 TO REJECT    PROB2 TO REJECT
   4.47E-05      1.11E-03    6.86309E+05    3.34417E+05      9.290E+01
13.252             0.215              0.992

                       HELIX   BETA-SHEET    REMAINDER                SCALE
FACTOR
 FRACTION               0.00         0.65         0.35
0.997
 STANDARD ERROR      2.3E-09      1.1E-02      1.0E-02
0(FOR ALPHA/S(1) = 1.11E-03) PRUNS = 0.0010         PUNCOR =  0.0000  0.6522
0.0011  0.0000  0.0006


           TEST DATA SET 1 - FOR CD PACKAGE
                   PRELIMINARY UNWEIGHTED ANALYSIS

      ALPHA    ALPHA/S(1)     OBJ. FCTN.       VARIANCE      STD. DEV.    DEG
FREEDOM    PROB1 TO REJECT    PROB2 TO REJECT
   1.15E-04      2.84E-03    2.07035E+06    7.52196E+05      1.363E+02
11.490             1.000              1.000

                       HELIX   BETA-SHEET    REMAINDER                SCALE
FACTOR
 FRACTION               0.00         0.63         0.37
0.991
 STANDARD ERROR      4.3E-09      1.2E-02      1.2E-02
0(FOR ALPHA/S(1) = 2.84E-03) PRUNS = 0.0002         PUNCOR =  0.0000  0.2413
0.0685  0.0030  0.0037


           TEST DATA SET 1 - FOR CD PACKAGE
                   PRELIMINARY UNWEIGHTED ANALYSIS

      ALPHA    ALPHA/S(1)     OBJ. FCTN.       VARIANCE      STD. DEV.    DEG
```

```
FREEDOM    PROB1 TO REJECT    PROB2 TO REJECT
   2.94E-04      7.29E-03    7.68181E+06    1.92527E+06      2.125E+02
9.364             1.000              1.000

                       HELIX   BETA-SHEET    REMAINDER                SCALE
FACTOR
 FRACTION               0.00         0.60         0.40
0.966
 STANDARD ERROR      1.1E-09      1.4E-02      1.3E-02
0(FOR ALPHA/S(1) = 7.29E-03) PRUNS = 0.0001         PUNCOR =  0.0000  0.0182
0.8969  0.2104  0.0720


           TEST DATA SET 1 - FOR CD PACKAGE
                   PRELIMINARY UNWEIGHTED ANALYSIS

      ALPHA    ALPHA/S(1)     OBJ. FCTN.       VARIANCE      STD. DEV.    DEG
FREEDOM    PROB1 TO REJECT    PROB2 TO REJECT
   7.55E-04      1.87E-02    3.04272E+07    1.01678E+07      4.752E+02
6.970             1.000              1.000

                       HELIX   BETA-SHEET    REMAINDER                SCALE
FACTOR
 FRACTION               0.00         0.56         0.44
0.868
 STANDARD ERROR      1.0E-09      2.1E-02      1.9E-02
0(FOR ALPHA/S(1) = 1.87E-02) PRUNS = 0.0000         PUNCOR =  0.0000  0.0000
0.0038  0.1401  0.7608


           TEST DATA SET 1 - FOR CD PACKAGE
                   PRELIMINARY UNWEIGHTED ANALYSIS

      ALPHA    ALPHA/S(1)     OBJ. FCTN.       VARIANCE      STD. DEV.    DEG
FREEDOM    PROB1 TO REJECT    PROB2 TO REJECT
   1.94E-03      4.80E-02    8.77113E+07    4.68932E+07      9.958E+02
4.706             1.000              1.000

                       HELIX   BETA-SHEET    REMAINDER                SCALE
FACTOR
 FRACTION               0.00         0.51         0.49
0.655
 STANDARD ERROR      1.3E-09      3.2E-02      3.3E-02
0(FOR ALPHA/S(1) = 4.80E-02) PRUNS =-1.0000         PUNCOR =  0.0002  0.0005
0.0053  0.0658  0.3439


           TEST DATA SET 1 - FOR CD PACKAGE
                   PRELIMINARY UNWEIGHTED ANALYSIS

      ALPHA    ALPHA/S(1)     OBJ. FCTN.       VARIANCE      STD. DEV.    DEG
FREEDOM    PROB1 TO REJECT    PROB2 TO REJECT
   4.97E-03      1.23E-01    1.89035E+08    1.14913E+08      1.533E+03
3.128             1.000              1.000

                       HELIX   BETA-SHEET    REMAINDER                SCALE
FACTOR
 FRACTION               0.08         0.43         0.50
0.434
 STANDARD ERROR      2.1E-02      3.2E-02      4.3E-02
0(FOR ALPHA/S(1) = 1.23E-01) PRUNS =-1.0000         PUNCOR =  0.0066  0.0258
0.0915  0.2557  0.5126
1CONTIN 2DP (MAR 84) ( CD-1 )    TEST DATA SET 1 - FOR CD PACKAGE
                                   CHOSEN SOLUTION
```

```
                               FhuAExt-POlymersoem
 WEIGHTED RESIDUALS (ALPHA/S(1)= 1.11E-03) MAX=U= 1.8E+02  MIN=L=-1.5E+02
(PRUNS= 0.0010) PUNCOR= 0.0000 0.6522 0.0011 0.0000 0.0006

 U---------+---------+--------*+---------+---------+--
  *                          *
                                      **
                               *
   *
    *        * **            *              *     *
     *        *  *   ***        *    *               *
      *     *                           *      *  *
 O------------------------------------------*---*---*-
           *      *     *                    *     *
       *  *                *
                                 *        *
         *          *                    *    *
        *          *      *         *
                         *        *
 L---------+---------+---------+---*-----+---------+--
                     20                  40                  60
80                 100                 120


0PLOT OF DATA (O) AND FIT TO DATA (X).  ORDINATES LISTED ARE FIT VALUES.

    ORDINATE  ABSCISSA
  -1.508E+02  2.40E+02
             X           O
  -1.314E+02  2.39E+02
               X       O
  -1.749E+02  2.38E+02
           X    O
  -2.527E+02  2.37E+02
     X   O
  -3.292E+02  2.36E+02
X  O
  -3.837E+02  2.35E+02                                                   O  X

  -4.216E+02  2.34E+02                                          O        X

  -4.651E+02  2.33E+02                                         O     X

  -5.380E+02  2.32E+02                                      O  X

  -5.980E+02  2.31E+02                                   O X

  -6.460E+02  2.30E+02                                 X O

  -6.545E+02  2.29E+02                                X     O

  -6.478E+02  2.28E+02                                 X   O

  -6.677E+02  2.27E+02                               X    O

  -7.204E+02  2.26E+02                           X   O

  -7.481E+02  2.25E+02                         X  O

  -7.818E+02  2.24E+02                    O X

  -7.763E+02  2.23E+02              O       X

  -8.038E+02  2.22E+02             O      X

  -8.756E+02  2.21E+02              X   O

  -9.208E+02  2.20E+02           X   O
```

```
  -9.161E+02  2.19E+02           X   O
  -8.534E+02  2.18E+02               OX
  -8.022E+02  2.17E+02          O         X
  -8.299E+02  2.16E+02        O         X
  -9.246E+02  2.15E+02       O  X
  -1.006E+03  2.14E+02    X     O
  -1.061E+03  2.13E+02X           O
  -1.031E+03  2.12E+02  X             O
  -9.285E+02  2.11E+02          X        O
  -8.022E+02  2.10E+02                    X   O
  -6.968E+02  2.09E+02                        O    X
  -6.016E+02  2.08E+02                         O          X
  -6.241E+02  2.07E+02                      O           X
  -7.144E+02  2.06E+02                  O        X
  -8.594E+02  2.05E+02                X   O
  -8.989E+02  2.04E+02            X          O
  -8.441E+02  2.03E+02                 X         O
  -6.561E+02  2.02E+02                                XO
  -4.241E+02  2.01E+02                                            O      X
  -2.629E+02  2.00E+02
O   X
  -2.049E+02  1.99E+02
         X    O
  -2.412E+01  1.98E+02
                       OX
   1.385E+02  1.97E+02
                                    OX
   2.503E+02  1.96E+02
                                       O      X
   2.758E+02  1.95E+02
                                                XO
   1.685E+02  1.94E+02
                                       *
   5.081E+01  1.93E+02
                              X    O
   4.854E+01  1.92E+02
                             X O
   7.907E+01  1.91E+02
                               OX
   1.222E+02  1.90E+02
                                   *
   9.974E-01  0.00E+00
                          *


 RMS RESIDUAL FOR PTS. 1 TO 31 =   8.26E+01
 RMS RESIDUAL FOR REMAINING PTS. = 7.78E+01
```

```
 ERRFIT = 0.00E+00
                                   SQUARE ROOTS OF LEAST SQUARES WEIGHTS
    1.2385E-02   1.2385E-02   1.2385E-02   1.2385E-02   1.2385E-02   1.2385E-02
 1.2385E-02   1.2385E-02   1.2385E-02   1.2385E-02
    1.2385E-02   1.2385E-02   1.2385E-02   1.2385E-02   1.2385E-02   1.2385E-02
 1.2385E-02   1.2385E-02   1.2385E-02   1.2385E-02
    1.2385E-02   1.2385E-02   1.2385E-02   1.2385E-02   1.2385E-02   1.2385E-02
 1.2385E-02   1.2385E-02   1.2385E-02   1.2385E-02
    1.2385E-02   1.2385E-02   1.2385E-02   1.2385E-02   1.2385E-02   1.2385E-02
 1.2385E-02   1.2385E-02   1.2385E-02   1.2385E-02
    1.2385E-02   1.2385E-02   1.2385E-02   1.2385E-02   1.2385E-02   1.2385E-02
 1.2385E-02   1.2385E-02   1.2385E-02   1.2385E-02
    1.2385E-02   3.3333E+01


   GRID POINT     MIN IN MATRIX A    AT T =     MAX IN MATRIX A    AT T =
SCALE FACTOR
   1.0000E+00         -3.0810E+02  2.22E+02         6.8217E+02  1.92E+02
1.728E-04
   2.0000E+00         -1.6580E+02  2.08E+02         2.1592E+02  1.92E+02
1.728E-04
   3.0000E+00         -1.3758E+02  2.11E+02         8.0263E+01  1.94E+02
1.728E-04
   4.0000E+00         -1.5597E+02  2.09E+02         1.8331E+02  1.90E+02
1.728E-04
   5.0000E+00         -1.6382E+02  2.22E+02         2.9901E+02  1.93E+02
1.728E-04
   6.0000E+00         -1.2108E+02  2.03E+02         5.0429E+01  1.90E+02
1.728E-04
   7.0000E+00         -8.2876E+01  2.24E+02         1.4857E+02  1.97E+02
1.728E-04
   8.0000E+00         -1.5177E+02  2.23E+02         1.6052E+02  1.96E+02
1.728E-04
   9.0000E+00         -1.5448E+02  1.99E+02         3.3333E+01  0.00E+00
1.728E-04
   1.0000E+01         -1.3881E+02  2.09E+02         1.5346E+02  1.90E+02
1.728E-04
   1.1000E+01         -1.6986E+02  2.09E+02         3.2131E+02  1.96E+02
1.728E-04
   1.2000E+01         -1.7976E+02  2.22E+02         2.4438E+02  1.95E+02
1.728E-04
   1.3000E+01         -1.2293E+02  2.12E+02         1.8180E+02  1.98E+02
1.728E-04
   1.4000E+01         -2.3071E+02  2.03E+02         6.2797E+01  1.90E+02
1.728E-04
   1.5000E+01         -1.7354E+02  2.10E+02         3.4965E+02  1.92E+02
1.728E-04
   1.6000E+01         -1.3727E+02  2.11E+02         8.1255E+01  1.95E+02
1.728E-04
0SCALE FACTOR FOR ALPHA =  9.260E+04

   0 UNREGULARIZED VARIABLES

 SINGULAR VALUES
     4.089E-02    1.465E-02    5.926E-03    3.164E-03    1.914E-03    1.448E-03
  6.074E-04    5.172E-04    4.533E-04    3.116E-04
     2.284E-04    1.123E-04    8.377E-05    6.475E-05    2.992E-05    2.657E-05
1
           TEST DATA SET 1 - FOR CD PACKAGE


      ALPHA    ALPHA/S(1)     OBJ. FCTN.       VARIANCE      STD. DEV.    DEG
FREEDOM    PROB1 TO REJECT    PROB2 TO REJECT
 * 6.09E-17      1.49E-15    3.93007E+01    3.93007E+01      1.031E+00
15.000             0.000              1.000

    ORDINATE    ERROR  ABSCISSA
```

```
   3.022E-01  9.4E-02  1.00E+00
.....X....
   3.314E-01  1.3E-01  2.00E+00
......X......
  -6.803E-01  2.7E-01  3.00E+00......X...........

  -4.219E-01  5.4E-02  4.00E+00               ...X..

  -1.020E-01  1.9E-01  5.00E+00                        ........X.........

   8.001E-02  7.7E-02  6.00E+00                                     ...X....

   5.318E-01  7.4E-02  7.00E+00
        ...X...
   6.375E-01  7.0E-02  8.00E+00
             ...X...
   1.375E+00  1.0E-01  9.00E+00
                                              ....X
  -8.172E-01  1.0E-01  1.00E+01X....

  -1.504E-01  4.6E-02  1.10E+01                            ..X..

  -2.389E-01  7.6E-02  1.20E+01                      ....X...

  -1.407E-01  5.6E-02  1.30E+01                            ..X...

  -2.279E-01  6.7E-02  1.40E+01                       ...X...

  -2.967E-02  9.7E-02  1.50E+01                               ....X.....

   5.231E-01  2.7E-01  1.60E+01
.............X...........

                       HELIX   BETA-SHEET    REMAINDER                SCALE
FACTOR
 FRACTION               0.00         0.69         0.31
0.972
 STANDARD ERROR      9.9E-09      3.0E-02      2.0E-02
0(FOR ALPHA/S(1) = 1.49E-15) PRUNS = 0.0066         PUNCOR =  0.0000  0.6415
0.0000  0.0000  0.0064


           TEST DATA SET 1 - FOR CD PACKAGE


      ALPHA    ALPHA/S(1)     OBJ. FCTN.       VARIANCE      STD. DEV.    DEG
FREEDOM    PROB1 TO REJECT    PROB2 TO REJECT
 * 4.47E-14      1.09E-12    3.93007E+01    3.93007E+01      1.031E+00
15.000             0.000              1.000

    ORDINATE    ERROR  ABSCISSA
   3.022E-01  9.4E-02  1.00E+00
.....X....
   3.314E-01  1.3E-01  2.00E+00
......X......
  -6.803E-01  2.7E-01  3.00E+00......X...........

  -4.219E-01  5.4E-02  4.00E+00               ...X..

  -1.020E-01  1.9E-01  5.00E+00                        ........X.........

   8.001E-02  7.7E-02  6.00E+00                                     ...X....

   5.318E-01  7.4E-02  7.00E+00
        ...X...
   6.375E-01  7.0E-02  8.00E+00
             ...X...
```

```
   1.375E+00  1.0E-01  9.00E+00
                                             ....X
  -8.172E-01  1.0E-01  1.00E+01X....

  -1.504E-01  4.6E-02  1.10E+01                            ..X..

  -2.389E-01  7.6E-02  1.20E+01                      ....X...

  -1.407E-01  5.6E-02  1.30E+01                            ..X...

  -2.279E-01  6.7E-02  1.40E+01                       ...X...

  -2.967E-02  9.7E-02  1.50E+01                               ....X.....

   5.231E-01  2.7E-01  1.60E+01
.............X...........

                       HELIX   BETA-SHEET    REMAINDER                SCALE
FACTOR
 FRACTION               0.00         0.69         0.31
0.972
 STANDARD ERROR      9.9E-09      3.0E-02      2.0E-02
0(FOR ALPHA/S(1) = 1.09E-12) PRUNS = 0.0066         PUNCOR =  0.0000  0.6415
0.0000  0.0000  0.0064



           TEST DATA SET 1 - FOR CD PACKAGE


      ALPHA    ALPHA/S(1)     OBJ. FCTN.       VARIANCE      STD. DEV.    DEG
FREEDOM    PROB1 TO REJECT    PROB2 TO REJECT
 * 3.28E-11      8.02E-10    3.93007E+01    3.93007E+01      1.031E+00
15.000             0.000              1.000

    ORDINATE    ERROR  ABSCISSA
   3.022E-01  9.4E-02  1.00E+00
.....X....
   3.314E-01  1.3E-01  2.00E+00
......X......
  -6.803E-01  2.7E-01  3.00E+00......X............

  -4.219E-01  5.4E-02  4.00E+00               ...X..

  -1.020E-01  1.9E-01  5.00E+00                        ........X.........

   8.001E-02  7.7E-02  6.00E+00                                     ...X....

   5.318E-01  7.4E-02  7.00E+00
        ...X...
   6.375E-01  7.0E-02  8.00E+00
             ...X...
   1.375E+00  1.0E-01  9.00E+00
                                             ....X
  -8.172E-01  1.0E-01  1.00E+01X....

  -1.504E-01  4.6E-02  1.10E+01                            ..X..

  -2.389E-01  7.6E-02  1.20E+01                      ....X...

  -1.407E-01  5.6E-02  1.30E+01                            ..X...

  -2.279E-01  6.7E-02  1.40E+01                       ...X...

  -2.967E-02  9.7E-02  1.50E+01                               ....X.....

   5.231E-01  2.7E-01  1.60E+01
.............X...........
```

```
                        HELIX   BETA-SHEET    REMAINDER                SCALE
FACTOR
 FRACTION                0.00         0.69         0.31
0.972
 STANDARD ERROR      9.9E-09      3.0E-02      2.0E-02
0(FOR ALPHA/S(1) = 8.02E-10) PRUNS = 0.0066         PUNCOR =  0.0000  0.6415
0.0000  0.0000  0.0064


           TEST DATA SET 1 - FOR CD PACKAGE


      ALPHA    ALPHA/S(1)     OBJ. FCTN.       VARIANCE      STD. DEV.    DEG
FREEDOM    PROB1 TO REJECT    PROB2 TO REJECT
 * 2.40E-08      5.88E-07    3.93007E+01    3.93007E+01      1.031E+00
15.000             0.000              1.000

    ORDINATE    ERROR  ABSCISSA
   3.022E-01  9.4E-02  1.00E+00
.....X....
   3.314E-01  1.3E-01  2.00E+00
......X......
  -6.803E-01  2.7E-01  3.00E+00......X...........

  -4.219E-01  5.4E-02  4.00E+00              ...X..

  -1.020E-01  1.9E-01  5.00E+00                       ........X.........

   8.001E-02  7.7E-02  6.00E+00                                    ...X....

   5.318E-01  7.4E-02  7.00E+00
        ...X...
   6.375E-01  7.0E-02  8.00E+00
             ...X...
   1.375E+00  1.0E-01  9.00E+00
                                             ....X
  -8.172E-01  1.0E-01  1.00E+01X....

  -1.504E-01  4.6E-02  1.10E+01                           ..X..

  -2.389E-01  7.6E-02  1.20E+01                     ....X...

  -1.407E-01  5.6E-02  1.30E+01                           ..X...

  -2.279E-01  6.7E-02  1.40E+01                      ...X...

  -2.967E-02  9.7E-02  1.50E+01                              ....X.....

   5.231E-01  2.7E-01  1.60E+01
............X...........

                        HELIX   BETA-SHEET    REMAINDER                SCALE
FACTOR
 FRACTION                0.00         0.69         0.31
0.972
 STANDARD ERROR      7.4E-09      3.0E-02      2.0E-02
0(FOR ALPHA/S(1) = 5.88E-07) PRUNS = 0.0066         PUNCOR =  0.0000  0.6415
0.0000  0.0000  0.0064


           TEST DATA SET 1 - FOR CD PACKAGE


      ALPHA    ALPHA/S(1)     OBJ. FCTN.       VARIANCE      STD. DEV.    DEG
FREEDOM    PROB1 TO REJECT    PROB2 TO REJECT
```

```
   1.76E-05      4.31E-04    5.02120E+01    4.01209E+01      1.034E+00
14.461             0.000              0.789

    ORDINATE    ERROR  ABSCISSA
   2.540E-01  8.2E-02  1.00E+00
....X....
   2.793E-01  1.1E-01  2.00E+00
.....X.....
  -5.128E-01  1.9E-01  3.00E+00  .........X..........

  -4.242E-01  4.7E-02  4.00E+00            ...X..

  -3.523E-02  1.6E-01  5.00E+00                          ........X........

   1.071E-01  6.2E-02  6.00E+00                                      ...X...

   4.929E-01  6.3E-02  7.00E+00
       ...X...
   6.108E-01  6.6E-02  8.00E+00
            ...X...
   1.327E+00  9.6E-02  9.00E+00
                                             ....X
  -7.609E-01  9.3E-02  1.00E+01X....

  -1.470E-01  4.1E-02  1.10E+01                           ..X..

  -2.136E-01  6.0E-02  1.20E+01                       ...X...

  -1.536E-01  5.0E-02  1.30E+01                          ...X..

  -2.460E-01  6.0E-02  1.40E+01                     ...X...

   1.088E-02  8.7E-02  1.50E+01                                ....X.....

   3.717E-01  1.9E-01  1.60E+01
..........X.........

                       HELIX   BETA-SHEET    REMAINDER                 SCALE
FACTOR
 FRACTION               0.00         0.68         0.32
0.960
 STANDARD ERROR       9.2E-09      2.7E-02      1.7E-02
0(FOR ALPHA/S(1) = 4.31E-04) PRUNS = 0.0011          PUNCOR =  0.0000  0.7938
0.0001  0.0000  0.0028


           TEST DATA SET 1 - FOR CD PACKAGE


      ALPHA    ALPHA/S(1)     OBJ. FCTN.       VARIANCE      STD. DEV.    DEG
FREEDOM    PROB1 TO REJECT    PROB2 TO REJECT
   1.29E-02      3.16E-01    5.66608E+04    7.62262E+03      1.232E+01
1.741            1.000              1.000

    ORDINATE    ERROR  ABSCISSA
  -3.621E-02  2.9E-03  1.00E+00X...

   1.603E-02  1.2E-03  2.00E+00
         .X.
   2.704E-02  1.5E-03  3.00E+00
                     ..X..
   1.759E-02  2.5E-03  4.00E+00
         ...X...
   1.154E-02  1.1E-03  5.00E+00
    .X.
   3.913E-02  1.7E-03  6.00E+00
                            ..X..
```

```
   5.015E-02  1.8E-03  7.00E+00
                                               ..X
   2.245E-02  1.5E-03  8.00E+00
                .X..
   4.588E-02  2.2E-03  9.00E+00
                                          ...X..
   2.064E-02  1.1E-03  1.00E+01
              .X..
   1.448E-02  1.9E-03  1.10E+01
      ..X..
   8.770E-03  2.1E-03  1.20E+01
...X..
   3.228E-02  2.0E-03  1.30E+01
                          ...X..
   2.140E-02  3.1E-03  1.40E+01
             ...X....
   7.158E-03  1.3E-03  1.50E+01
..X.
   2.732E-02  1.6E-03  1.60E+01
                     ..X..

                       HELIX   BETA-SHEET    REMAINDER                SCALE
FACTOR
 FRACTION                0.18         0.35         0.48
0.326
 STANDARD ERROR      1.5E-02      1.1E-02      1.9E-02
0(FOR ALPHA/S(1) = 3.16E-01) PRUNS = 0.0000         PUNCOR =  0.0000  0.0000
0.0000  0.0000  0.0000


           TEST DATA SET 1 - FOR CD PACKAGE


      ALPHA    ALPHA/S(1)     OBJ. FCTN.       VARIANCE      STD. DEV.    DEG
FREEDOM    PROB1 TO REJECT    PROB2 TO REJECT
   4.52E-05      1.11E-03    9.40368E+01    5.20334E+01      1.158E+00
13.211              0.329              0.996

    ORDINATE    ERROR  ABSCISSA
   9.893E-02  5.7E-02  1.00E+00                                    ...X....

   1.904E-01  7.2E-02  2.00E+00
.....X....
  -2.504E-01  8.6E-02  3.00E+00             ......X.....

  -3.766E-01  3.9E-02  4.00E+00         ..X..

   1.149E-01  1.0E-01  5.00E+00                                  ......X......

   1.351E-01  4.6E-02  6.00E+00                                       ..X...

   4.127E-01  4.3E-02  7.00E+00
     ...X..
   4.942E-01  5.7E-02  8.00E+00
         ....X...
   1.114E+00  8.1E-02  9.00E+00
                                             ....X
  -5.723E-01  7.5E-02  1.00E+01X....

  -9.701E-02  3.6E-02  1.10E+01                          ..X..

  -1.825E-01  4.0E-02  1.20E+01                    ...X..

  -1.867E-01  3.8E-02  1.30E+01                    ..X...

  -2.543E-01  5.0E-02  1.40E+01               ...X...
```

```
   1.201E-01  7.0E-02  1.50E+01                                    .....X....

   1.519E-01  8.5E-02  1.60E+01                                     .....X.....


                       HELIX   BETA-SHEET    REMAINDER                SCALE
FACTOR
 FRACTION               0.00         0.65         0.35
0.912
 STANDARD ERROR      9.0E-09      2.7E-02      1.6E-02
0(FOR ALPHA/S(1) = 1.11E-03) PRUNS = 0.0044         PUNCOR =  0.0000  0.7252
0.0045  0.0002  0.0015


           TEST DATA SET 1 - FOR CD PACKAGE


      ALPHA    ALPHA/S(1)     OBJ. FCTN.       VARIANCE      STD. DEV.    DEG
FREEDOM    PROB1 TO REJECT    PROB2 TO REJECT
   1.16E-04      2.84E-03    2.26031E+02    1.25481E+02      1.756E+00
11.321             1.000              1.000

    ORDINATE    ERROR  ABSCISSA
  -1.042E-01  3.2E-02  1.00E+00              ....X...

   1.081E-01  5.3E-02  2.00E+00                                    ......X......

  -8.943E-02  3.4E-02  3.00E+00               ....X....

  -2.105E-01  3.4E-02  4.00E+00   ....X....

   1.687E-01  5.0E-02  5.00E+00
......X.....
   1.192E-01  4.7E-02  6.00E+00
.....X......
   3.510E-01  3.2E-02  7.00E+00
               ....X....
   2.292E-01  4.2E-02  8.00E+00
 .....X....
   6.253E-01  6.2E-02  9.00E+00
                                           ......X
  -2.754E-01  5.8E-02  1.00E+01X......

   2.995E-02  3.5E-02  1.10E+01                             ....X....

  -1.707E-01  3.3E-02  1.20E+01       ....X....

  -1.640E-01  3.3E-02  1.30E+01        ....X....

  -1.363E-01  4.3E-02  1.40E+01          .....X.....

   2.577E-01  5.1E-02  1.50E+01
   ......X.....
   2.730E-02  3.6E-02  1.60E+01                             ....X....


                       HELIX   BETA-SHEET    REMAINDER                SCALE
FACTOR
 FRACTION               0.00         0.61         0.39
0.766
 STANDARD ERROR      2.5E-09      3.7E-02      2.3E-02
0(FOR ALPHA/S(1) = 2.84E-03) PRUNS = 0.0002         PUNCOR =  0.0046  0.2342
0.4435  0.0624  0.0234


           TEST DATA SET 1 - FOR CD PACKAGE
```

```
      ALPHA    ALPHA/S(1)     OBJ. FCTN.       VARIANCE      STD. DEV.    DEG
FREEDOM    PROB1 TO REJECT    PROB2 TO REJECT
   2.98E-04      7.29E-03    4.78506E+02    3.13107E+02      2.697E+00
8.944              1.000              1.000

    ORDINATE    ERROR  ABSCISSA
  -1.115E-01  1.8E-02  1.00E+00....X.....

   6.690E-02  3.3E-02  2.00E+00
.........X.........
  -2.115E-02  1.7E-02  3.00E+00                      .....X....

  -9.771E-02  2.7E-02  4.00E+00........X.......

   9.680E-02  2.8E-02  5.00E+00
  .......X.......
   9.678E-02  3.4E-02  6.00E+00
.........X.........
   2.510E-01  2.5E-02  7.00E+00
                                           ......X
   5.737E-02  3.3E-02  8.00E+00
........X.........
   2.252E-01  3.2E-02  9.00E+00
                                   .........X......
  -7.788E-02  3.5E-02  1.00E+01    .........X.........

   5.183E-02  3.2E-02  1.10E+01
........X........
  -1.285E-01  2.7E-02  1.20E+01X.......

  -4.950E-02  2.6E-02  1.30E+01           .......X.......

  -5.230E-03  3.0E-02  1.40E+01                      ........X........

   1.691E-01  3.2E-02  1.50E+01
                   .........X........
  -8.169E-03  1.9E-02  1.60E+01                        .....X.....


                         HELIX   BETA-SHEET    REMAINDER              SCALE
FACTOR
 FRACTION                 0.00         0.57         0.43
0.515
 STANDARD ERROR      3.9E-09      4.7E-02      3.5E-02
0(FOR ALPHA/S(1) = 7.29E-03) PRUNS = 0.0004         PUNCOR =  0.1726  0.1866
0.7112  0.4809  0.1978
```

TEST DATA SET 1 - FOR CD PACKAGE

```
      ALPHA    ALPHA/S(1)     OBJ. FCTN.       VARIANCE      STD. DEV.    DEG
FREEDOM    PROB1 TO REJECT    PROB2 TO REJECT
   7.65E-04      1.87E-02    8.96455E+02    5.57469E+02      3.499E+00
6.468              1.000              1.000

    ORDINATE    ERROR  ABSCISSA
  -4.438E-02  6.9E-03  1.00E+00    ...X....

   3.759E-02  1.5E-02  2.00E+00
.......X.......
   6.977E-03  1.0E-02  3.00E+00                           .....X.....

  -4.342E-02  1.4E-02  4.00E+00 .......X.......
```

```
   3.250E-02  1.2E-02  5.00E+00
.....X......
   8.295E-02  1.7E-02  6.00E+00
            .........X........
   1.411E-01  1.3E-02  7.00E+00
                                             ......X
   1.756E-04  1.7E-02  8.00E+00                        ........X.........

   7.571E-02  1.1E-02  9.00E+00
           ......X......
   1.992E-02  1.2E-02  1.00E+01                                    ......X......

   2.179E-02  1.7E-02  1.10E+01
........X.........
  -5.997E-02  1.3E-02  1.20E+01X......

   5.833E-03  1.6E-02  1.30E+01                           ........X........

   3.426E-03  1.3E-02  1.40E+01                            ......X......

   3.222E-02  1.4E-02  1.50E+01
......X.......
   2.672E-03  1.1E-02  1.60E+01                            ......X.....


                       HELIX   BETA-SHEET    REMAINDER                SCALE
FACTOR
 FRACTION                0.00         0.52         0.48
0.315
 STANDARD ERROR      2.1E-09      3.8E-02      4.0E-02
0(FOR ALPHA/S(1) = 1.87E-02) PRUNS = 0.0004         PUNCOR =  0.0806  0.3471
0.9966  0.4547  0.3287
```

TEST DATA SET 1 - FOR CD PACKAGE

```
      ALPHA    ALPHA/S(1)     OBJ. FCTN.       VARIANCE      STD. DEV.    DEG
FREEDOM    PROB1 TO REJECT    PROB2 TO REJECT
   1.96E-03      4.80E-02    2.30917E+03    7.70873E+02      4.037E+00
4.690              1.000              1.000

    ORDINATE    ERROR  ABSCISSA
  -2.960E-02  4.1E-03  1.00E+00X....

   1.675E-02  5.2E-03  2.00E+00
......X.....
   8.437E-03  6.4E-03  3.00E+00                                    ......X.......

  -6.107E-03  6.9E-03  4.00E+00                 .......X.......

   1.635E-02  2.9E-03  5.00E+00
...X...
   4.328E-02  5.2E-03  6.00E+00
                  ......X.....
   6.875E-02  7.3E-03  7.00E+00
                                          .......X
   6.885E-03  5.7E-03  8.00E+00                               ......X.....

   3.925E-02  3.2E-03  9.00E+00
                ...X....
   1.423E-02  2.3E-03  1.00E+01                                                ..X..

   1.051E-02  6.5E-03  1.10E+01                                    ......X.......

  -1.482E-02  6.8E-03  1.20E+01        .......X......
```

```
   1.522E-02  8.2E-03  1.30E+01
........X........
   7.060E-03  4.3E-03  1.40E+01                               .....X....

   9.658E-03  4.4E-03  1.50E+01                                  ....X.....

   7.216E-03  6.3E-03  1.60E+01                              ......X......


                       HELIX   BETA-SHEET    REMAINDER                SCALE
FACTOR
 FRACTION               0.08         0.43         0.49
0.213
 STANDARD ERROR      1.4E-02      2.6E-02      3.3E-02
0(FOR ALPHA/S(1) = 4.80E-02) PRUNS = 0.0000         PUNCOR =  0.0141  0.2615
0.8582  0.7258  0.6617



           TEST DATA SET 1 - FOR CD PACKAGE


      ALPHA    ALPHA/S(1)     OBJ. FCTN.       VARIANCE      STD. DEV.    DEG
FREEDOM    PROB1 TO REJECT    PROB2 TO REJECT
   5.04E-03      1.23E-01    1.04713E+04    1.14500E+03      4.833E+00
2.985              1.000              1.000

    ORDINATE    ERROR  ABSCISSA
  -3.260E-02  2.8E-03  1.00E+00X...

   1.198E-02  1.6E-03  2.00E+00
..X..
   1.602E-02  2.2E-03  3.00E+00
    ...X..
   5.584E-03  3.2E-03  4.00E+00                                        ...X....

   1.064E-02  1.0E-03  5.00E+00
.X.
   3.371E-02  1.6E-03  6.00E+00
                           ..X..
   5.255E-02  2.8E-03  7.00E+00
                                                       ...X
   1.386E-02  2.2E-03  8.00E+00
 ...X...
   3.627E-02  1.5E-03  9.00E+00
                               .X..
   1.363E-02  9.0E-04  1.00E+01
   .X.
   9.941E-03  2.8E-03  1.10E+01
...X....
  -3.099E-03  2.9E-03  1.20E+01                              ...X....

   2.436E-02  3.0E-03  1.30E+01
             ...X....
   7.896E-03  2.3E-03  1.40E+01
...X...
   6.712E-03  1.5E-03  1.50E+01                                           ..X.

   1.589E-02  2.3E-03  1.60E+01
    ..X...

                       HELIX   BETA-SHEET    REMAINDER                SCALE
FACTOR
 FRACTION               0.12         0.39         0.49
0.223
 STANDARD ERROR      1.1E-02      1.3E-02      2.1E-02
0(FOR ALPHA/S(1) = 1.23E-01) PRUNS = 0.0000         PUNCOR =  0.0001  0.0042
0.0517  0.2179  0.4673
```

FhuAExt-POlymersoem

1CONTIN 2DP (MAR 84) (CD-1)   TEST DATA SET 1 - FOR CD PACKAGE
CHOSEN SOLUTION

WEIGHTED RESIDUALS (ALPHA/S(1)= 1.11E-03) MAX=U= 2.9E+00 MIN=L=-1.6E+00
(PRUNS= 0.0044) PUNCOR= 0.0000 0.7252 0.0045 0.0002 0.0015

```
 U---------+---------+---------+---------+---------+-*


                              *
  *                          *
                                      **
   *                           *
    *        * **      *    *                    *
     *        *  *   **         *    *     *   *
      *     *                           *         *
 0---------*------------*-------------------**--*--**-
       *  *       *        *
                                 *        *
         *          *                    *    *
        *          *     **         *
 L---------+---------+---------+--**-----+---------+--
                     20                  40                  60
80                 100                 120
```

0PLOT OF DATA (O) AND FIT TO DATA (X). ORDINATES LISTED ARE FIT VALUES.

```
    ORDINATE  ABSCISSA
  -1.464E+02  2.40E+02
             X           O
  -1.319E+02  2.39E+02
              X       O
  -1.742E+02  2.38E+02
           X    O
  -2.483E+02  2.37E+02
     X   O
  -3.225E+02  2.36E+02
X O
  -3.778E+02  2.35E+02                                                  O  X

  -4.201E+02  2.34E+02                                          O       X

  -4.679E+02  2.33E+02                                        O     X

  -5.407E+02  2.32E+02                                     O  X

  -5.989E+02  2.31E+02                                  O X

  -6.446E+02  2.30E+02                                X O

  -6.532E+02  2.29E+02                               X     O

  -6.479E+02  2.28E+02                                X   O

  -6.678E+02  2.27E+02                              X    O

  -7.174E+02  2.26E+02                          X   O

  -7.437E+02  2.25E+02                        X  O

  -7.764E+02  2.24E+02                   O X

  -7.752E+02  2.23E+02            O        X

  -8.043E+02  2.22E+02            O      X
```

```
  -8.749E+02  2.21E+02             X  O
  -9.208E+02  2.20E+02         X    O
  -9.185E+02  2.19E+02          X   O
  -8.618E+02  2.18E+02              *
  -8.150E+02  2.17E+02         O        X
  -8.387E+02  2.16E+02       O        X
  -9.229E+02  2.15E+02      O  X
  -9.959E+02  2.14E+02   X     O
  -1.043E+03  2.13E+02X          O
  -1.013E+03  2.12E+02  X            O
  -9.164E+02  2.11E+02          X       O
  -7.980E+02  2.10E+02                   X   O
  -7.012E+02  2.09E+02                       O   X
  -6.193E+02  2.08E+02                        O         X
  -6.442E+02  2.07E+02                     O          X
  -7.283E+02  2.06E+02                 O       X
  -8.499E+02  2.05E+02               X  O
  -8.846E+02  2.04E+02            X         O
  -8.305E+02  2.03E+02                 X        O
  -6.539E+02  2.02E+02                               XO
  -4.324E+02  2.01E+02                                           O     X
  -2.708E+02  2.00E+02
O   X
  -1.968E+02  1.99E+02
         X   O
  -2.463E+01  1.98E+02
                       *
   1.348E+02  1.97E+02
                                    *
   2.456E+02  1.96E+02
                                       O     X
   2.723E+02  1.95E+02
                                               X O
   1.722E+02  1.94E+02
                                       *
   5.910E+01  1.93E+02
                              X   O
   5.167E+01  1.92E+02
                             X O
   7.750E+01  1.91E+02
                              OX
   1.210E+02  1.90E+02
                                   *
   9.118E-01  0.00E+00
                         *
1CONTIN VERSION 2DP (MAR 1984) ( CD-1  PACKAGE)
++++++++++++++++++++++++++++++++  CHOSEN SOLUTION
```

```
++++++++++++++++++++++++++++++++

           TEST DATA SET 1 - FOR CD PACKAGE


      ALPHA    ALPHA/S(1)     OBJ. FCTN.       VARIANCE      STD. DEV.    DEG
FREEDOM    PROB1 TO REJECT    PROB2 TO REJECT
   4.52E-05      1.11E-03    9.40368E+01    5.20334E+01      1.158E+00
13.211             0.329              0.996

    ORDINATE    ERROR  ABSCISSA
   9.893E-02  5.7E-02  1.00E+00                                            ...X....

   1.904E-01  7.2E-02  2.00E+00
.....X....
  -2.504E-01  8.6E-02  3.00E+00             ......X.....

  -3.766E-01  3.9E-02  4.00E+00         ..X..

   1.149E-01  1.0E-01  5.00E+00                                          ......X......

   1.351E-01  4.6E-02  6.00E+00                                                ..X...

   4.127E-01  4.3E-02  7.00E+00
     ...X..
   4.942E-01  5.7E-02  8.00E+00
         ....X...
   1.114E+00  8.1E-02  9.00E+00
                                             ....X
  -5.723E-01  7.5E-02  1.00E+01X....

  -9.701E-02  3.6E-02  1.10E+01                         ..X..

  -1.825E-01  4.0E-02  1.20E+01                   ...X..

  -1.867E-01  3.8E-02  1.30E+01                   ..X...

  -2.543E-01  5.0E-02  1.40E+01              ...X...

   1.201E-01  7.0E-02  1.50E+01                                            .....X....

   1.519E-01  8.5E-02  1.60E+01                                             .....X.....

                       HELIX   BETA-SHEET    REMAINDER                SCALE
FACTOR
 FRACTION               0.00         0.65         0.35
0.912
 STANDARD ERROR      9.0E-09      2.7E-02      1.6E-02
```
